# Pan-cancer analysis of IFN-γ with possible immunotherapeutic significance: a verification of single-cell sequencing and bulk omics research

**DOI:** 10.3389/fimmu.2023.1202150

**Published:** 2023-08-14

**Authors:** Xiaoying Wei, Hanyi Ruan, Yan Zhang, Tianyu Qin, Yujie Zhang, Yan Qin, Wei Li

**Affiliations:** ^1^ Department of Health Management, The People’s Hospital of Guangxi Zhuang Autonomous Region and Research Center of Health Management, Guangxi Academy of Medical Sciences, Nanning, China; ^2^ Department of Oncology, Guangxi Medical University Cancer Hospital, Nanning, China

**Keywords:** IFN-γ, tumor microenvironment, immunotherapy, pan-cancer, single-cell transcriptome sequencing

## Abstract

**Background:**

Interferon-gamma (IFN-γ), commonly referred to as type II interferon, is a crucial cytokine that coordinates the tumor immune process and has received considerable attention in tumor immunotherapy research. Previous studies have discussed the role and mechanisms associated with IFN-γ in specific tumors or diseases, but the relevant role of IFN-γ in pan-cancer remains uncertain.

**Methods:**

TCGA and GTEx RNA expression data and clinical data were downloaded. Additionally, we analyzed the role of IFN-γ on tumors by using a bioinformatic approach, which included the analysis of the correlation between IFN-γ in different tumors and expression, prognosis, functional status, TMB, MSI, immune cell infiltration, and TIDE. We also developed a PPI network for topological analysis of the network, identifying hub genes as those having a degree greater than IFN-γ levels.

**Result:**

IFN-γ was differentially expressed and predicted different survival statuses in a majority of tumor types in TCGA. Additionally, IFN-γ expression was strongly linked to factors like infiltration of T cells, immune checkpoints, immune-activating genes, immunosuppressive genes, chemokines, and chemokine receptors, as well as tumor purity, functional statuses, and prognostic value. Also, prognosis, CNV, and treatment response were all substantially correlated with IFN-γ-related gene expression. Particularly, the IFN-γ-related gene STAT1 exhibited the greatest percentage of SNVs and the largest percentage of SNPs in UCEC. Elevated expression levels of IFN-γ-related genes were found in a wide variety of tumor types, and this was shown to be positively linked to drug sensitivity for 20 different types of drugs.

**Conclusion:**

IFN-γ is a good indicator of response to tumor immunotherapy and is likely to limit tumor progression, offering a novel approach for immunotherapy’s future development.

## Introduction

Cancer is one of the most feared diseases of the 21st century and has been rapidly increasing in prevalence over the past few decades. This can be attributed to changes in our lifestyles, habits, and the fact that people are living longer. As a result, cancer has become a major threat to human life and health. In the field of cancer treatment, there is now a strong focus on preserving the immune system, which has led to numerous advancements and breakthroughs in the area of immunotherapy. Some of the advanced immunotherapeutic strategies being employed today include the transfer of isolated activated T cells, the use of immunomodulatory monoclonal antibodies (MABs), and the development of cancer vaccines (1).

Cytokines (CK) are a class of proteins with a small molecular weight (typically <30kDa) and diverse biological functions. They are produced and released by immune cells as well as certain non-immune cells (such as fibroblasts, epidermal cells, and endothelial cells) in response to stimulation (2, 3). CKs are crucial components of the immune system and play a vital role in regulating both pathological conditions (such as cancer and autoimmune diseases) and maintaining physiological immunological balance (4, 5). CKs can be categorized into groups such as tumor necrosis factor (TNF), interferon (IFN), colony-stimulating factor (CSF), and interleukin (IL).When CKs bind to their respective receptor subunits, signaling is initiated through the formation of dimers or oligomers. This activation leads to the stimulation of pathways involving signal transducers and activators of transcription (STATs) and Janus kinases (JAKs). Additionally, specific gene expression programs and biological processes are activated (6, 7). The clustering of receptors triggers the activation of various kinases, which then phosphorylate tyrosine and serine residues in the cytoplasmic structural domain of the receptor. This phosphorylation event further activates transcriptional regulators, facilitating nuclear translocation and modulation of gene expression. Consequently, these processes exert the corresponding biological effects (8, 9).

Interferon-γ (IFN-γ), the sole member of the type II interferon family, plays a critical role as a cytokine. It is released by activated T lymphocytes, natural killer cells (NK), and γδT cells within the tumor microenvironment (TME). IFN-γ exhibits cytostatic, pro-apoptotic, and immune-inducing effects. Moreover, it performs a fundamental function in coordinating the anti-tumor immune process (10, 11). In addition to its function in the activation of cellular immunity and the enhancement of anti-tumor immunity, active IFN-γ signaling is linked to apoptosis and the arrest of the cell cycle in human cancer cells, both of which have the potential to have a direct impact on the fight against cancer (12). The role of IFN-γ in anti-tumor activities is best illustrated by the process known as cancer immunoediting. IFN-γ can induce multiple immunomodulatory pathways to achieve antitumor effects during the elimination phase of immunoediting as well as to maintain immune homeostasis (13). However, malignant tumor cells can also use IFN-γ as an inducer to suppress anti-tumor immunity and achieve immune escape of tumor cells *in vivo* (13, 14). Numerous research reports have demonstrated that active IFN-γ signaling is a characteristic that is shared by most tumors in the IFN-γ-tumor relationship through targeting cytotoxic T lymphocyte-associated antigen-4 (CTLA-4) and programmed cell death protein 1/programmed cell death 1 ligand 1 (PD-1/PD-L1) antibodies when subjected to immune checkpoint blockade (ICB) (15, 16). IFN-γ promotes the expression of the immunosuppressive metabolite indoleamine2,3-dioxygenase (IDO) in tumor cells and host bone marrow cells by driving the upregulation of PD-L1 in these cells, thus suppressing tumor-specific T cells and contributing to the development of an immunosuppressive TME (13).

Although the anti-cancer effects of IFN-γ have been demonstrated in various tumor studies, there is still a lack of research exploring its properties and mechanisms in pan-cancer. Additionally, there has been limited investigation into the positive and negative effects of IFN-γ in the anti-tumor process. To address this gap, we conducted a pan-cancer analysis using the Genotype-Tissue Expression (GTEx) and the Cancer Genome Atlas (TCGA) databases. This analysis focused on genes associated with IFN-γ across a range of tumors, examining their expression levels, prognostic outcomes, immune infiltration, tumor purity, single-cell levels, and tumor markers. By doing so, we aimed to provide valuable insights into the potential application of IFN-γ in tumor immunotherapy, expanding our understanding of its involvement in anti-cancer mechanisms.

## Method

### Data collection

We downloaded TCGA and GTEx RNA expression and clinical data by using the UCSC XENA database (http://xena.ucsc.edu/). TCGA (https://portal.gdc.cancer.gov/) is a platform with a sample size of over 10,000 and contains data on 33 common tumors and follow-up data (17, 18). **Supplementary Table 1** displayed the full and abbreviated tumor names. TCGA was searched for methylation data and copy number variation (CNV). From the TCGA dataset, we retrieved RNA-Seq data that was presented in the form of transcripts per million (TPM). Additionally, we used the GTEx dataset for gene expression analysis in non-cancer tissues (19).

### Evaluation of IFN-γ scores

IFN-γ-related gene was derived from Ayers et al (20). In their study, the IFN-γ 10 gene signature was identified based on data from different clinical studies using a learning-validation model. Calculation of IFN-γ scores based on single-sample gene-set enrichment analysis (ssGSEA) for the quantification of expression levels of these genes in each cancer (21). ssGSEA uses a method similar to GSEA enrichment analysis in which the enrichment score of the target gene set is calculated by ranking the target genes among the total genes. ssGSEA converts the gene expression profile of a single sample into a gene set enrichment profile. The enrichment score of a gene set represents the activity level of a biological process that is synergistically upregulated or downregulated by the members of the gene set. This transformation allows researchers to characterize cell states in terms of the activity levels of biological processes and pathways, rather than by the expression levels of individual genes (22).

### Construction of IFN-γ regulation Network and protein-protein interaction (PPI) analysis

Analysis of protein-protein interactions (PPI) was conducted on the IFN-γ-related genes after they were imported into the STRING database (https://string-db.org/). After downloading the txt file, an Excel copy of it was made for annotation purposes, after which it was imported into the Cytoscape program to develop the PPI network for the core genes. Cytoscape’s network analysis feature was utilized to examine the topology of the network, and genes with degrees greater than IFN-γ were considered hub genes.

### The analysis of IFN-γ function at the single-cell level

We investigated the association of IFN-γ with functional status in many malignancies utilizing the CancerSEA database. Single-cell analysis of 14 functional statuses of 10 IFN-γ-related genes across tumor types was conducted utilizing the Cancer Single Cell State Atlas (CancerSEA) database (http://biocc.hrbmu.edu.cn/CancerSEA/).

### Single-cell transcriptome sequencing data analysis

Single-cell transcriptome sequencing data (GSE152938) was downloaded form GEO database. Prior literature has outlined the steps used to prepare single-cell suspensions (23). In brief, cold Hank’s Balanced Salt Solution was utilized to transport freshly isolated tumor samples from the operating room to the lab (HBSS; Gibco, C11875500BT). Afterward, the samples were rinsed and sliced into 2-4 mm sections. For 30 minutes, several species of tissue were gently agitated in a digesting solution comprised of HBSS at 37°C. Before single-cell sequencing, samples were washed and filtered to remove red blood cells and determine cell viability. Two samples of kidney clear cell carcinoma (KIRC) were obtained from patients who underwent radical nephrectomy. Hiseq X10 (Illumina, San Diego, California) with standard settings was utilized to sequence all the samples. CellRanger (v3.0.2) was utilized to transform preliminary sequencing data (.bcl) into FASTQ files. To perform quality control (QC) and secondary analysis, we employed the R programming language (v3.5.2) together with the Seurat R package (v3.1.1). The GEO database (GSE152938) contains the datasets derived by single-cell sequencing (24).

### Paraffin-embedded tissue collection

The matched malignancies and paracancerous tissues used in this study were derived from a total of 43 patients with breast cancer. Patients received a definite breast cancer diagnosis but had not yet undergone any kind of chemotherapy or radiotherapy. All patients were granted their written consent to participate. The affiliated Cancer Hospital of Guangxi Medical University’s Ethics and Anthropology Committee granted its approval to the present research. All procedures and tests were carried out in conformity with all applicable guidelines and regulations.

### Immunohistochemical staining of paraffin sections

The immunohistochemistry detection kit (EliVision plus) and DAB staining kit were purchased from Maixin Biotechnology Company in Fuzhou, China. Formalin was utilized to preserve all the tumor samples. To prepare the tissues for staining, they were first sectioned to a thickness of 5 micrometers and then put on glass slides, followed by routine dehydration, paraffin embedding, and consecutive sectioning with a thickness of 4μm. Deparaffinization was done using xylene, followed by gradient ethanol hydration. EDTA high-temperature high-pressure antigen retrieval, DAB staining, and counterstaining with hematoxylin were performed. The primary antibody was diluted at a concentration of 1:1000. Immunohistochemical staining was performed using the EnVision two-step method, and all experimental procedures strictly followed the instructions provided with the kit.

### Survival analysis

Utilizing the R software, we carried out analyses of univariate Cox regression and Kaplan-Meier (KM) survival. The relevance of IFN-γ expression to patients with various cancers was assessed by using measurements of progression-free interval (PFI), disease-specific survival (DSS), and overall survival (OS) (25). Furthermore, both KM curves and univariate Cox proportional hazards regression were utilized to derive p-values, 95% confidence intervals (CIs), and hazard ratios (HRs) (26).

### Correlation analysis between IFN-γ expression and immunity

Both tumor mutational burden (TMB) and microsatellite instability (MSI) have been proven in previous research to play a role in the prevention and treatment of tumors (27). TMB is a biological marker of immune response that characterizes the number of mutations that have occurred in tumor cells (28), calculated as the total number of errors in somatic gene coding, base substitution, gene insertions, or deletion that can be identified per million bases (29). The TMB score was determined by dividing the sum of mutations by the size of the exome (the size of an exome was determined at 38 MB). MSI, induced by MMR defects, is related to patient prognosis (30). Data on somatic mutations were collected from TCGA (https://tcga.xenahubs.net) and used to compute MSI scores for all samples.

Furthermore, utilizing the TIMER database (http://cistrome.org/TIMER/), we examined the link between IFN-γ and tumor-infiltrating immune cells (TIICs). Through the use of ssGSEA, we studied how IFN-γ is linked to other immune-related factors such TIICs, immune-activating genes, immune suppressor genes, chemokines, and chemokine receptors. The immune score is a representation of the number of immune cells that have infiltrated the tumor tissue.

### Tumor immune dysfunction and exclusion score analysis

TIDE is a mathematical framework that integrates and models data from 33,197 samples collected from 189 human cancer studies. When applied to malignancies, TIDE simulates the immune evasion mechanism by dampening the function of T cells in cancers with high cytotoxic T cell (CTL) infiltration and inhibiting the infiltration of T cells in tumors with lower CTL infiltration (31, 32). Following the tagging of defective markers on T cells, how the expression of certain genes in the tumor interacts with the amount of CTL infiltration was analyzed to determine how it will affect patient survival (33). TIDE is an effective predictor of ICBF response, and patients exhibiting elevated TIDE scores have a higher risk of the tumor evading the immune system. Consequently, they have a low likelihood of responding favorably to the ICBF scheme.

### Drug sensitivity analysis

The Genomics of Drug Sensitivity in Cancer (GDSC) database was searched to obtain the data on the cell lines (n = 860), genes (n = 17419), and small molecules (n = 265). Using the methodology developed by Rees et al., we investigated the degree to which gene expression is correlated with drug responsiveness (34). The half maximum inhibitory concentration (IC50) values for medications as well as gene expression patterns for each tumor cell line were obtained from the GDSC. Calculations were made to determine the Pearson correlation coefficients between the transcript levels and the IC50 value (24).

### Statistical analysis

The raw data obtained from TCGA and GTEx RNA were subjected to log2 transformation for normalization before further analysis. The Spearman correlation test was performed to assess the associations between gene expressions, and a significance level of P < 0.05 was used as the threshold for determining significant correlations. The Student’s t-test was utilized to compare the differences in gene expression levels between normal and cancerous tissues. Kaplan-Meier (KM) survival curves were employed to evaluate the prognostic significance of the analyzed indexes. Cox proportional risk regression models were used to calculate adjusted risk ratios. A significance level of P < 0.05 was considered statistically significant.

## Result

### Differentially expressed IFN-γ-related genes in pan-carcinoma and their effect on prognosis

**Figure 1:**
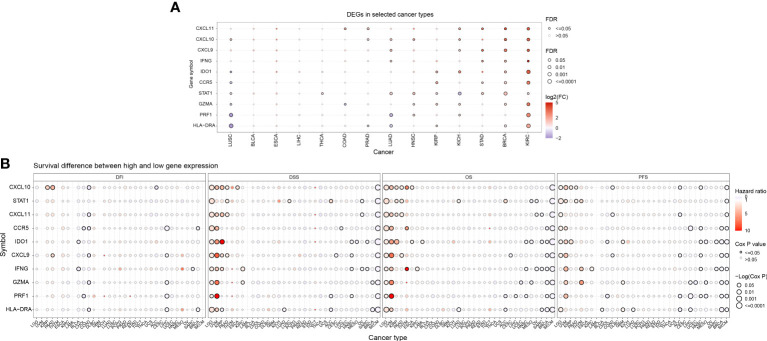
Prognostic significance of differential IFN-γ-related genes (IFN-γ-RGs) in various cancers. **(A)** Expression analysis of IFN-γ-RGs in 33 different types of cancer. Red indicates high expression genes, blue indicates low expression genes. **(B)** Survival differences between high and low gene expression levels. Red indicates high hazard ratio (HR). The size of the circles represents the significance level, with larger circles indicating lower p-values.

The **Supplementary Figure 1** shows the flow chart of this study. Initially, we conducted an analysis of gene expression profiles associated with IFN-γ in various cancers and observed variations in their expression levels across different tumor types. Using a heat map, we examined the expression of 10 IFN-γ-associated genes in 33 distinct cancer types and discovered discrepancies in gene expression within the same tumor as well as across different tumor types. Notably, TGCT, LUSC, LUAD, KIRC, HNSC, DLBC, and CHOL exhibited high expression of the studied genes, while UVM, PCPG, LGG, KICH, and ACC showed low expression (**Figure 1A**). In terms of prognostic implications, we found that high expression of most of the selected 10 genes was associated with shortened progression-free survival (PFS), overall survival (OS), and disease-free survival (DSS) in patients with LGG and UVM, indicating increased risk. Conversely, SKCM patients with high gene expression had higher DSS, OS, and PFS, suggesting a protective effect (all P < 0.05). Additionally, high STAT1 expression was linked to higher DSS and OS in patients with PAAD or ACC (**Figure 1B**). These findings indicate that the expression of IFN-γ-associated genes is correlated with the prognosis of tumor patients, with the correlation depending on the specific tumor type.

### Analysis of IFN-γ-related genes and gene mutations

**Figure 2:**
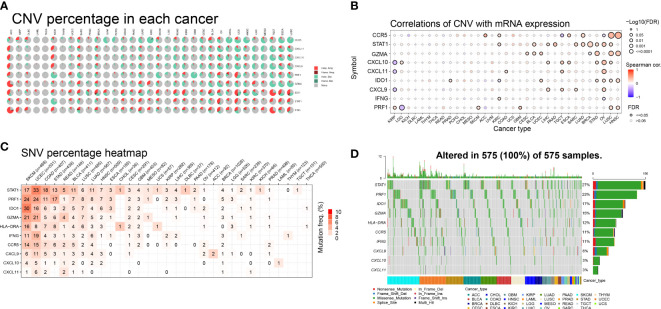
Pie charts illustrating the copy number variation (CNV) distribution of IFN-γ-related genes (IFN-RGs) in 33 different cancers. **(A)** Each CNV pie chart shows the relative frequency of homozygous/heterozygous IFN-γ-RG combinations in each tumor type. Different colored sections represent different CNV types. **(B)** Association between CNV and mRNA expression. The size of the dots represents the statistical significance, with larger dots indicating higher significance. P-values have been adjusted by false discovery rate (FDR) correction. **(C)** Color shading represents the intensity of mutation frequency. The size of the numbers indicates how frequently the associated mutated genes occur in a given tumor sample. No number indicates no mutation of that gene anywhere, “0” indicates no mutation in the coding region of the gene. **(D)** SNV Oncoplot. The side and top bar plots show the number of variations among samples or genes.

To investigate the impact of gene mutations on gene expression, we analyzed the mutation status of IFN-γ-related genes in different tumors. Our study examined the genetic variations of genes associated with IFN-γ in 33 distinct cancers and found that in most malignancies, these genes were associated with copy number variation (CNV). Among the 9 genes we investigated, heterozygous amplification and heterozygous deletion were the most common mutations observed in the 33 distinct cancers. Specifically, heterozygous amplification was the most prevalent CNV type in IDO1, STAT1, and IFNG, while heterozygous deletion was the main CNV type for CCR5, CXCL11, CXCL10, CXCL9, PRF1, and GZMA across 25 tumors. Additionally, in cases of adrenocortical carcinoma (ACC), heterozygous amplification was the primary type of CNV (**Figure 2A**).

Furthermore, we conducted additional research to explore the relationship between relative linear copy number values and the mRNA expression levels of genes associated with IFN-γ. Our data revealed a strong positive correlation between the expression of CCR5, GZMA, IDO1, and PRF1 and CNV in both lung squamous cell carcinoma (LUSC) and head and neck squamous cell carcinoma (HNSC). Conversely, we observed a strong negative correlation between the expression of CXCL9, CXCL10, and CXCL11 and CNV in kidney renal papillary cell carcinoma (KIRP), which is a significant finding (**Figure 2B**). Additionally, we found a strong and favorable correlation between the expression of STAT1 and CNV.

We examined the mutations and types of variation in IFN-γ-related genes in each cancer type and discovered that uterine corpus endometrial carcinoma (UCEC) had the highest percentage (33%) of single nucleotide variations (SNVs) in STAT1, followed by skin cutaneous melanoma (SKCM), colon adenocarcinoma (COAD), stomach adenocarcinoma (STAD), rectum adenocarcinoma (READ), bladder urothelial carcinoma (BLCA), LUSC, and lung adenocarcinoma (LUAD). CASP1 had a high proportion of SNVs in UCEC, SKCM, LUSC, BLCA, and LUAD. In both SKCM and UCEC, the incidence of SNVs was higher in STAT1, PRF1, IDO1, GZMA, HLA-DRA, IFNG, and CCR5. The proportion of SNVs in CXCL10 and CXCL11 was lower (**Figure 2C**). These 10 genes mentioned above were the most common targets of missense mutations in pan-cancer single nucleotide polymorphisms (SNPs). The frequency of gene alterations was highest in patients with UCEC and SKCM, followed by those with STAD, COAD, BLCA, and pancreatic adenocarcinoma (PAAD). STAT1 had the highest proportion of SNPs (27%), followed by PRF1 (23%), IDO1 (17%), and GZMA (15%) (**Figure 2D**). These findings suggest that IFN-γ-related genes have a high frequency of mutations in various tumors and have the potential to be targeted further as therapeutic molecules.

### Differential analysis of methylation of IFN-γ-related genes in pan-cancer

**Figure 3:**
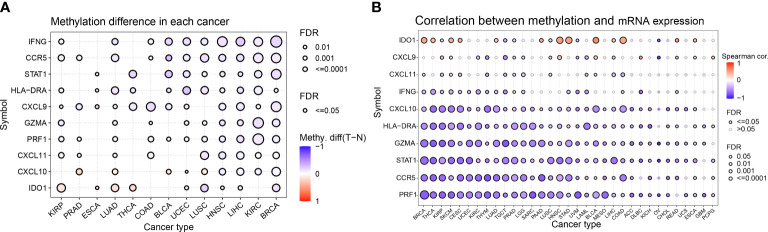
Differential methylation analysis of IFN-γ-related genes in pan-cancer. **(A)** Differential methylation of IFN-RGs in 13 different cancers. Different colors represent different methylation levels, red dots indicate higher methylation levels in cancer, blue dots indicate lower methylation levels. **(B)** Association between methylation and mRNA gene expression. Different colored linkages represent different associations, red dots and blue dots represent positive and negative associations, respectively. P-values have been adjusted by FDR correction.

Abnormal DNA methylation may lead to abnormal gene expression and an increased risk of cancer. We studied the differential methylation of IFN-γ-related genes in 13 distinct types of cancers to learn more about how these genes impact tumorigenesis and uncover the mechanism of aberrant expression of these genes. IDO1 had high methylation levels in KIRP, LUAD, THCA, and ESCA, and significantly low methylation levels in LUSC and BRCA. Among the 10 genes studied, almost all of them showed low methylation levels in BRCA, KIRC, LIHC, and HNSC. Among 13 kinds of tumors, STAT1, IFNG, and CCR5 showed low methylation levels in BRCA, KIRC, LIHC, HNSC, UCEC, and BLCA (**Figure 3A**). For comprehending the relationship between methylation and IFN-γ mRNA expression, we discovered a strong inverse correlation in 31 cancer subtypes. Methylation was inversely associated with the expression of PRF1, CCR5, STAT1, GZMA, HLA-DRA, and CXCL10 in these malignancies. Conversely, methylation was positively linked to IDO1 expression in BRCA, THCA, SKCM, CESC, LUAD, PAAD, HNSC, STAD, BLCA, LIHC, COAD, READ, and ESCA (**Figure 3B**). These results suggest that the aberrant expression of IFN-γ-related genes is partly due to aberrant methylation regulation.

### Differential expression of IFN-γ score and its association with tumor staging

**Figure 4:**
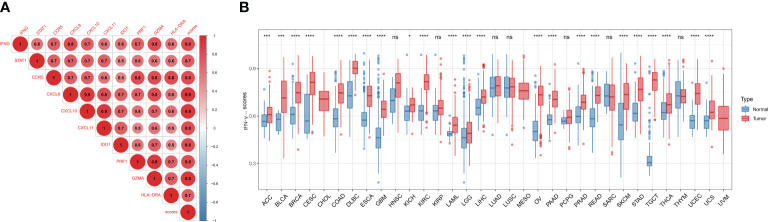
Differential expression of IFN-γ scores. **(A)** Association analysis between IFN-γ scores and IFN-γ-related gene expression. The hue of the colors represents the strength of the association, red dots and blue dots represent positive and negative associations, respectively. **(B)** Comparison of IFN-γ scores between 33 different types of tumors and normal tissues. *p < 0.05, ***p < 0.001, ****p < 0.0001, ns, no significance. P-values have been adjusted by FDR correction.

Firstly, we found a positive link between genes associated with IFN-γ (p<0.05), indicating a close association between IFN-γ-RGs (**Figure 4A**). We also assessed the IFN-γ score between tumor and normal specimens for 33 malignancies using data from GTEx and TCGA. In contrast with normal samples, IFN-γ scores were remarkably elevated in carcinoma tissue samples, including in UCS, BRCA, UCEC, COAD, TGCT, OV, LIHC, LAML, PAAD, KIRC, PRAD, GBM, LGG, READ, ESCA, SKCM, DLBC, STAD, CESC, THCA, BLCA, and ACC. Twenty-two of thirty-three tumors had IFN-γ scores that were greater than those of normal tissues, implying that the inflammatory response in cancerous tissues was greatly enhanced (**Figure 4B**). The IFN-γ scores in pan-cancer at different stages were further investigated. The IFN-γ score was remarkably higher in the early stages of TGTC, HNSC, and COAD (All P < 0.05). The score of IFN-γ was higher in the late stage of KIRP (All P < 0.05). It can be inferred that IFN-γ may be a breakthrough in the early prevention and treatment of TGTC, HNSC, and COAD (**Supplementary Figure 2**).

### Prognostic significance of IFN-γ score in tumor

**Figure 5:**
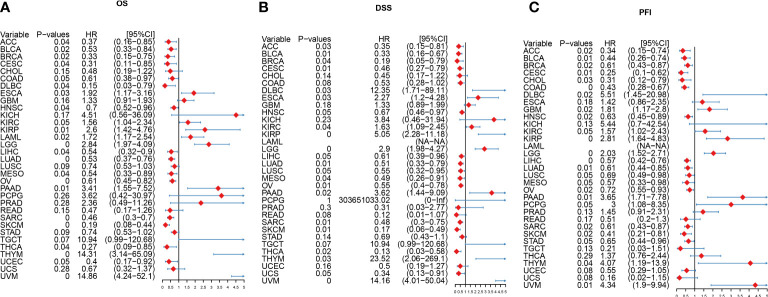
Forest plot of the results from univariate Cox regression analysis for IFN-γ. **(A)** Overall survival (OS). **(B)** Disease-specific survival (DSS). **(C)** Progression-free interval (PFI). P-values have been adjusted by FDR correction.

We explored the predictive significance of IFN-γ in TCGA pan-cancer. Cox Regression analysis demonstrated that IFN-γ served a protective function among patients with SKCM, SARC, OV, MESO, LUAD, LIHC, HNSC, DLBC, CESC, BRCA, BLCA, THCA, and ACC (All P< 0.05, **Figure 5A**). The findings from DSS analysis confirmed the protective function of IFN-γ in BRCA, THCA, SARC, MESO, OV, CESC, LUAD, SKCM, BLCA, and ACC (All P< 0.05, **Figure 5B**). The findings from PFI analysis illustrated the protective function of IFN-γ in BLCA, SKCM, LUAD, CESC, OV, LIHC, COAD, CHOL, BRCA, SARC, HNSC, and ACC (All P< 0.05, **Figure 5C**). Higher IFN-γ scores were linked to improved OS in ESCA, KIRC, LUAD, CESC, SARC, SKCM, STAD, and DLBC, as determined by KM analysis (**Supplementary Figure 3**). In MESO, LUSC, UCS, BRCA, OV, LUAD, CESC, HNSC, SARC, BLCA, SKCM, THCA, and ACC, higher IFN-γ expression was associated with improved OS and DSS (**Supplementary Figure 4**). Additionally, a longer PFI was associated with higher IFN-γ scores in OV, BLCA, STAD, HNSC, SKCM, CESC, LUSC, CHOL, LUAD, MESO, BRCA, COAD, LIHC, AD, and ACC (**Supplementary Figure 5**). From these findings, the IFN-γ score could improve the predictive significance of classical prognostic markers. Moreover, IFN-γ is strongly linked to the prognosis of many types of malignancies, suggesting that it may have a beneficial influence on the prognosis of patients with these tumors. In addition, we performed GSEA analysis of immune activation genes, immune suppression genes, immune checkpoints, chemokines, chemokine receptor gene sets and compared the variability between cancer and para-cancer (**Supplementary Figures 6A–E**). The results showed that the above gene set scores were either high or low in the tumors and lacked results similar to the consistency of IFN-γ-related genes. In addition, we performed 20 random samples of 10 genes each time for the above gene sets to obtain 20 random immune gene sets and perform GSEA analysis. The results were similar to previous results in that no gene sets were observed to have a consistent up- or down-regulation trend across tumors (**Supplementary Figure 6F**). The above results suggest that the expression status of IFN-γ-related genes in tumors is regulated by the biology behind it, and is not a coincidental result that can be obtained by an arbitrary set of immune genes.

### Definition of hub genes and immunohistochemistry verification

**Figure 6:**
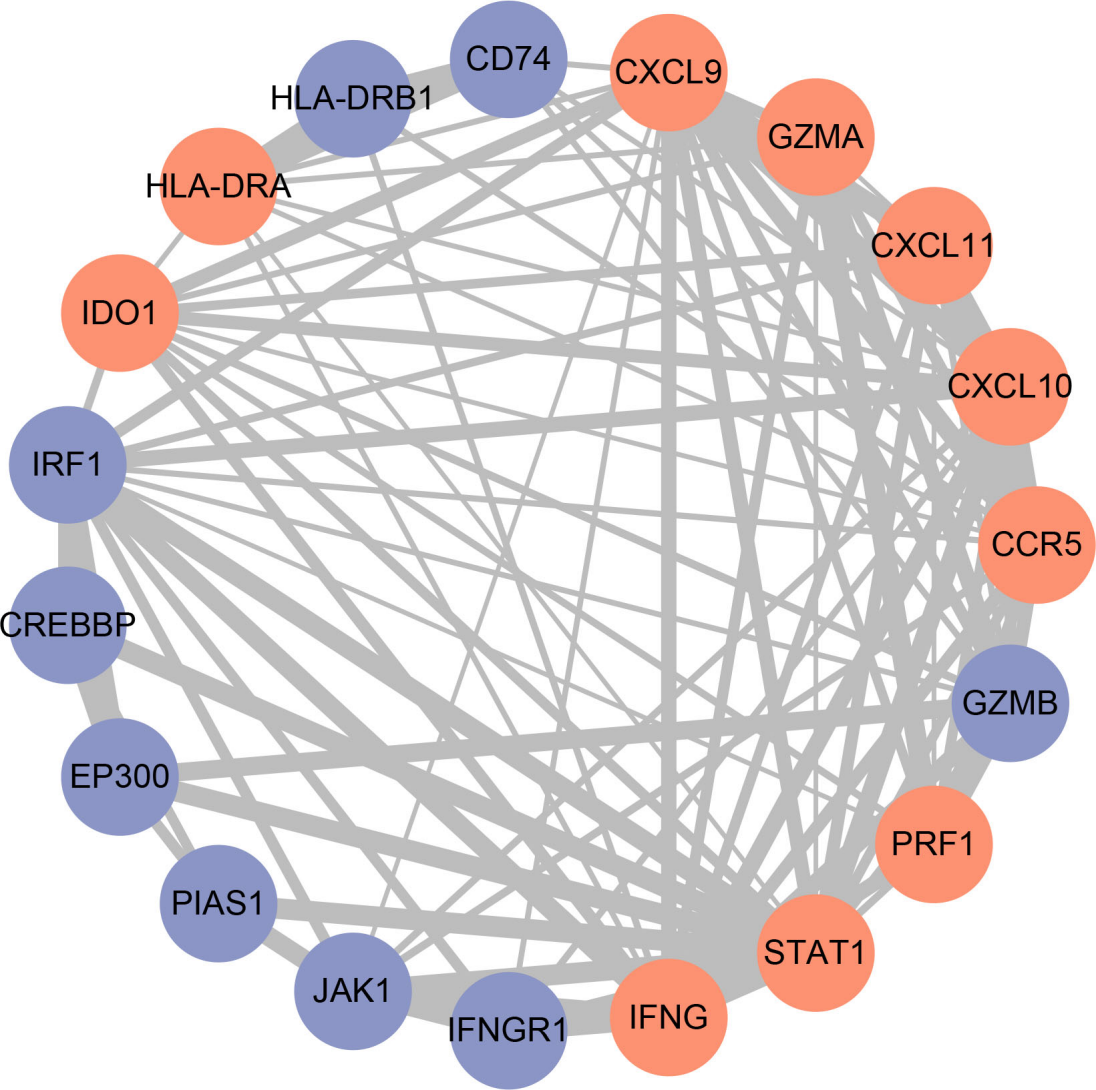
Construction of the protein-protein interaction (PPI) network. Red nodes represent IFN-γ-related genes, blue nodes represent other genes. The thickness of the lines indicates the strength of the evidence for the interaction.

**Figure 7:**
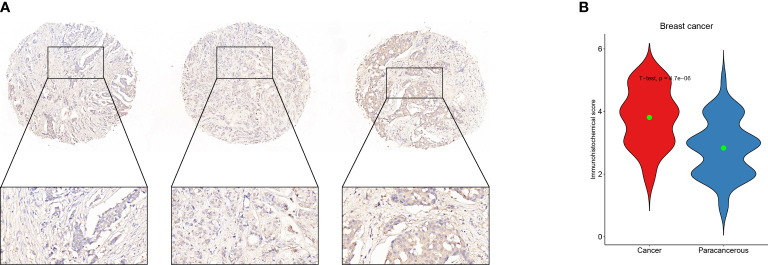
Validation of STAT1 expression in breast malignant tumors and adjacent tissues using immunohistochemistry. **(A)** Example of STAT1 expression in breast malignant tumor detected by immunohistochemistry. **(B)** Statistical analysis of STAT1 expression using Student’s t-test to represent the mean values.

In order to get the hub gene, the IFN- genes were imported into the Cytoscape program, and then a PPI network diagram was created. The proteins are denoted by the nodes, whereas the strength of the association between these proteins is denoted by the links. As can be seen, there are a total of 19 nodes in the PPI network, as well as 92 connections. The STAT1 gene is deemed to be the hub gene since it has the greatest degree of association (**Figure 6**). By means of IHC, we compared the expression of STAT1 in breast malignancies and paracancerous tissues and found that STAT1 was considerably overexpressed in the malignant breast tissues (**Figure 7**, P=4.7e-6), which was in line with the findings of our investigation.

### Single-cell functional analysis of IFN-γ

**Figure 8:**
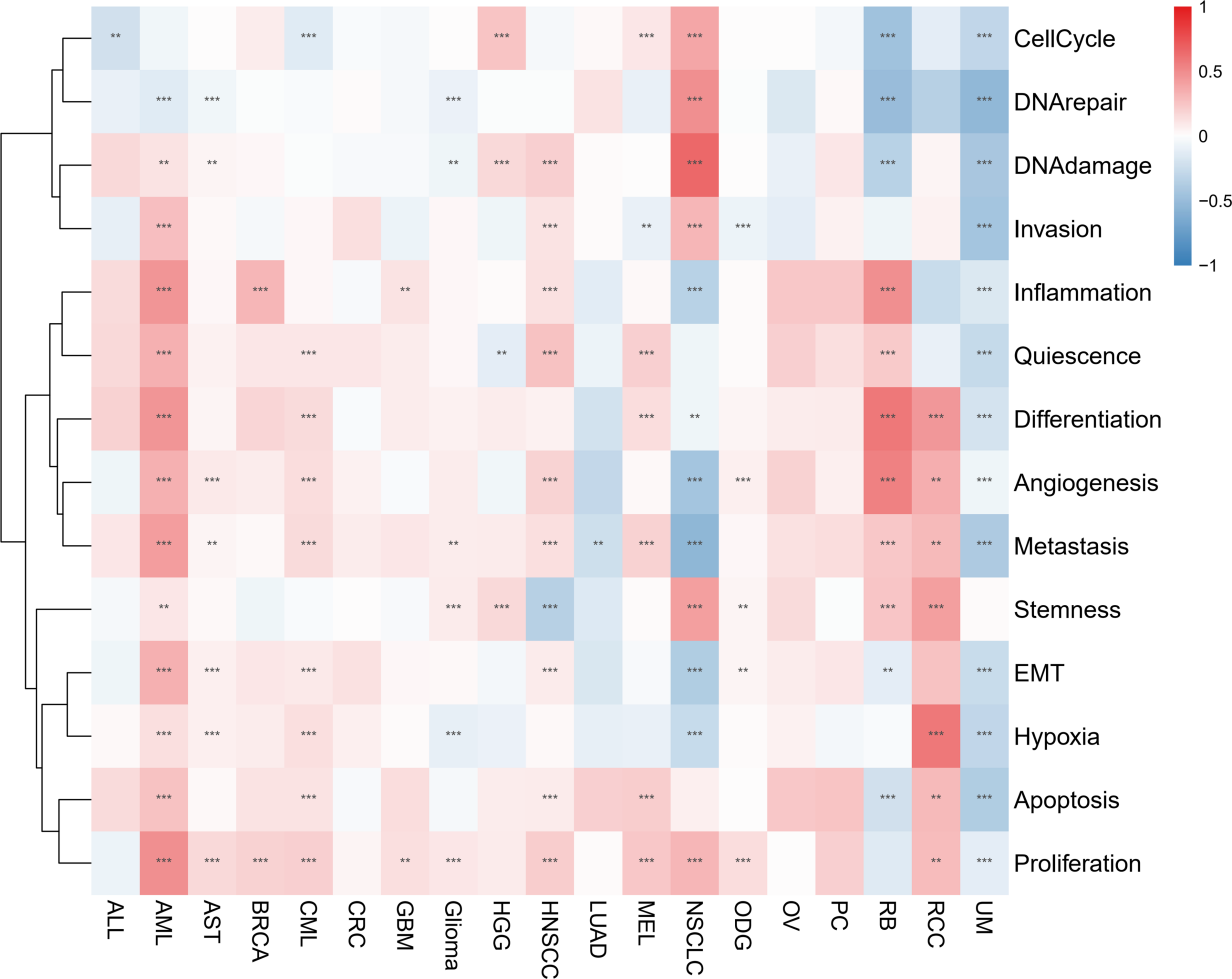
Associations between IFN-γ levels and 14 different functional states in various malignancies. Red and blue represent positive and negative associations, respectively. ** represents P<0.01, *** represents P<0.001.

Through examining the CancerSEA dataset, we compared the IFN-γ score to 14 different functional statuses of cancers. In AML, the IFN-γ score was positively correlated with inflammation, invasion, quiescence, differentiation, angiogenesis, metastasis, EMT, and other functions, but negatively correlated with 13 functions in UM. In 11 types of tumors, there was a favorable correlation between the IFN-γ score and proliferation (**Figure 8**). Combined with the information on drug responsiveness from The Cancer Therapeutics Response Portal database and information on gene expression profiles of tumor cell lines, we found that twenty of thirty drugs’ sensitivities were shown to be positively linked to STAT1. IFN-γ-related genes may serve as a target for research into these medications and targeted therapy for cancer (**Supplementary Table 2**).

### The purity of tumors correlates with levels of IFN-γ

**Figure 9:**
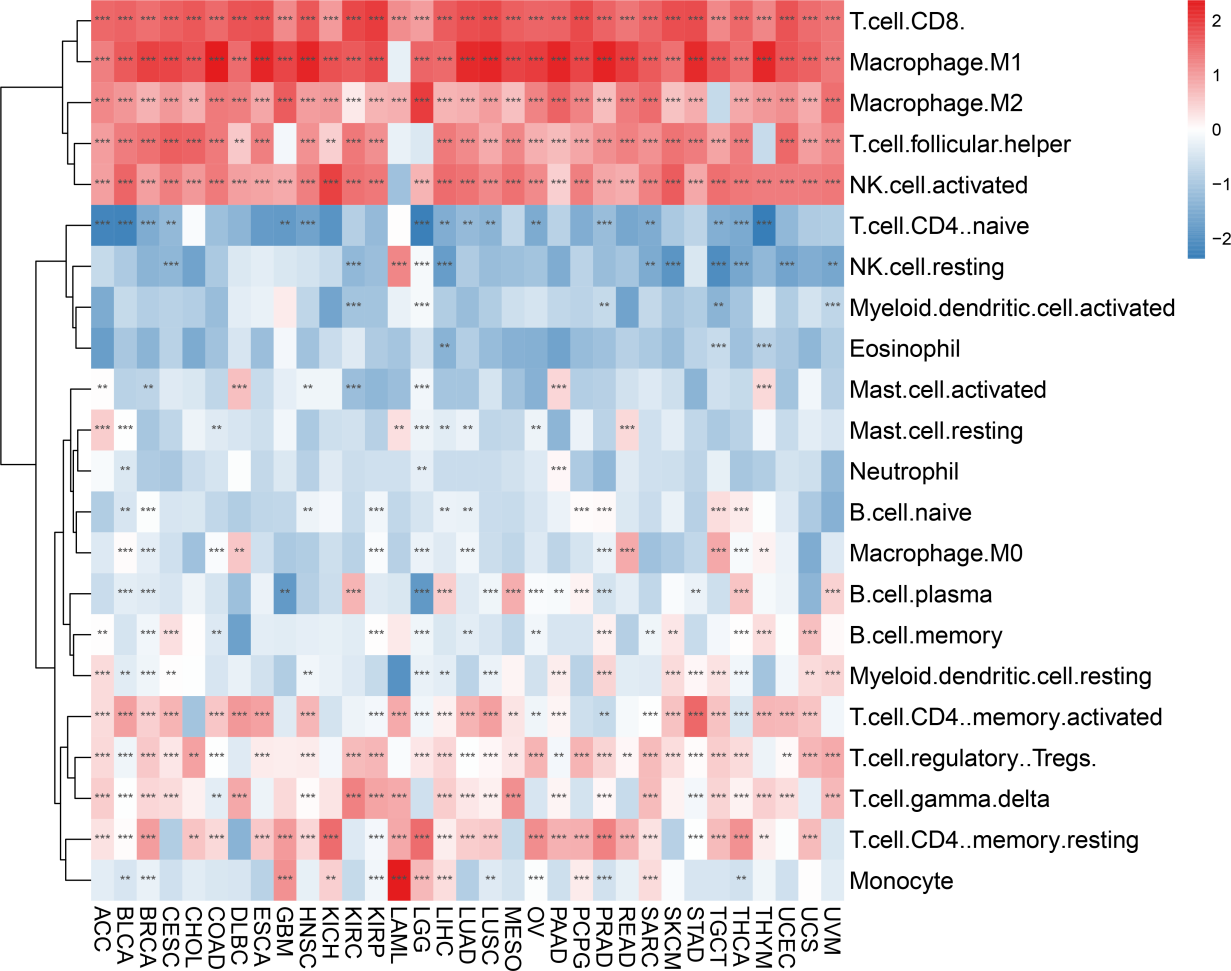
Correlation between IFN-γ scores and tumor-infiltrating immune cells. There is a correlation between IFN-γ scores and tumor-infiltrating immune cells in 33 different tumors. Red and blue represent positive and negative correlations, respectively. **p < 0.01, ***p < 0.001.

**Figure 10:**
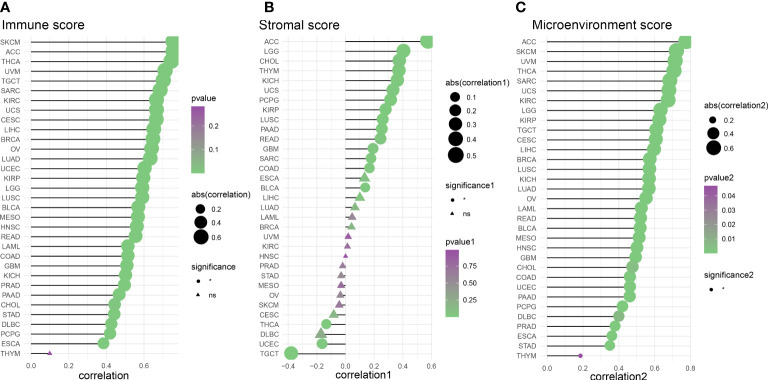
Analysis of the correlation between IFN-γ scores and tumor purity. **(A)** Tumor microenvironment score analysis based on the correlation between interferon-gamma levels and immune cell infiltration. **(B)** Tumor immune score analysis based on the correlation between IFN-γ levels and tumor microenvironment scores. **(C)** Analysis of the relationship between IFN-γ and tumor stromal scores (all P<0.05).

We evaluated 33 different types of tumors for determining associations between IFN-γ score and discovered a positive link between IFN-γ and M1 and M2 Macrophages, T cells follicular helper cells, activated NK cells, and CD8 T cells in most cancers. Also, the IFN-γ score showed an inverse association with T cell CD4 naïve and NK cell resting. (**Figure 9**). In the analysis of tumor immune score, the IFN-γ score was found to have a positive correlation with the degree of immune cell infiltration in most of the 33 tumors studied (P < 0. 05, **Figure 10A**, **Supplementary Figure 7**). For PCPG, LUSC, PAAD, SARC, READ, KIRP, COAD, GBM, UCS, KICH, THYM, CHOL, LGG, and ACC, IFN-γ score was positively linked to stromal cell score (**Figure 10B**). Additionally, the IFN-γ score had a positive link to the TME score of ACC, SKCM, UVM, THCA, SARC, UCS, KIRC, LGG, KIRP, TGCT, CESC, LIHC, BRCA, LUSC, KICH, LUAD, OV, LAML, READ, BLCA, MESO, HNSC, GBM, CHOL, COAD, UCEC, PAAD, PCPG, DLBC, PRAD, ESCA, STAD, and THYM (All P < 0. 05, **Figure 10C**). The above results indicate that IFN-γ is closely related to the immune status of tumors.

### Association of IFN-γ score with genes involved in immunity

**Figure 11:**
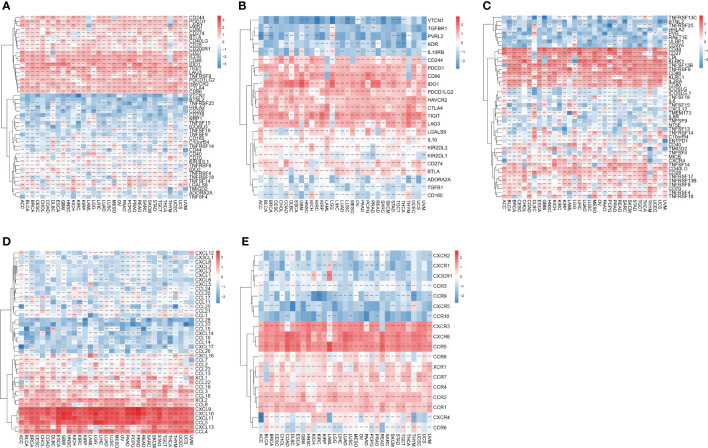
Relationship between IFN-γ levels and immune-related genes. **(A)** Association between immune checkpoint status and IFN-γ levels in human malignancies. **(B)** Association between immune inhibitory genes and interferon-gamma scores in human cancers. **(C)** Association between IFN-γ scores and expression of immune activation genes in human tumors. **(D)** Association between chemical factors and IFN-γ levels in human malignancies. **(E)** Association between IFN-γ scores and expression of chemical factor receptors in human tumors. **p < 0.01, ***p < 0.001.

To investigate the involvement of IFN-γ in immune modulation, we investigated whether or not there was a correlation between the IFN-γ score and the presence of ICGs in human malignancies. The results of the association between IFN-γ score and ICG indicated that the IFN-γ scores of virtually all of the different cancers that were investigated had a positive association with the expression of TIGIT, IDO1, ICOS, CD86, CTLA4, HAVCR2, PDCD1LG2, and CD48 (**Figure 11A**). Further, we analyzed 23 immunosuppression genes for their association with the IFN-γ score. The expression levels of LAG3, TIGIT, CD96, IDO1, PDCD1, HAVCR2, CTLA4, PDCD1LG2, CD244, and CD244 were positively linked to IFN-γ scores in almost all evaluated cancer types. In 13 tumors, the IFN-γ score was inversely linked to VTCN1 expression, whereas in 16 tumors, it was inversely linked to KDR expression (**Figure 11B**). In 32 different cancers, the IFN-γ score was strongly linked to CD86, CD48, KLRK1, LTA, CD27, TNFSF13B, TNFRSF9, CD40LG, KLRC1, IL2RA, and CD80, out of a total of 46 immune activation genes in pan-cancer (All P < 0. 05, **Figure 11C**). Simultaneously, we explored the link between IFN-γ score and chemokines. The findings demonstrated a positive link between IFN- score and the expression of CCL5, CXCL11, CXCL9, CXCL10, CCL4, CXCL13, CCL3, CCL8, and CCL2 chemokine genes (**Figure 11D**). Positive correlations were observed between IFN-γ score and the chemokine receptor genes CXCR6, CCR1, CCR5, CCR2, and CXCR3, and negative correlations with CXCR2, CCR1, CCR9, and CCR10 (**Figure 11E**) Our results are consistent with previous studies which indicate that immune checkpoint genes (ICGs) perform a remarkable function in regulating the infiltration of immune cells as well as immunotherapy (35).

### Correlation between IFN-γ score and immunotherapy response markers

**Figure 12:**
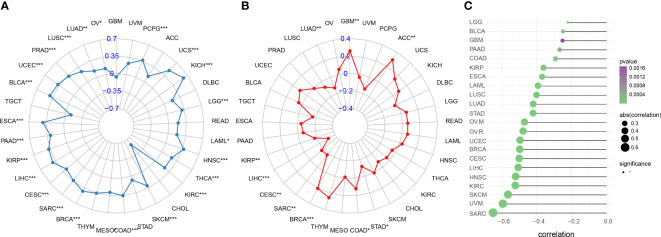
Immune therapy response indicators associated with IFN-γ in human malignancies. **(A)** Association between IFN-γ levels and tumor mutation burden in various cancers. **(B)** Association between microsatellite instability and interferon-gamma levels in cancers. **(C)** Association between IFN-γ scores and tumor immune dysfunction and exclusion scores. *p < 0.05, **p < 0.01, ***p < 0.001.

Immunotherapy outcomes may be predicted by monitoring the tumor’s immune escape process. For most cancers, we observed a favorable correlation between IFN-γ score and TMB. The TMB was strongly linked to IFN-γ score for PCPG, OV, LGG, LUSC, PRAD, THCA, LAML, COAD, ESCA, SARC, LIHC, CESC, BRCA, KIRP, MESO, PAAD, SKCM, BLCA, KIRC, UCEC, HNSC, KICH, LUAD, and UCS (All P<0.05, **Figure 12A**). Furthermore, we investigated whether or not the IFN-γ score was related to MSI. A higher IFN-γ score was associated with a lower prevalence of MSI in GBM, ACC, and BRCA (All P<0.05, **Figure 12B**). TIDE scores, like TMB and PD-L1, are one of the popular markers used to predict the effect of ICB treatment. Low ICB response was recorded in patients with elevated TIDE scores because of the increased risk of tumor immune evasion in these patients. In an examination of 22 cancers, the correlation between TIDE and IFN-γ scores was inverse in all 22 tumors. Evidence like this points to a link between IFN-γ expression and ICB response (**Figure 12C**, **Supplementary Figure 8**) This provides a basis for further investigation of whether the genes associated with IFN-γ can be used as potential markers of ICB therapy and modulators of immune checkpoint inhibition therapy.

### Single-cell transcriptome analysis of IFN-γ in KIRC tumor microenvironment

**Figure 13:**
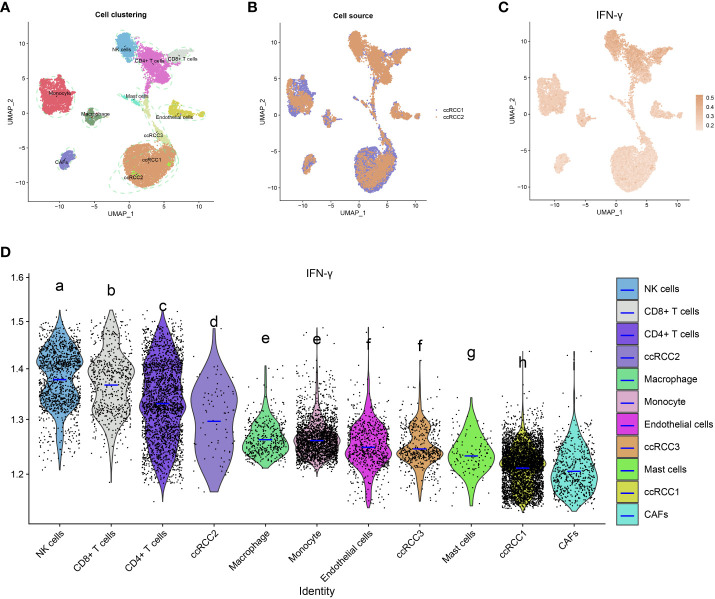
IFN-γ in the tumor microenvironment of KIRC. **(A)** t-distributed stochastic neighbor embedding (tSEN) plots showing 11 different cell types in KIRC samples. **(B)** tSEN plots of two KIRC samples. **(C)** IFN-γ scores of different cell types displayed on the tSEN plots. **(D)** Analysis of IFN-γ levels in different cell types in the tumor microenvironment of KIRC. The violin plots show the median of the IFN-γ scores. The letters at the top indicate whether there is a statistically significant difference between two cells. Different letters represent different levels of statistical significance.

Quality control was performed by Seurat, and then 13124 high-quality single-cell transcriptomic data were selected for further analyses. The aforementioned cells may be classified into 11 groups, as determined by a tSNE-based cell clustering analyses: monocyte1, monocyte2, KIRC1, KIRC2, KIRC3, macrophages, mast cells, endothelial cells, NK cells, CD4+T cells, and CD8+T cells (**Figure 13A**). Variations in marker gene expression were highly significant across cell types (**Supplementary Figure 9**). We also discovered that cancerous cells from two independent KIRC samples cluster together into the same cluster (KIRC3) as well as many other distinct clusters (KIRC1 and KIRC2). The above findings demonstrate the heterogeneity within the KIRC cell type (**Figure 13B**). To assess the variations in IFN-γ scores across cell types, we conducted ssGSEA to summarize the IFN- scores of cells in the KIRC TME. Notably, we found significant differences in IFN-γ scores among different cells (**Figure 13C**). KIRC cells had the least IFN-γ score, suggesting that this marker more accurately represented the TME than the tumor itself. IFN-γ scores varied significantly across KIRC cell subsets, suggesting that IFN-γ expression is a potential KIRC cell characteristic (**Figure 13D**). Based on this analysis, it appears that IFN-γ is significantly different among different cells of KIRC TME. As a consequence, targeting IFN-γ could represent a substantial step forward in TME regulation.

## Discussion

IFN-γ is a protein that is produced by two polypeptide chains that are linked together in an antiparallel manner and are encoded by the IFNG gene (36). During the innate immune response, natural killer (NK) and natural killer T (NKT) cells are the primary cells involved in regulating IFN-γ synthesis. On the other hand, during the adaptive immunological response, CD8+ and CD4+ T cells are the primary paracrine producers of IFN-γ (37). IFN-γ maintains a steady level of coordination between pro-tumor and anti-tumor immune function in the tumor microenvironment (TME) (38, 39). IFN-γ is implicated in the eradication of cancer by preventing the growth of new blood vessels, suppressing the proliferation of existing cells, enhancing apoptosis, stimulating adaptive immunity, and improving antigen processing and presentation (40). Research indicates that IFN-γ may selectively and dosage- dependently trigger apoptotic death of stem cell-like carcinoma cells in colon cancer patients through JAK-STAT1-IRF1 signal transmission (41). IFN-γ- deficient animals were shown to develop lung epithelial tumors and lymphomas spontaneously, providing additional evidence that IFN-γ is involved in the immunity against tumors and validating IFN-γ’s anti-cancer property (42, 43). Other immunosuppressive processes may be activated by IFN-γ because of its ability to induce the synthesis of indoleamine-2,3-dioxygenase (IDO) and immune checkpoint inhibitory molecules (44, 45). IFN-γ is important for cancer immunity and treatment. However, the relationship between IFN-γ and immunity is still the focus of the literature. Therefore, we conducted a systematic pan-cancer investigation of 10 IFN-γ-related genes using several databases. This research may provide the necessary strategy to maximize the anti-tumor effects of IFN-γ.

First, we analyzed the differential expression of IFN-γ-related genes in 33 tumors and found that these genes were upregulated in most tumors, especially in CESC, GBM, OV, SKCM, and TGCT. Additionally, tissue concentrations of IFN-γ were significantly higher in the cervical tissues of patients with cervical cancer (46). Also, evidence from human esophageal cancer samples demonstrated an increased level of IFN-γ in tumor tissue, which linked favorably to tumor growth and was in line with our findings (47). We analyzed the link between IFN-γ and survival rate to better understand its role in clinical risk stratification. According to the results of the survival study, OS, DSS, and PFI were all linked to IFN-γ overexpression. In the investigation of the prognostic implications of IFN-related gene expression, it was discovered that patients with COAD, LIHC, BRCA, SKCM, ACC, HNSC, and SARC had improved prognoses when IFN-γ expression levels were elevated (all p<0.05). In contrast, a worse prognosis was observed in individuals with LGG, UVM, KIRP, PAAD, and THYM who had elevated IFN-γ expression levels (all p<0.05). Previous studies found that IFN-γ inhibits the development of squamous cell carcinoma, which provided strong evidence for our findings (48).

We evaluated IFN-γ scores in tumor and normal samples of 33 cancers and found that most tumors had higher IFN-γ scores than normal tissue. INF-γ is a cytokine that promotes inflammation and is proven to be intimately linked to both innate and acquired immune responses (49, 50). Chronic inflammation can induce tumors, and the inflammatory microenvironment of tumors and exposure to tumor antigens trigger the infiltration of immune cells. Thus, IFN-γ scores are elevated in tumors that are closely associated with the inflammatory features of tumors. IFN-γ has antitumor effect. A previous study showed that M1 macrophages can be induced *in vitro* by IFN-γ, which can trigger a rapid pro-inflammatory response, and pathogen clearance and show anti-tumor activity (51). IFN-γ promotes migration of immune cells to TME by transcriptionally regulating the expression and secretion of CXCL9, CXCL10 and CXCL11 and their cognate receptor CXCR3 in T cells, NK cells, monocytes, DCs and cancer cells. The increase in chemotaxis of activated CTL towards TME enhances cytotoxic effects and limits tumor growth. In addition, IFN-γ can play an anti-tumor role by promoting macrophage activation, up-regulating the expression of antigen processing and presenting molecules, boosting the growth and activation of Th1 cells, facilitating the function of NK cells, and regulating the function of B cells. Therefore, IFN-γ promotes a severe inflammatory response in the tumor and shows a good prognosis. In addition, we observed large variation in IFN-γ scores across tumors, which may be related to differences in the inherent characteristics of different tumors. It has been found that IFN-γ is under transcriptional control and epigenetic control, such as chromosome access, DNA methylation and histone acetylation (52). There is variability in IFN-γ scores because the aforementioned functional activity varies among tumors.

To a large extent, TMB determines the immune response of cancer patients to treatment with immune checkpoint inhibitors (ICIs), either, anti-cytotoxic T cell-associated antigen 4 (CTLA-4) or anti-programmed cell death 1 (PD-1) (53–55). Researchers discovered that TMB has a significant role in tumor immunotherapy success. TMB is a good indicator of the effectiveness of immune checkpoint inhibitor (ICI), with larger values indicating better efficacy (56, 57). Additionally, we evaluated the link between IFN-γ scores and tumor immunity and discovered that, IFN-γ scores were positively correlated with TMB in most tumors. Numerous research reports have demonstrated that tumors with increased TMB are more likely to respond favorably to cancer immunotherapy (58). For instance, among non-small-cell lung cancer(NSCLC) patients treated with anti-PD-1/L1, patients with high TMB had longer associated PFS than those with low TMB (59). We found that IFN-γ scores were inversely linked to TIDE scores in most tumors. The lower the TIDE, the lower the possibility of immune escape, the higher the response rate to ICB treatment, and the better the clinical outcome of immunotherapy (24). Therefore, it can be inferred that IFN-γ is an indicator of a good response to tumor immunotherapy.

We examined the differential methylation of IFN-γ-related genes in 13 distinct cancers. We found variation in methylation patterns across tumors, and this phenomenon is similar to the findings of Saghafinia et al. (60) That may be caused by intrinsic differences in different tumors. In 13 different cancers, we discovered a statistically significant inverse association between the expression of most IFN-γ-related genes and methylation. Our findings were supported by data showing that the transcriptional activity of the entire IFN-γ promoter vector may be suppressed by its methylation (61, 62). Also, DNA hypermethylation in the IFN-γ promoter region was found in a vast number of cervical cancer samples, which may be linked to carcinogenesis in this disease. This suggests that methylation-mediated IFN-γ gene silencing contributes significantly to the mechanism of cervical carcinogenesis (63). However, we observed a significant positive correlation between the methylation level of IDO1 and RNA expression (64). Sailer and others similarly observed a significant positive correlation between IDO1 methylation levels and RNA expression in HNSCC (64). The reason for this phenomenon is mainly that methylation of IDO1 occurs mostly within the gene rather than the CPG island.

However, this research has several drawbacks. The current research only offers preliminary data on the association of IFN-γwith a wide range of tumor progression, and additional experimental work is required to clarify the specific molecular roles and processes of IFN-γ in carcinogenesis. Confirmation of our conclusions requires more research at the molecular and cellular levels. Meanwhile, the specific mechanisms involved in the regulation of immunity by IFN-γ remain unclear. In addition, there is a lack of specific and complete cases from which to draw inferences about the effectiveness of various medications in suppressing tumor development. Since IFN-γ processing can enhance tumor immunity by increasing T-cell and macrophage activity (65, 66), tumor cells resistant to IFN-γ may not necessarily be caused by their own drug resistance, but may be caused by the tumor promoting immune escape or creating an immunosuppressive microenvironment. We will further explore the mechanism of IFN-γ resistance in a subsequent study. Finally, the control group in this study included non-cancer samples sourced from the GTEx database. However, GTEx consisted of tissues sampled from abruptly deceased individuals, which may impact the expression of immune genes and therefore influence the research findings.

## Conclusion

This paper presents a pan-cancer analysis of IFN-γ in different tumors. Additionally, we presented novel concepts and perspectives for future tumor immunotherapy, highlighting the potential utility and application direction of IFN-γ for further tumor immunotherapy.

## Data availability statement

The original contributions presented in the study are included in the article/**Supplementary Materials**, further inquiries can be directed to the corresponding author/s.

## Ethics statement

This study was approved by The Affiliated Cancer Hospital of Guangxi Medical University’s Ethics and Anthropology Committee. Written informed consent from obtained from the patients/participants.

## Author contributions

HR, YZ, TQ, YJZ, XW, YQ, and WL conceived and designed the experiments; HR analyzed the data; YZ, TQ, YJZ, XW, YQ, and WL helped with reagents/materials/analysis tools; and XW, YQ, WL, YZ, TQ, and YJZ contributed to the writing of the manuscript. All authors reviewed the manuscript. All authors contributed to the article and approved the submitted version.

## Supplementary material

Supplementary Figure 1 Flow chart of this study.

Supplementary Figure 2 Stage-specific IFN-γ score for pan-cancer.

Supplementary Figure 3 Overall survival as determined by the Kaplan-Meier method for cancer patients with high and low IFN-γ expression levels.

Supplementary Figure 4 Kaplan-Meier survival curves of disease-specific survival in patients with cancer with high versus low expressions of the IFN-γ.

Supplementary Figure 5 Kaplan-Meier plots showing progression-free interval (PFI) survival rates in cancer patients having high and low levels of IFN-γ. Patients with an HR>1 have a dismal prognosis.

Supplementary Figure 6
**(A–E)** Comparison of immune activation genes, immune suppression genes, immune checkpoints, chemokines, chemokine receptor genes scores in cancer versus non-cancer. **(F)** Comparison of random sets of immune genes scores in cancer versus non-cancer. Red indicates that the score was significantly increased in tumors and FDR<0.05, blue indicates that the score was significantly decreased in tumors and FDR<0.05, and white indicates FDR>0.05.

Supplementary Figure 7 Link between immune score and IFN-γ score.

Supplementary Figure 8 There is a correlation between IFN-γ scores and markers of responsiveness to immunotherapy.

Supplementary Figure 9 Different cells in the kidney renal clear cell carcinoma tumor microenvironment express the IFN-γ marker genes.
