# Supplementary figures and images for: Pan-cancer analysis of IFN-γ with possible immunotherapeutic significance: a verification of single-cell sequencing and bulk omics research
Source: Front Immunol. 2023 Aug 14;14:1202150. doi: 10.3389/fimmu.2023.1202150 (PMC10461559; doi:10.3389/fimmu.2023.1202150)

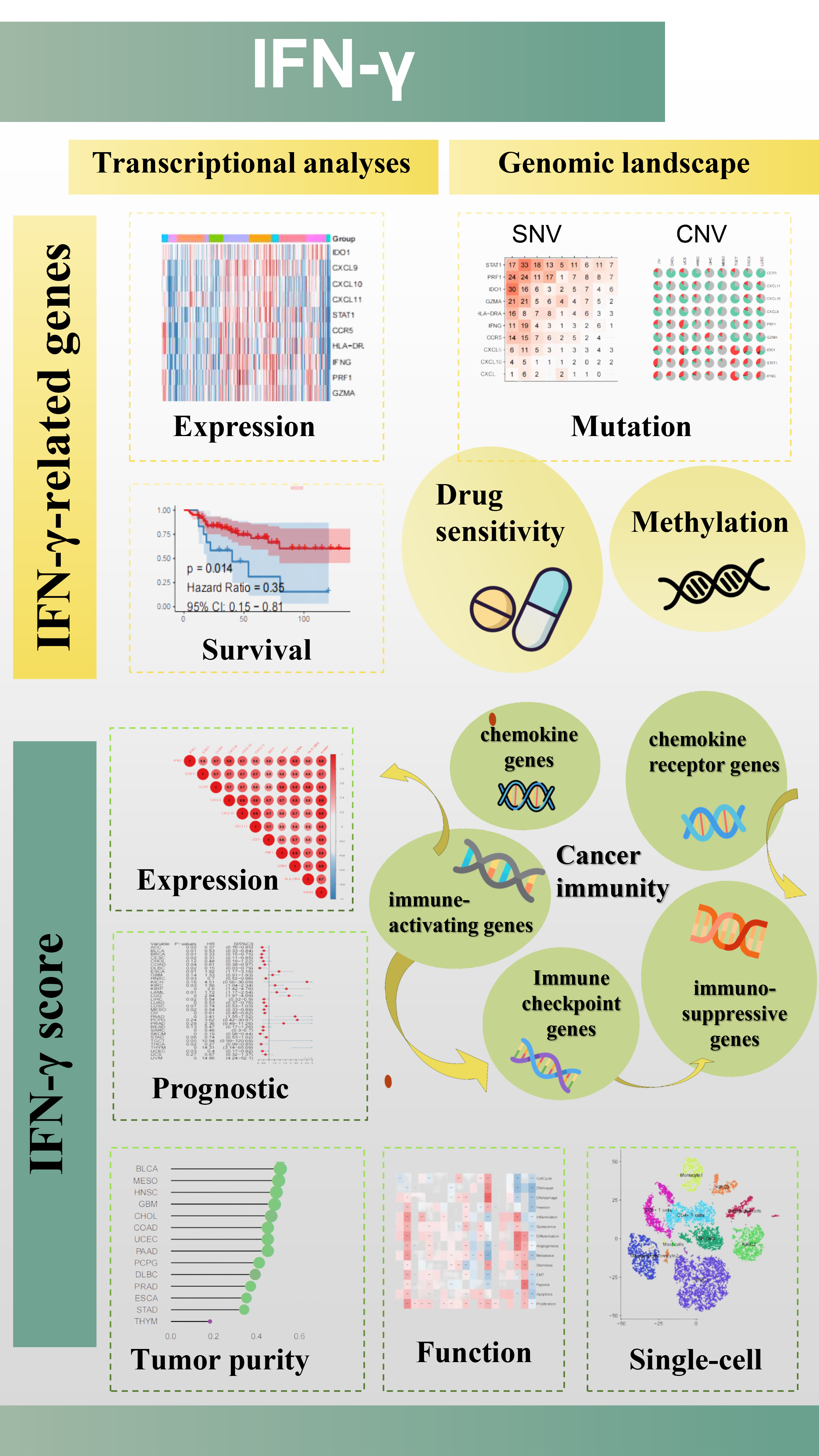

Supplement: Supplementary Figure 1 — Flow chart of this study. [file Image_1.tif]

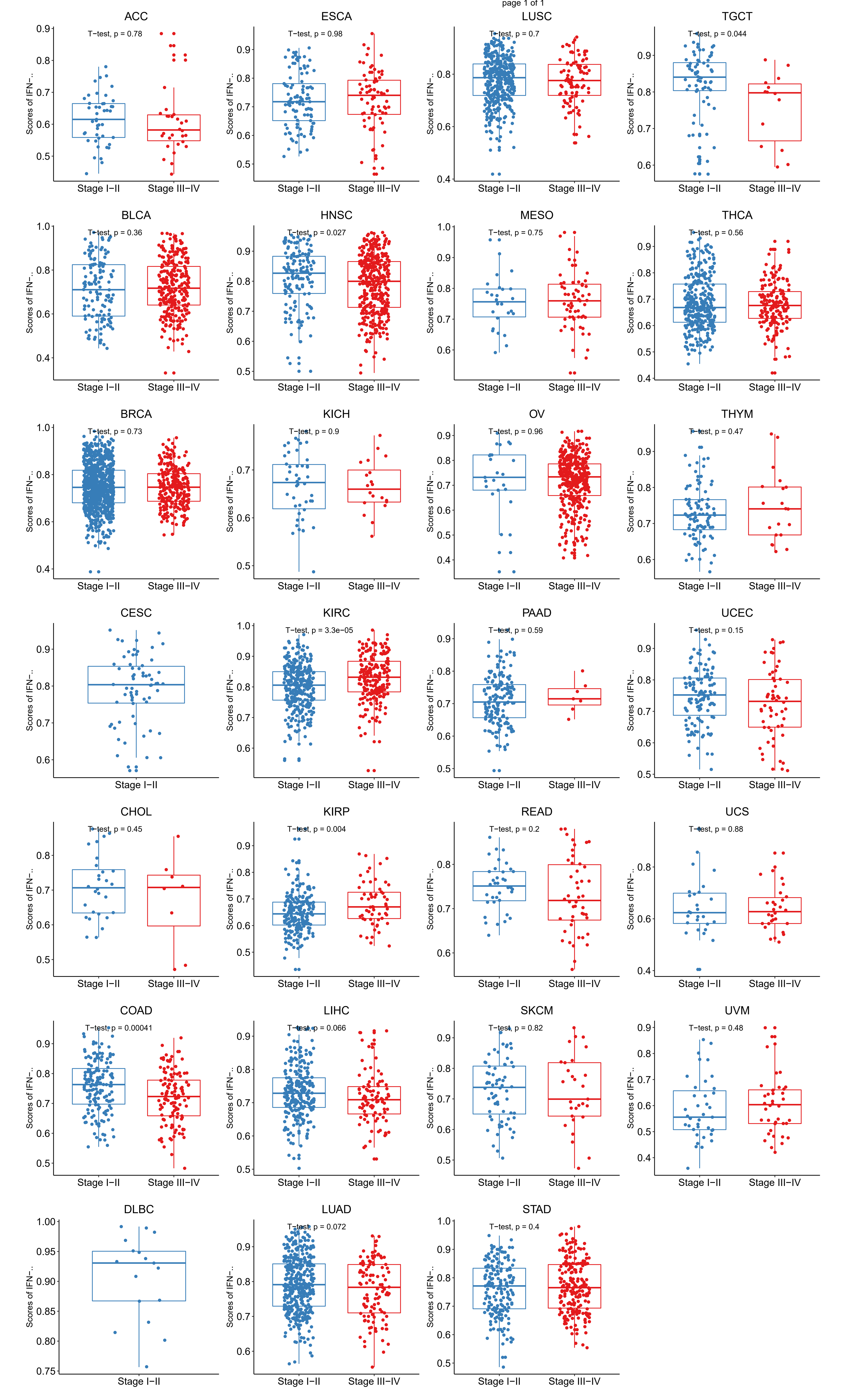

Supplement: Supplementary Figure 2 — Stage-specific IFN-γ score for pan-cancer. [file Image_2.tif]

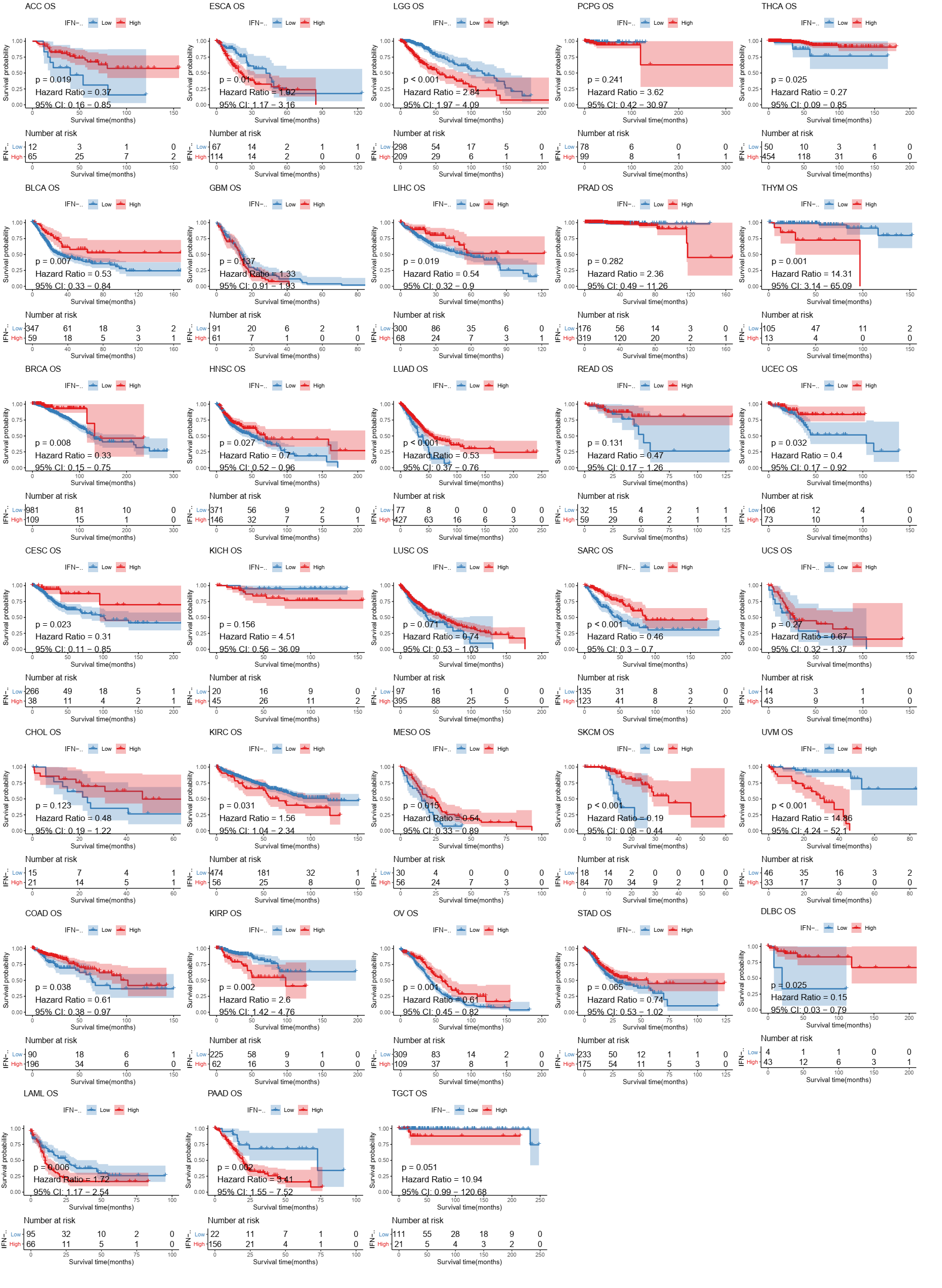

Supplement: Supplementary Figure 3 — Overall survival as determined by the Kaplan-Meier method for cancer patients with high and low IFN-γ expression levels. [file Image_3.tif]

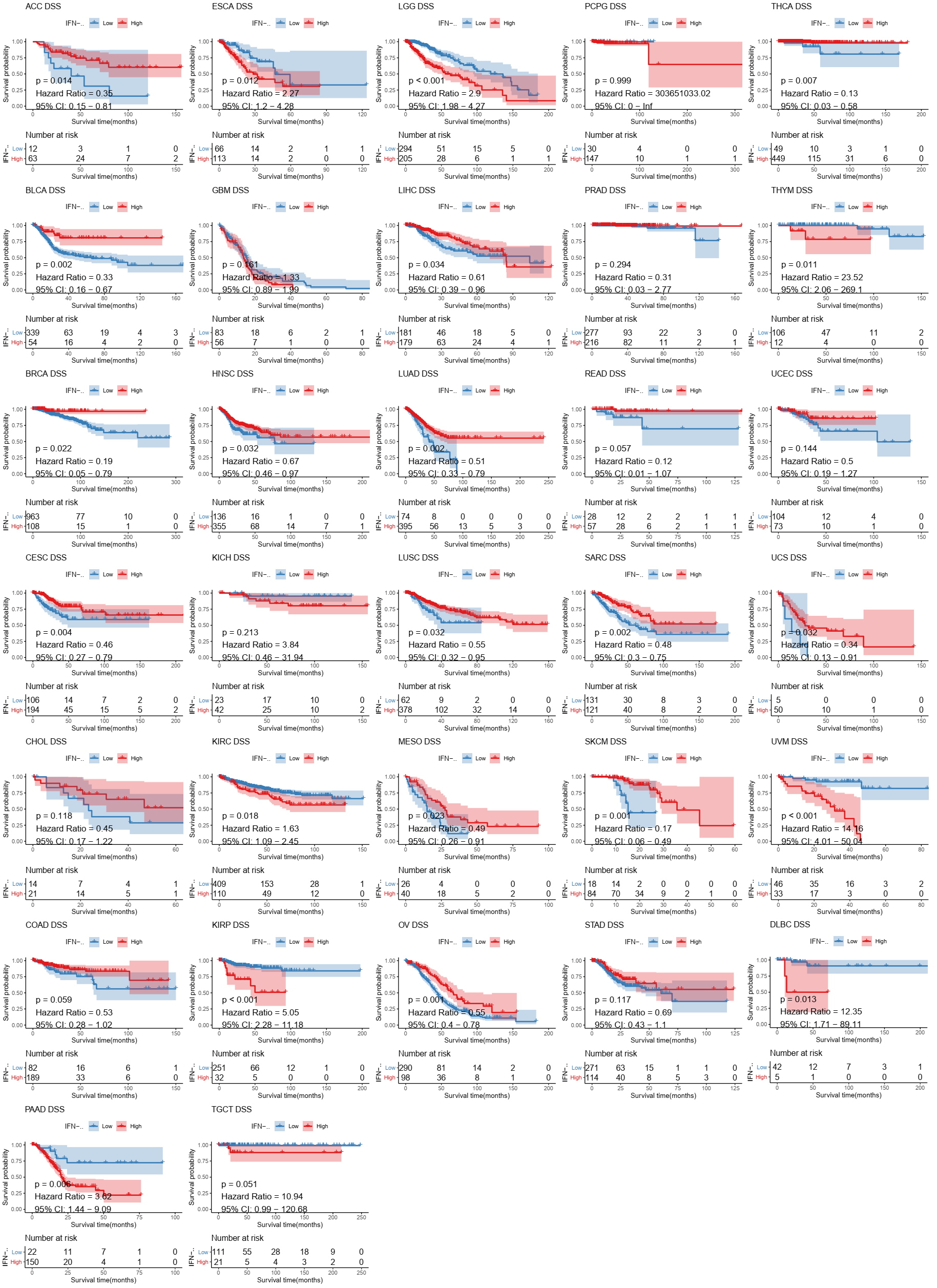

Supplement: Supplementary Figure 4 — Kaplan-Meier survival curves of disease-specific survival in patients with cancer with high versus low expressions of the IFN-γ. [file Image_4.tif]

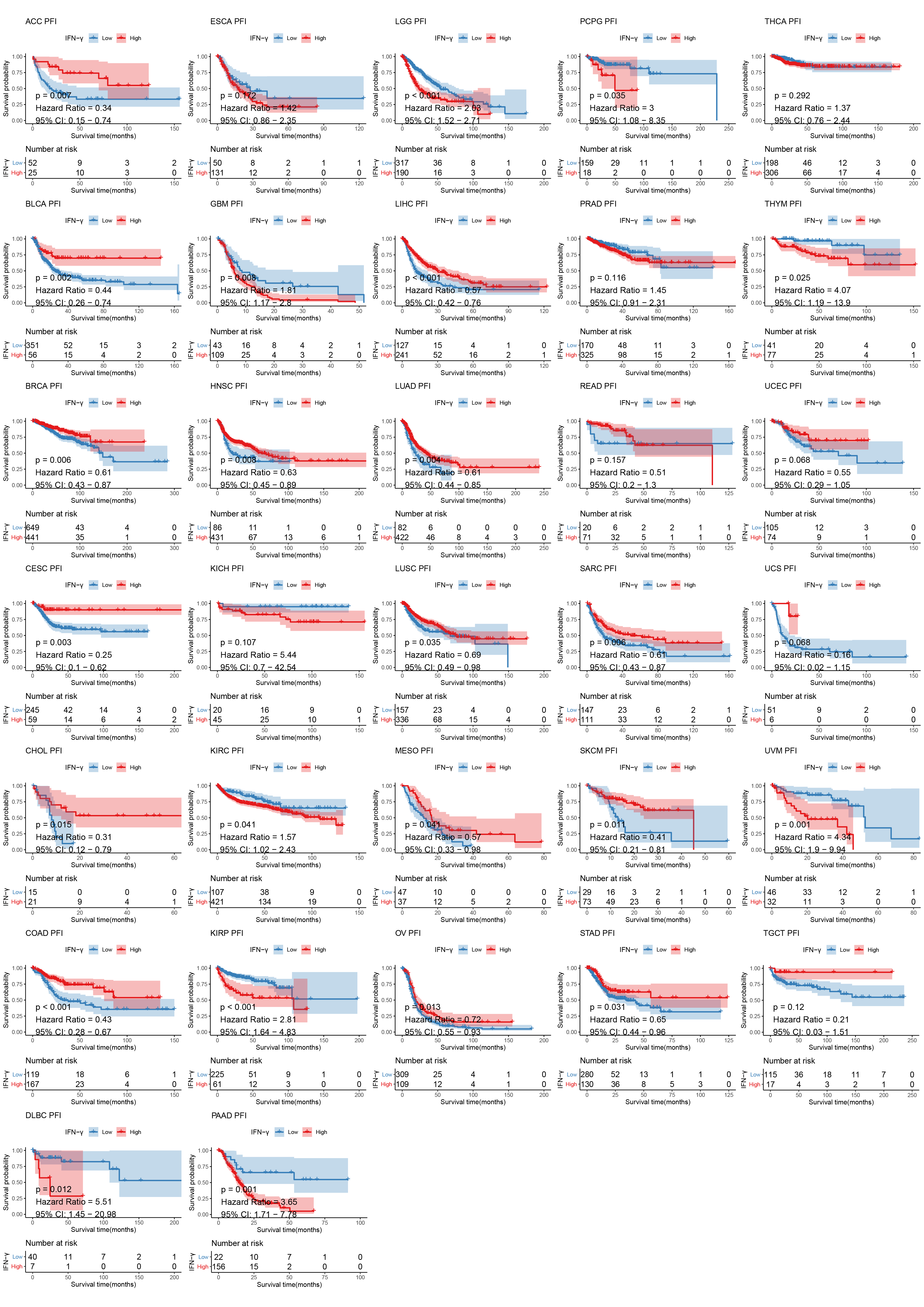

Supplement: Supplementary Figure 5 — Kaplan-Meier plots showing progression-free interval (PFI) survival rates in cancer patients having high and low levels of IFN-γ. Patients with an HR>1 have a dismal prognosis. [file Image_5.tif]

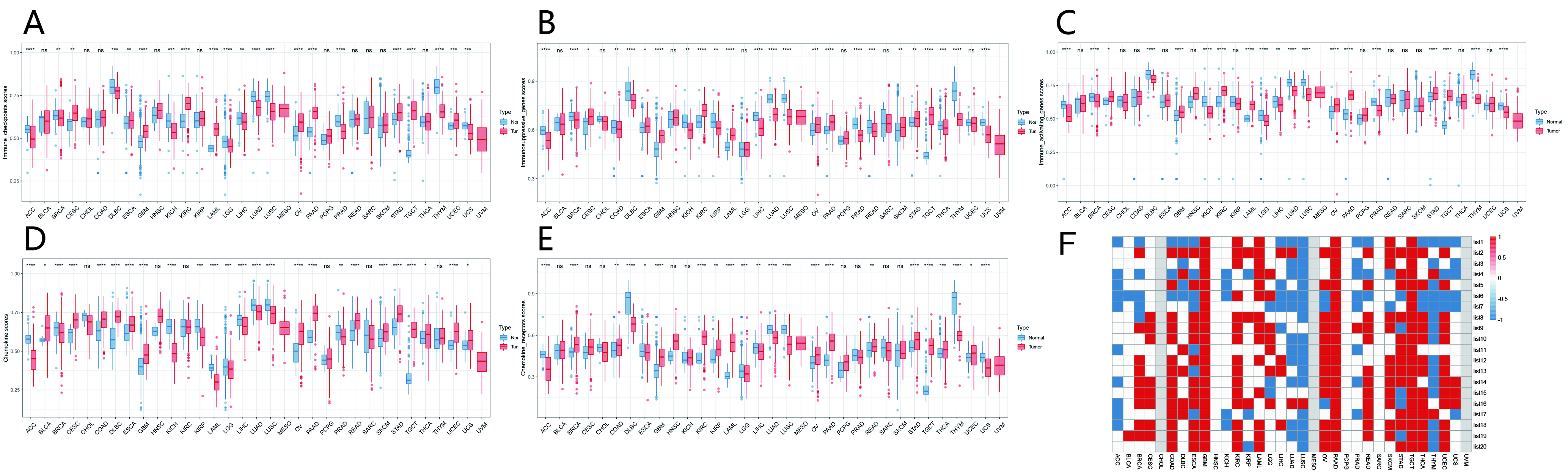

Supplement: Supplementary Figure 6 — (A–E) Comparison of immune activation genes, immune suppression genes, immune checkpoints, chemokines, chemokine receptor genes scores in cancer versus non-cancer. (F) Comparison of random sets of immune genes scores in cancer versus non-cancer. Red indicates that the score was significantly increased in tumors and FDR<0.05, blue indicates that the score was significantly decreased in tumors and FDR<0.05, and white indicates FDR>0.05. [file Image_6.tif]

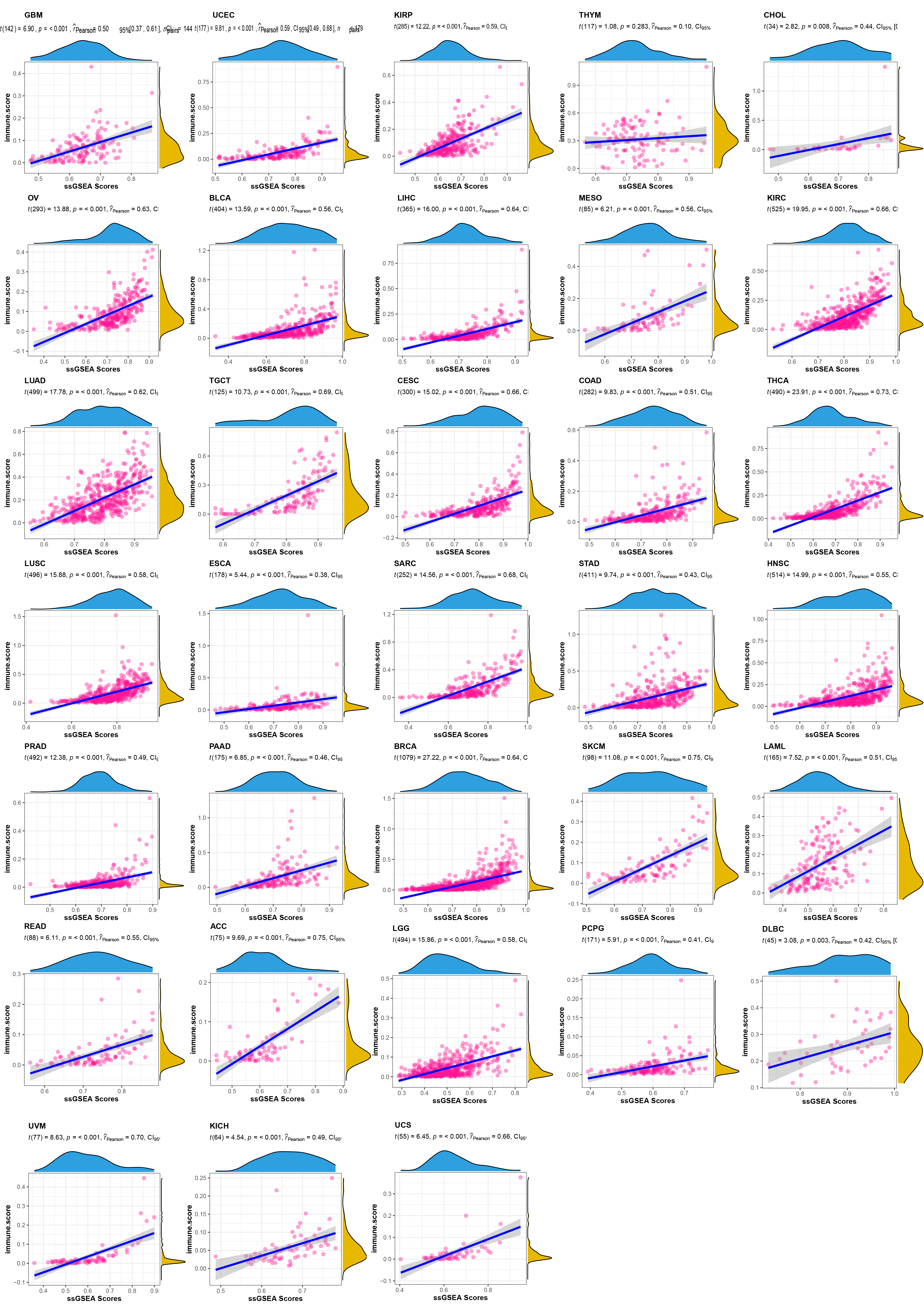

Supplement: Supplementary Figure 7 — Link between immune score and IFN-γ score. [file Image_7.tif]

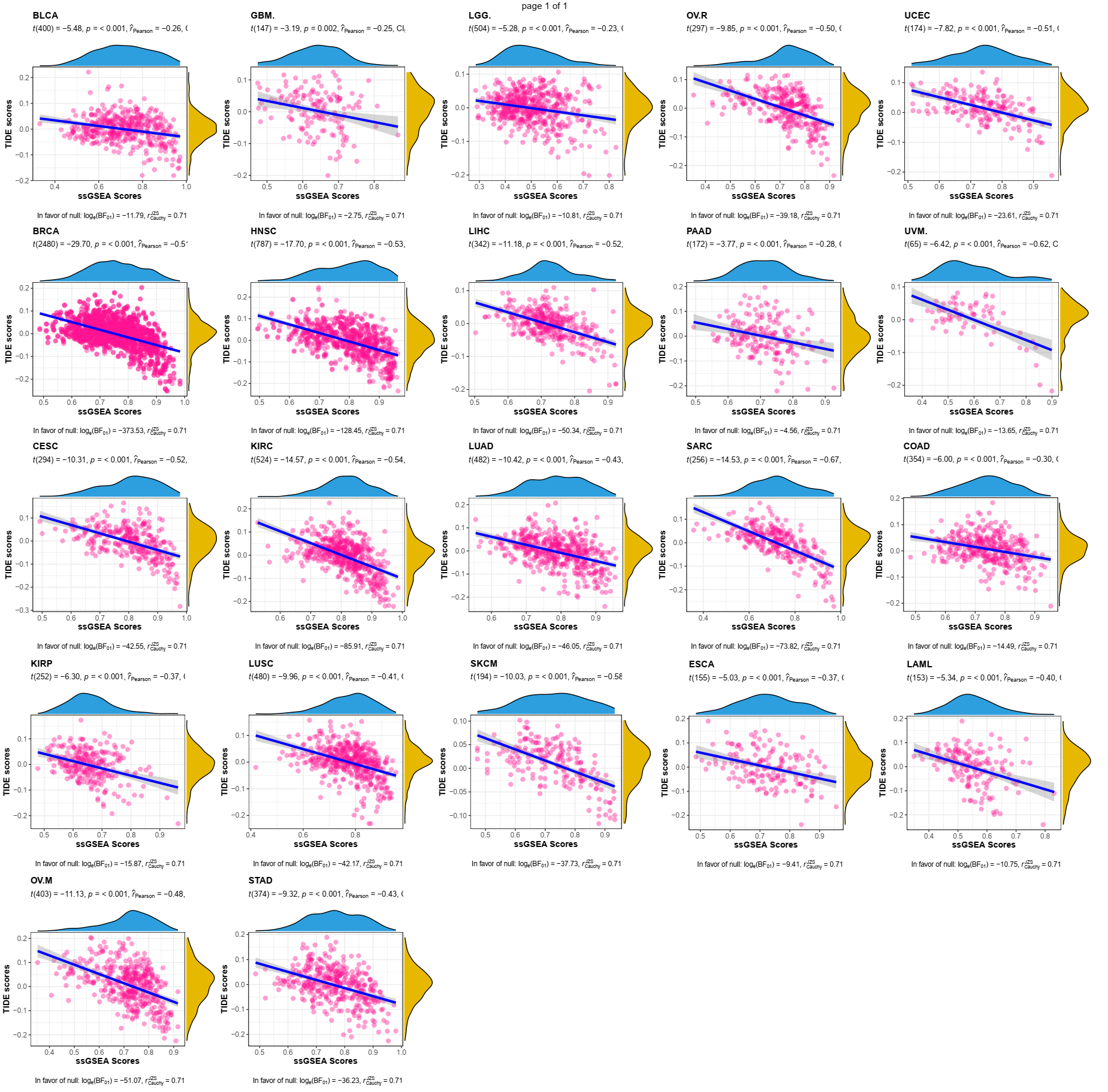

Supplement: Supplementary Figure 8 — There is a correlation between IFN-γ scores and markers of responsiveness to immunotherapy. [file Image_8.tif]

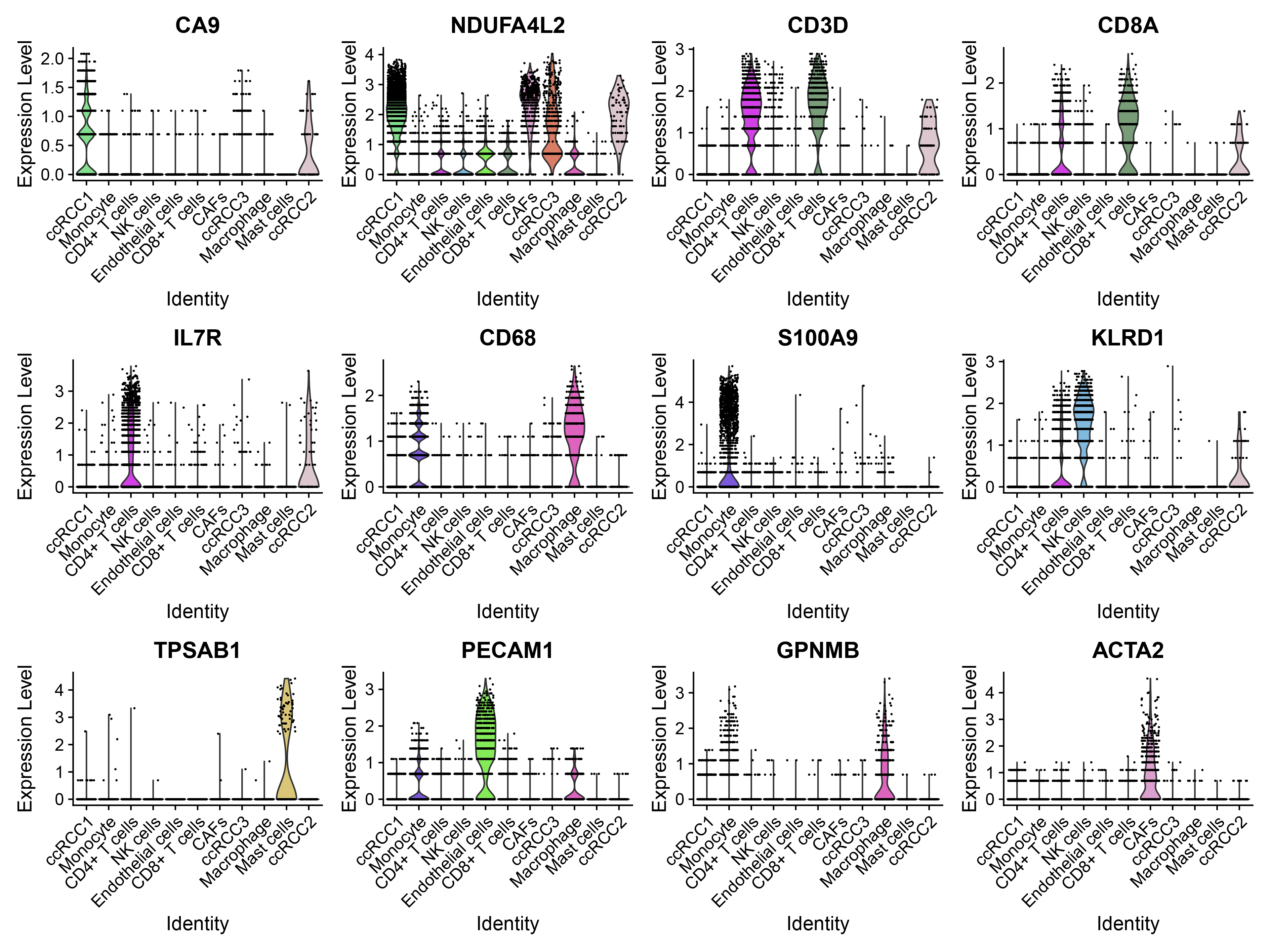

Supplement: Supplementary Figure 9 — Different cells in the kidney renal clear cell carcinoma tumor microenvironment express the IFN-γ marker genes. [file Image_9.tif]
