# Supplementary material for: Pan-cancer analysis of IFN-γ with possible immunotherapeutic significance: a verification of single-cell sequencing and bulk omics research
Source: Front Immunol. 2023 Aug 14;14:1202150. doi: 10.3389/fimmu.2023.1202150 (PMC10461559; doi:10.3389/fimmu.2023.1202150)
Supplement: Supplementary file 11 [file Table_2.docx]

Supplementary Table 2: Quantitative information on drug sensitivity

| Symbol drug cor fdr |
| --- |
| \| CCR5 \| (5Z)-7-Oxozeaenol \| -0.06448 \| 0.111506 \| \| --- \| --- \| --- \| --- \| \| CCR5 \| 17-AAG \| 0.065254 \| 0.084818 \| \| CCR5 \| 5-Fluorouracil \| -0.28942 \| 1.59E-17 \| \| CCR5 \| 681640 \| 0.090805 \| 0.102747 \| \| CCR5 \| A-443654 \| -0.03122 \| 0.886306 \| \| CCR5 \| A-770041 \| 0.070836 \| 0.375566 \| \| CCR5 \| AC220 \| -0.16038 \| 3.29E-05 \| \| CCR5 \| AG-014699 \| 0.091315 \| 0.025218 \| \| CCR5 \| AICAR \| -0.12711 \| 0.000854 \| \| CCR5 \| AKT inhibitor VIII \| 0.036451 \| 0.445459 \| \| CCR5 \| AMG-706 \| 0.009003 \| 0.912377 \| \| CCR5 \| AP-24534 \| -0.1218 \| 0.001528 \| \| CCR5 \| AR-42 \| -0.19557 \| 1.54E-08 \| \| CCR5 \| AS601245 \| 0.008689 \| 0.904192 \| \| CCR5 \| AS605240 \| -0.15067 \| 7.52E-05 \| \| CCR5 \| AT-7519 \| -0.19336 \| 1.95E-08 \| \| CCR5 \| ATRA \| -0.08354 \| 0.060892 \| \| CCR5 \| AUY922 \| -0.05356 \| 0.291331 \| \| CCR5 \| AZ628 \| -0.03271 \| 0.705672 \| \| CCR5 \| AZD6482 \| -0.03293 \| 0.511932 \| \| CCR5 \| AZD7762 \| -0.16694 \| 8.69E-06 \| \| CCR5 \| AZD8055 \| -0.21613 \| 4.53E-09 \| \| CCR5 \| Afatinib \| 0.05359 \| 0.156862 \| \| CCR5 \| Axitinib \| -0.06426 \| 0.193748 \| \| CCR5 \| BAY 61-3606 \| -0.15444 \| 4.63E-05 \| \| CCR5 \| BEZ235 \| -0.03835 \| 0.451588 \| \| CCR5 \| BHG712 \| -0.18644 \| 7.37E-08 \| \| CCR5 \| BI-2536 \| -0.03238 \| 0.793459 \| \| CCR5 \| BIRB 0796 \| 0.019791 \| 0.735256 \| \| CCR5 \| BIX02189 \| -0.16977 \| 1.23E-06 \| \| CCR5 \| BMS-509744 \| 0.015164 \| 0.892917 \| \| CCR5 \| BMS-536924 \| -0.02588 \| 0.743213 \| \| CCR5 \| BMS-708163 \| 0.064742 \| 0.105287 \| \| CCR5 \| BMS-754807 \| 0.049094 \| 0.365237 \| \| CCR5 \| BMS345541 \| -0.17497 \| 4.5E-07 \| \| CCR5 \| BX-795 \| -0.11625 \| 0.004327 \| \| CCR5 \| BX-912 \| -0.19417 \| 1.42E-08 \| \| CCR5 \| Belinostat \| -0.16555 \| 3.45E-06 \| \| CCR5 \| Bexarotene \| -0.03112 \| 0.70163 \| \| CCR5 \| Bicalutamide \| 0.049487 \| 0.268618 \| \| CCR5 \| Bleomycin \| -0.00779 \| 0.91589 \| \| CCR5 \| Bleomycin (50 uM) \| 0.079108 \| 0.029307 \| \| CCR5 \| Bortezomib \| 0.006856 \| 0.950856 \| \| CCR5 \| Bosutinib \| 0.017398 \| 0.789504 \| \| CCR5 \| Bryostatin 1 \| 0.031821 \| 0.570333 \| \| CCR5 \| CAL-101 \| -0.17571 \| 6.91E-07 \| \| CCR5 \| CAY10603 \| -0.19551 \| 1.45E-08 \| \| CCR5 \| CCT007093 \| 0.04917 \| 0.264243 \| \| CCR5 \| CCT018159 \| -0.01844 \| 0.761227 \| \| CCR5 \| CEP-701 \| -0.13634 \| 0.000353 \| \| CCR5 \| CGP-082996 \| -0.02509 \| 0.835823 \| \| CCR5 \| CGP-60474 \| -0.07561 \| 0.361162 \| \| CCR5 \| CH5424802 \| -0.10792 \| 0.017943 \| \| CCR5 \| CHIR-99021 \| 0.044007 \| 0.266153 \| \| CCR5 \| CI-1040 \| 0.00552 \| 0.921291 \| \| CCR5 \| CMK \| -0.03459 \| 0.797205 \| \| CCR5 \| CP466722 \| -0.20733 \| 1.44E-09 \| \| CCR5 \| CP724714 \| -0.0049 \| 0.947568 \| \| CCR5 \| CUDC-101 \| -0.20174 \| 8.12E-09 \| \| CCR5 \| CX-5461 \| -0.11493 \| 0.001356 \| \| CCR5 \| Camptothecin \| -0.08096 \| 0.052864 \| \| CCR5 \| Cetuximab \| 0.065979 \| 0.104194 \| \| CCR5 \| Cisplatin \| -0.00069 \| 0.991978 \| \| CCR5 \| Crizotinib \| -0.0645 \| 0.641555 \| \| CCR5 \| Cyclopamine \| -0.02951 \| 0.816889 \| \| CCR5 \| Cytarabine \| 0.040896 \| 0.432677 \| \| CCR5 \| DMOG \| -0.13326 \| 0.000756 \| \| CCR5 \| Dabrafenib \| 0.013494 \| 0.78807 \| \| CCR5 \| Dasatinib \| 0.072572 \| 0.285144 \| \| CCR5 \| Docetaxel \| 0.139389 \| 0.000149 \| \| CCR5 \| Doxorubicin \| -0.00575 \| 0.946538 \| \| CCR5 \| EHT 1864 \| -0.02723 \| 0.717254 \| \| CCR5 \| EKB-569 \| -0.09899 \| 0.009645 \| \| CCR5 \| EX-527 \| 0.000208 \| 0.998966 \| \| CCR5 \| Elesclomol \| 0.025662 \| 0.600271 \| \| CCR5 \| Embelin \| -0.01169 \| 0.883082 \| \| CCR5 \| Epothilone B \| 0.023577 \| 0.679153 \| \| CCR5 \| Erlotinib \| 0.100891 \| 0.157463 \| \| CCR5 \| Etoposide \| -0.06062 \| 0.185108 \| \| CCR5 \| FH535 \| 0.001861 \| 0.978872 \| \| CCR5 \| FK866 \| -0.15782 \| 7.11E-06 \| \| CCR5 \| FMK \| -0.09602 \| 0.080482 \| \| CCR5 \| FR-180204 \| -0.05569 \| 0.264668 \| \| CCR5 \| FTI-277 \| 0.053835 \| 0.200591 \| \| CCR5 \| Foretinib \| -0.08711 \| 0.025531 \| \| CCR5 \| GDC0449 \| -0.01361 \| 0.917544 \| \| CCR5 \| GDC0941 \| -0.01185 \| 0.869159 \| \| CCR5 \| GNF-2 \| 0.03482 \| 0.897448 \| \| CCR5 \| GSK-650394 \| -0.04451 \| 0.532132 \| \| CCR5 \| GSK1070916 \| -0.14691 \| 3.55E-05 \| \| CCR5 \| GSK1904529A \| 0.069572 \| 0.115822 \| \| CCR5 \| GSK2126458 \| -0.13256 \| 0.000234 \| \| CCR5 \| GSK269962A \| -0.08184 \| 0.066367 \| \| CCR5 \| GSK429286A \| -0.14453 \| 0.000103 \| \| CCR5 \| GSK690693 \| -0.08677 \| 0.017858 \| \| CCR5 \| GW 441756 \| -0.00699 \| 0.989313 \| \| CCR5 \| GW-2580 \| -0.07059 \| 0.586362 \| \| CCR5 \| GW843682X \| -0.04442 \| 0.649424 \| \| CCR5 \| Gefitinib \| 0.073498 \| 0.072619 \| \| CCR5 \| Gemcitabine \| -0.07303 \| 0.113902 \| \| CCR5 \| Genentech Cpd 10 \| -0.1263 \| 0.000396 \| \| CCR5 \| HG-5-113-01 \| -0.09423 \| 0.149063 \| \| CCR5 \| HG-5-88-01 \| -0.01113 \| 0.959924 \| \| CCR5 \| HG-6-64-1 \| -0.05379 \| 0.254944 \| \| CCR5 \| I-BET-762 \| -0.27757 \| 9.52E-17 \| \| CCR5 \| IOX2 \| 0.058348 \| 0.291693 \| \| CCR5 \| IPA-3 \| -0.14749 \| 7.55E-05 \| \| CCR5 \| Imatinib \| -0.00957 \| 0.966914 \| \| CCR5 \| Ispinesib Mesylate \| -0.11821 \| 0.000947 \| \| CCR5 \| JNJ-26854165 \| -0.03437 \| 0.525119 \| \| CCR5 \| JNK Inhibitor VIII \| 0.096301 \| 0.018338 \| \| CCR5 \| JNK-9L \| -0.0736 \| 0.1448 \| \| CCR5 \| JQ1 \| -0.11298 \| 0.00411 \| \| CCR5 \| JQ12 \| -0.09114 \| 0.060191 \| \| CCR5 \| JW-7-24-1 \| -0.19417 \| 1.63E-08 \| \| CCR5 \| JW-7-52-1 \| 0.002088 \| 0.99011 \| \| CCR5 \| KIN001-055 \| -0.0299 \| 0.671617 \| \| CCR5 \| KIN001-102 \| -0.19806 \| 7.61E-09 \| \| CCR5 \| KIN001-135 \| 0.129849 \| 0.134889 \| \| CCR5 \| KIN001-236 \| -0.15815 \| 7.83E-06 \| \| CCR5 \| KIN001-244 \| -0.18289 \| 1.85E-07 \| \| CCR5 \| KIN001-260 \| -0.23193 \| 1.4E-11 \| \| CCR5 \| KIN001-266 \| -0.07205 \| 0.110325 \| \| CCR5 \| KIN001-270 \| -0.19676 \| 5.26E-08 \| \| CCR5 \| KU-55933 \| -0.02863 \| 0.678569 \| \| CCR5 \| LAQ824 \| -0.15276 \| 3.92E-05 \| \| CCR5 \| LFM-A13 \| 0.048424 \| 0.339664 \| \| CCR5 \| LY317615 \| -0.08407 \| 0.041311 \| \| CCR5 \| Lapatinib \| 0.098676 \| 0.115476 \| \| CCR5 \| Lenalidomide \| -0.21773 \| 2.63E-07 \| \| CCR5 \| Linifanib \| -0.15122 \| 0.00011 \| \| CCR5 \| Lisitinib \| 0.023693 \| 0.752157 \| \| CCR5 \| MG-132 \| 0.06109 \| 0.497949 \| \| CCR5 \| MK-2206 \| -0.09423 \| 0.043912 \| \| CCR5 \| MLN4924 \| -0.02221 \| 0.729487 \| \| CCR5 \| MP470 \| -0.07065 \| 0.094909 \| \| CCR5 \| MPS-1-IN-1 \| -0.14329 \| 6.9E-05 \| \| CCR5 \| MS-275 \| -0.15037 \| 0.032215 \| \| CCR5 \| Masitinib \| -0.16558 \| 3.25E-06 \| \| CCR5 \| Methotrexate \| -0.23476 \| 3.01E-11 \| \| CCR5 \| Midostaurin \| -0.01796 \| 0.725732 \| \| CCR5 \| Mitomycin C \| -0.0757 \| 0.106405 \| \| CCR5 \| NG-25 \| -0.17815 \| 2.62E-07 \| \| CCR5 \| NPK76-II-72-1 \| -0.192 \| 1.72E-08 \| \| CCR5 \| NSC-207895 \| -0.04542 \| 0.320142 \| \| CCR5 \| NSC-87877 \| 0.061953 \| 0.245717 \| \| CCR5 \| NU-7441 \| -0.06187 \| 0.344382 \| \| CCR5 \| Navitoclax \| -0.08354 \| 0.025384 \| \| CCR5 \| Nilotinib \| -0.06218 \| 0.203349 \| \| CCR5 \| Nutlin-3a (-) \| -0.1592 \| 8.71E-05 \| \| CCR5 \| OSI-027 \| -0.20269 \| 4.84E-09 \| \| CCR5 \| OSI-930 \| -0.18476 \| 2.66E-07 \| \| CCR5 \| OSU-03012 \| -0.03794 \| 0.465326 \| \| CCR5 \| Obatoclax Mesylate \| -0.08479 \| 0.051993 \| \| CCR5 \| Olaparib \| 0.055449 \| 0.202667 \| \| CCR5 \| PAC-1 \| -0.08997 \| 0.02532 \| \| CCR5 \| PD-0325901 \| 0.035327 \| 0.405893 \| \| CCR5 \| PD-0332991 \| -0.01298 \| 0.855664 \| \| CCR5 \| PD-173074 \| -0.02037 \| 0.911373 \| \| CCR5 \| PF-4708671 \| 0.003312 \| 0.985343 \| \| CCR5 \| PF-562271 \| 0.008738 \| 0.903815 \| \| CCR5 \| PFI-1 \| -0.15074 \| 0.000199 \| \| CCR5 \| PHA-665752 \| 0.025081 \| 0.883759 \| \| CCR5 \| PHA-793887 \| -0.21598 \| 1.97E-10 \| \| CCR5 \| PI-103 \| -0.1759 \| 4.59E-07 \| \| CCR5 \| PIK-93 \| -0.18099 \| 1.32E-07 \| \| CCR5 \| PLX4720 \| 0.044901 \| 0.288192 \| \| CCR5 \| Paclitaxel \| -0.05448 \| 0.636032 \| \| CCR5 \| Parthenolide \| -0.03637 \| 0.753571 \| \| CCR5 \| Pazopanib \| -0.00848 \| 0.903935 \| \| CCR5 \| Phenformin \| -0.19164 \| 6.1E-08 \| \| CCR5 \| Pyrimethamine \| -0.01004 \| 0.948557 \| \| CCR5 \| QL-VIII-58 \| -0.00754 \| 0.944402 \| \| CCR5 \| QL-X-138 \| -0.15499 \| 1.14E-05 \| \| CCR5 \| QL-XI-92 \| -0.21492 \| 4.18E-10 \| \| CCR5 \| QL-XII-47 \| -0.14324 \| 0.000116 \| \| CCR5 \| QL-XII-61 \| -0.11659 \| 0.050736 \| \| CCR5 \| QS11 \| -0.00752 \| 0.930074 \| \| CCR5 \| RDEA119 \| 0.025778 \| 0.524019 \| \| CCR5 \| RO-3306 \| 0.048647 \| 0.255015 \| \| CCR5 \| Rapamycin \| -0.04955 \| 0.693464 \| \| CCR5 \| Roscovitine \| -0.08886 \| 0.558476 \| \| CCR5 \| Ruxolitinib \| -0.20608 \| 3.34E-08 \| \| CCR5 \| S-Trityl-L-cysteine \| -0.09882 \| 0.191516 \| \| CCR5 \| SB 216763 \| 0.035347 \| 0.497364 \| \| CCR5 \| SB 505124 \| 0.007548 \| 0.947078 \| \| CCR5 \| SB52334 \| 0.041052 \| 0.401323 \| \| CCR5 \| SB590885 \| 0.041206 \| 0.420697 \| \| CCR5 \| SGC0946 \| 0.035485 \| 0.525293 \| \| CCR5 \| SL 0101-1 \| 0.054318 \| 0.468833 \| \| CCR5 \| SN-38 \| -0.06601 \| 0.11906 \| \| CCR5 \| SNX-2112 \| -0.17636 \| 4.3E-07 \| \| CCR5 \| STF-62247 \| -0.15745 \| 2.58E-05 \| \| CCR5 \| Salubrinal \| -0.07441 \| 0.371354 \| \| CCR5 \| Saracatinib \| 0.07194 \| 0.383554 \| \| CCR5 \| Shikonin \| -0.018 \| 0.776033 \| \| CCR5 \| Sorafenib \| -0.14423 \| 0.069384 \| \| CCR5 \| Sunitinib \| -0.15596 \| 0.015108 \| \| CCR5 \| T0901317 \| -0.11694 \| 0.002201 \| \| CCR5 \| TAE684 \| -0.01206 \| 0.940738 \| \| CCR5 \| TAK-715 \| -0.19587 \| 1.7E-08 \| \| CCR5 \| TG101348 \| -0.24311 \| 7.72E-13 \| \| CCR5 \| TGX221 \| 0.059143 \| 0.377133 \| \| CCR5 \| THZ-2-102-1 \| -0.1843 \| 1.24E-07 \| \| CCR5 \| THZ-2-49 \| -0.17601 \| 6.43E-07 \| \| CCR5 \| TL-1-85 \| -0.1847 \| 9.39E-08 \| \| CCR5 \| TL-2-105 \| -0.11123 \| 0.00217 \| \| CCR5 \| TPCA-1 \| -0.26893 \| 1.19E-15 \| \| CCR5 \| TW 37 \| 0.02706 \| 0.598504 \| \| CCR5 \| Talazoparib \| -0.01763 \| 0.734545 \| \| CCR5 \| Tamoxifen \| 0.012892 \| 0.935146 \| \| CCR5 \| Temozolomide \| -0.17795 \| 1.27E-05 \| \| CCR5 \| Temsirolimus \| -0.09431 \| 0.029887 \| \| CCR5 \| Thapsigargin \| 0.016723 \| 0.840616 \| \| CCR5 \| Tipifarnib \| -0.1066 \| 0.033861 \| \| CCR5 \| Tivozanib \| -0.07598 \| 0.124245 \| \| CCR5 \| Trametinib \| 0.067578 \| 0.072972 \| \| CCR5 \| Tubastatin A \| -0.18952 \| 3.78E-08 \| \| CCR5 \| UNC0638 \| -0.16617 \| 1E-06 \| \| CCR5 \| UNC1215 \| 0.042359 \| 0.411871 \| \| CCR5 \| VNLG/124 \| -0.11964 \| 0.001694 \| \| CCR5 \| VX-11e \| -0.08758 \| 0.04214 \| \| CCR5 \| VX-680 \| -0.05971 \| 0.530062 \| \| CCR5 \| VX-702 \| -0.04969 \| 0.784914 \| \| CCR5 \| Veliparib \| 0.028531 \| 0.695814 \| \| CCR5 \| Vinblastine \| -0.08553 \| 0.040685 \| \| CCR5 \| Vinorelbine \| -0.04667 \| 0.394962 \| \| CCR5 \| Vorinostat \| -0.21584 \| 1.17E-09 \| \| CCR5 \| WH-4-023 \| 0.058057 \| 0.468488 \| \| CCR5 \| WZ-1-84 \| 0.035164 \| 0.685979 \| \| CCR5 \| WZ3105 \| -0.15082 \| 1.42E-05 \| \| CCR5 \| XAV939 \| 0.085988 \| 0.024933 \| \| CCR5 \| XL-184 \| -0.10417 \| 0.011509 \| \| CCR5 \| XMD11-85h \| -0.09122 \| 0.497138 \| \| CCR5 \| XMD13-2 \| -0.1963 \| 1.18E-08 \| \| CCR5 \| XMD14-99 \| -0.22904 \| 5.85E-11 \| \| CCR5 \| XMD15-27 \| -0.13557 \| 0.000767 \| \| CCR5 \| XMD8-85 \| -0.16445 \| 0.015518 \| \| CCR5 \| XMD8-92 \| -0.07193 \| 0.472288 \| \| CCR5 \| Y-39983 \| -0.1997 \| 1.18E-08 \| \| CCR5 \| YK 4-279 \| -0.09236 \| 0.051505 \| \| CCR5 \| YM155 \| -0.04334 \| 0.408965 \| \| CCR5 \| YM201636 \| -0.12961 \| 0.000273 \| \| CCR5 \| Z-LLNle-CHO \| -0.05145 \| 0.515075 \| \| CCR5 \| ZG-10 \| -0.08967 \| 0.151743 \| \| CCR5 \| ZM-447439 \| -0.09597 \| 0.023827 \| \| CCR5 \| ZSTK474 \| -0.17577 \| 4.83E-07 \| \| CCR5 \| Zibotentan \| 0.02795 \| 0.926063 \| \| CCR5 \| piperlongumine \| -0.04412 \| 0.322665 \| \| CCR5 \| rTRAIL \| 0.033931 \| 0.661639 \| \| CCR5 \| selumetinib \| 0.063638 \| 0.09001 \| \| CXCL10 \| (5Z)-7-Oxozeaenol \| 0.010049 \| 0.845013 \| \| CXCL10 \| 17-AAG \| 0.072927 \| 0.052732 \| \| CXCL10 \| 5-Fluorouracil \| -0.07743 \| 0.03446 \| \| CXCL10 \| 681640 \| -0.03285 \| 0.606651 \| \| CXCL10 \| A-443654 \| 0.064823 \| 0.68977 \| \| CXCL10 \| A-770041 \| 0.069617 \| 0.385334 \| \| CXCL10 \| AC220 \| -0.00539 \| 0.937752 \| \| CXCL10 \| AG-014699 \| 0.003001 \| 0.955267 \| \| CXCL10 \| AICAR \| -0.12798 \| 0.000784 \| \| CXCL10 \| AKT inhibitor VIII \| -0.00697 \| 0.901386 \| \| CXCL10 \| AMG-706 \| 0.062018 \| 0.261837 \| \| CXCL10 \| AP-24534 \| 0.049829 \| 0.237387 \| \| CXCL10 \| AR-42 \| -0.06 \| 0.101591 \| \| CXCL10 \| AS601245 \| 0.022123 \| 0.722755 \| \| CXCL10 \| AS605240 \| -0.01949 \| 0.691542 \| \| CXCL10 \| AT-7519 \| -0.12244 \| 0.000502 \| \| CXCL10 \| ATRA \| -0.0614 \| 0.193339 \| \| CXCL10 \| AUY922 \| -0.03783 \| 0.492889 \| \| CXCL10 \| AZ628 \| 0.098049 \| 0.165172 \| \| CXCL10 \| AZD6482 \| -0.02914 \| 0.574414 \| \| CXCL10 \| AZD7762 \| 0.013612 \| 0.77328 \| \| CXCL10 \| AZD8055 \| -0.04551 \| 0.267027 \| \| CXCL10 \| Afatinib \| -0.05128 \| 0.1771 \| \| CXCL10 \| Axitinib \| 0.06163 \| 0.215756 \| \| CXCL10 \| BAY 61-3606 \| -0.0087 \| 0.857715 \| \| CXCL10 \| BEZ235 \| -0.01267 \| 0.831937 \| \| CXCL10 \| BHG712 \| 0.026164 \| 0.502697 \| \| CXCL10 \| BI-2536 \| 0.084196 \| 0.380658 \| \| CXCL10 \| BIRB 0796 \| 0.057854 \| 0.248907 \| \| CXCL10 \| BIX02189 \| 0.003924 \| 0.928131 \| \| CXCL10 \| BMS-509744 \| 0.059764 \| 0.522189 \| \| CXCL10 \| BMS-536924 \| 0.008514 \| 0.93128 \| \| CXCL10 \| BMS-708163 \| 0.0186 \| 0.678357 \| \| CXCL10 \| BMS-754807 \| 0.063242 \| 0.222239 \| \| CXCL10 \| BMS345541 \| -0.04273 \| 0.254704 \| \| CXCL10 \| BX-795 \| 0.01018 \| 0.862891 \| \| CXCL10 \| BX-912 \| -0.00887 \| 0.824835 \| \| CXCL10 \| Belinostat \| -0.05842 \| 0.123469 \| \| CXCL10 \| Bexarotene \| 0.032409 \| 0.686667 \| \| CXCL10 \| Bicalutamide \| -0.01517 \| 0.771912 \| \| CXCL10 \| Bleomycin \| -0.0368 \| 0.529237 \| \| CXCL10 \| Bleomycin (50 uM) \| -0.03024 \| 0.432543 \| \| CXCL10 \| Bortezomib \| 0.002942 \| 0.979345 \| \| CXCL10 \| Bosutinib \| 0.002204 \| 0.976218 \| \| CXCL10 \| Bryostatin 1 \| 0.030543 \| 0.588269 \| \| CXCL10 \| CAL-101 \| -0.06926 \| 0.06658 \| \| CXCL10 \| CAY10603 \| -0.06362 \| 0.080828 \| \| CXCL10 \| CCT007093 \| 0.0072 \| 0.892727 \| \| CXCL10 \| CCT018159 \| -0.07919 \| 0.089773 \| \| CXCL10 \| CEP-701 \| 0.008849 \| 0.85498 \| \| CXCL10 \| CGP-082996 \| 0.024076 \| 0.843485 \| \| CXCL10 \| CGP-60474 \| -0.03942 \| 0.681296 \| \| CXCL10 \| CH5424802 \| -0.01152 \| 0.882741 \| \| CXCL10 \| CHIR-99021 \| 0.050818 \| 0.193513 \| \| CXCL10 \| CI-1040 \| 0.025995 \| 0.591457 \| \| CXCL10 \| CMK \| -0.00967 \| 0.950848 \| \| CXCL10 \| CP466722 \| -0.05228 \| 0.156857 \| \| CXCL10 \| CP724714 \| -0.00849 \| 0.907924 \| \| CXCL10 \| CUDC-101 \| -0.06043 \| 0.106458 \| \| CXCL10 \| CX-5461 \| 0.005318 \| 0.901575 \| \| CXCL10 \| Camptothecin \| -0.05314 \| 0.230014 \| \| CXCL10 \| Cetuximab \| -0.00115 \| 0.980312 \| \| CXCL10 \| Cisplatin \| 0.003506 \| 0.958538 \| \| CXCL10 \| Crizotinib \| 0.047139 \| 0.759524 \| \| CXCL10 \| Cyclopamine \| 0.013977 \| 0.922617 \| \| CXCL10 \| Cytarabine \| 0.070036 \| 0.139235 \| \| CXCL10 \| DMOG \| 0.021309 \| 0.680862 \| \| CXCL10 \| Dabrafenib \| 0.05246 \| 0.226636 \| \| CXCL10 \| Dasatinib \| 0.115461 \| 0.071158 \| \| CXCL10 \| Docetaxel \| 0.007506 \| 0.858335 \| \| CXCL10 \| Doxorubicin \| -0.04036 \| 0.536623 \| \| CXCL10 \| EHT 1864 \| 0.015755 \| 0.855617 \| \| CXCL10 \| EKB-569 \| -0.01003 \| 0.833958 \| \| CXCL10 \| EX-527 \| 0.021457 \| 0.863096 \| \| CXCL10 \| Elesclomol \| 0.078862 \| 0.064418 \| \| CXCL10 \| Embelin \| 0.038251 \| 0.532052 \| \| CXCL10 \| Epothilone B \| 0.000532 \| 0.993409 \| \| CXCL10 \| Erlotinib \| 0.100228 \| 0.160622 \| \| CXCL10 \| Etoposide \| -0.03544 \| 0.485186 \| \| CXCL10 \| FH535 \| 0.067045 \| 0.141902 \| \| CXCL10 \| FK866 \| -0.07689 \| 0.035394 \| \| CXCL10 \| FMK \| 0.049116 \| 0.460679 \| \| CXCL10 \| FR-180204 \| 0.041957 \| 0.428923 \| \| CXCL10 \| FTI-277 \| 0.002585 \| 0.961207 \| \| CXCL10 \| Foretinib \| 0.015911 \| 0.742893 \| \| CXCL10 \| GDC0449 \| -0.04503 \| 0.639823 \| \| CXCL10 \| GDC0941 \| -0.03717 \| 0.526384 \| \| CXCL10 \| GNF-2 \| 0.085006 \| 0.66075 \| \| CXCL10 \| GSK-650394 \| 0.039003 \| 0.600399 \| \| CXCL10 \| GSK1070916 \| 0.014877 \| 0.717836 \| \| CXCL10 \| GSK1904529A \| 0.021066 \| 0.684497 \| \| CXCL10 \| GSK2126458 \| -0.09295 \| 0.011653 \| \| CXCL10 \| GSK269962A \| 0.075244 \| 0.09701 \| \| CXCL10 \| GSK429286A \| 0.04849 \| 0.250812 \| \| CXCL10 \| GSK690693 \| -0.00415 \| 0.923703 \| \| CXCL10 \| GW 441756 \| 0.005772 \| 0.991299 \| \| CXCL10 \| GW-2580 \| 0.020595 \| 0.967411 \| \| CXCL10 \| GW843682X \| 0.063212 \| 0.48083 \| \| CXCL10 \| Gefitinib \| -0.0434 \| 0.308956 \| \| CXCL10 \| Gemcitabine \| -0.0528 \| 0.277932 \| \| CXCL10 \| Genentech Cpd 10 \| -0.0162 \| 0.698262 \| \| CXCL10 \| HG-5-113-01 \| 0.004277 \| 0.96606 \| \| CXCL10 \| HG-5-88-01 \| 0.011149 \| 0.959673 \| \| CXCL10 \| HG-6-64-1 \| 0.049701 \| 0.299671 \| \| CXCL10 \| I-BET-762 \| -0.0255 \| 0.497068 \| \| CXCL10 \| IOX2 \| 0.028252 \| 0.653376 \| \| CXCL10 \| IPA-3 \| -0.03838 \| 0.354352 \| \| CXCL10 \| Imatinib \| 0.078878 \| 0.54186 \| \| CXCL10 \| Ispinesib Mesylate \| -0.08264 \| 0.023733 \| \| CXCL10 \| JNJ-26854165 \| 0.006399 \| 0.925211 \| \| CXCL10 \| JNK Inhibitor VIII \| 0.008794 \| 0.861694 \| \| CXCL10 \| JNK-9L \| -0.05348 \| 0.323129 \| \| CXCL10 \| JQ1 \| -0.0588 \| 0.163537 \| \| CXCL10 \| JQ12 \| 0.011638 \| 0.864001 \| \| CXCL10 \| JW-7-24-1 \| -0.06091 \| 0.095225 \| \| CXCL10 \| JW-7-52-1 \| 0.022928 \| 0.853659 \| \| CXCL10 \| KIN001-055 \| 0.02522 \| 0.731237 \| \| CXCL10 \| KIN001-102 \| -0.05907 \| 0.104711 \| \| CXCL10 \| KIN001-135 \| 0.072941 \| 0.46129 \| \| CXCL10 \| KIN001-236 \| -0.03867 \| 0.31999 \| \| CXCL10 \| KIN001-244 \| -0.04851 \| 0.202662 \| \| CXCL10 \| KIN001-260 \| -0.0923 \| 0.010422 \| \| CXCL10 \| KIN001-266 \| -0.04273 \| 0.379766 \| \| CXCL10 \| KIN001-270 \| -0.03135 \| 0.457402 \| \| CXCL10 \| KU-55933 \| -0.08369 \| 0.102698 \| \| CXCL10 \| LAQ824 \| -0.02226 \| 0.610088 \| \| CXCL10 \| LFM-A13 \| 0.010146 \| 0.871279 \| \| CXCL10 \| LY317615 \| -0.01475 \| 0.773665 \| \| CXCL10 \| Lapatinib \| 0.085895 \| 0.175136 \| \| CXCL10 \| Lenalidomide \| 0.00107 \| 0.992485 \| \| CXCL10 \| Linifanib \| 0.028824 \| 0.613517 \| \| CXCL10 \| Lisitinib \| 0.041268 \| 0.529382 \| \| CXCL10 \| MG-132 \| 0.000943 \| 0.994277 \| \| CXCL10 \| MK-2206 \| 0.032688 \| 0.553772 \| \| CXCL10 \| MLN4924 \| -0.00274 \| 0.970719 \| \| CXCL10 \| MP470 \| 0.028229 \| 0.543237 \| \| CXCL10 \| MPS-1-IN-1 \| -0.01518 \| 0.721858 \| \| CXCL10 \| MS-275 \| 0.041356 \| 0.651084 \| \| CXCL10 \| Masitinib \| -0.00591 \| 0.896501 \| \| CXCL10 \| Methotrexate \| -0.08191 \| 0.027766 \| \| CXCL10 \| Midostaurin \| 0.062501 \| 0.152414 \| \| CXCL10 \| Mitomycin C \| -0.03451 \| 0.528032 \| \| CXCL10 \| NG-25 \| 0.002993 \| 0.943857 \| \| CXCL10 \| NPK76-II-72-1 \| -0.03119 \| 0.404979 \| \| CXCL10 \| NSC-207895 \| 0.004781 \| 0.936431 \| \| CXCL10 \| NSC-87877 \| 0.05406 \| 0.319269 \| \| CXCL10 \| NU-7441 \| -0.03808 \| 0.613362 \| \| CXCL10 \| Navitoclax \| -0.06007 \| 0.115047 \| \| CXCL10 \| Nilotinib \| 0.034604 \| 0.527868 \| \| CXCL10 \| Nutlin-3a (-) \| -0.0244 \| 0.65152 \| \| CXCL10 \| OSI-027 \| -0.11193 \| 0.001686 \| \| CXCL10 \| OSI-930 \| -0.04862 \| 0.221816 \| \| CXCL10 \| OSU-03012 \| -0.03598 \| 0.49256 \| \| CXCL10 \| Obatoclax Mesylate \| -0.03214 \| 0.52493 \| \| CXCL10 \| Olaparib \| -0.00816 \| 0.877671 \| \| CXCL10 \| PAC-1 \| -0.04804 \| 0.265863 \| \| CXCL10 \| PD-0325901 \| 0.049853 \| 0.227771 \| \| CXCL10 \| PD-0332991 \| 0.029642 \| 0.63058 \| \| CXCL10 \| PD-173074 \| 0.031435 \| 0.846233 \| \| CXCL10 \| PF-4708671 \| -0.04219 \| 0.667571 \| \| CXCL10 \| PF-562271 \| 0.042878 \| 0.448106 \| \| CXCL10 \| PFI-1 \| -0.06518 \| 0.153788 \| \| CXCL10 \| PHA-665752 \| 0.054412 \| 0.713801 \| \| CXCL10 \| PHA-793887 \| -0.07506 \| 0.034913 \| \| CXCL10 \| PI-103 \| -0.09836 \| 0.006104 \| \| CXCL10 \| PIK-93 \| -0.08091 \| 0.023177 \| \| CXCL10 \| PLX4720 \| 0.021894 \| 0.632243 \| \| CXCL10 \| Paclitaxel \| 0.013236 \| 0.934662 \| \| CXCL10 \| Parthenolide \| 0.023749 \| 0.850389 \| \| CXCL10 \| Pazopanib \| 0.048851 \| 0.370565 \| \| CXCL10 \| Phenformin \| -0.03615 \| 0.356757 \| \| CXCL10 \| Pyrimethamine \| -0.00708 \| 0.964938 \| \| CXCL10 \| QL-VIII-58 \| -0.00183 \| 0.987972 \| \| CXCL10 \| QL-X-138 \| -0.06148 \| 0.099465 \| \| CXCL10 \| QL-XI-92 \| -0.03714 \| 0.332475 \| \| CXCL10 \| QL-XII-47 \| -0.06065 \| 0.128867 \| \| CXCL10 \| QL-XII-61 \| -0.01799 \| 0.828196 \| \| CXCL10 \| QS11 \| 0.031232 \| 0.641491 \| \| CXCL10 \| RDEA119 \| 0.022912 \| 0.574295 \| \| CXCL10 \| RO-3306 \| -0.01754 \| 0.705058 \| \| CXCL10 \| Rapamycin \| -0.02129 \| 0.894881 \| \| CXCL10 \| Roscovitine \| 0.00196 \| 0.995508 \| \| CXCL10 \| Ruxolitinib \| -0.07594 \| 0.096366 \| \| CXCL10 \| S-Trityl-L-cysteine \| 0.025621 \| 0.795292 \| \| CXCL10 \| SB 216763 \| 0.043384 \| 0.392723 \| \| CXCL10 \| SB 505124 \| 0.052598 \| 0.453373 \| \| CXCL10 \| SB52334 \| 0.041038 \| 0.401449 \| \| CXCL10 \| SB590885 \| 0.015154 \| 0.790183 \| \| CXCL10 \| SGC0946 \| 0.02342 \| 0.703824 \| \| CXCL10 \| SL 0101-1 \| -0.01413 \| 0.882079 \| \| CXCL10 \| SN-38 \| -0.05243 \| 0.232625 \| \| CXCL10 \| SNX-2112 \| -0.11658 \| 0.001087 \| \| CXCL10 \| STF-62247 \| -0.04025 \| 0.338218 \| \| CXCL10 \| Salubrinal \| -0.06281 \| 0.464387 \| \| CXCL10 \| Saracatinib \| 0.091079 \| 0.252022 \| \| CXCL10 \| Shikonin \| -0.05761 \| 0.259017 \| \| CXCL10 \| Sorafenib \| 0.046263 \| 0.697167 \| \| CXCL10 \| Sunitinib \| 0.041818 \| 0.611445 \| \| CXCL10 \| T0901317 \| -0.01307 \| 0.784637 \| \| CXCL10 \| TAE684 \| 0.05861 \| 0.604289 \| \| CXCL10 \| TAK-715 \| -0.05012 \| 0.180343 \| \| CXCL10 \| TG101348 \| -0.07081 \| 0.050745 \| \| CXCL10 \| TGX221 \| 0.014655 \| 0.847347 \| \| CXCL10 \| THZ-2-102-1 \| -0.08713 \| 0.016035 \| \| CXCL10 \| THZ-2-49 \| -0.02757 \| 0.485804 \| \| CXCL10 \| TL-1-85 \| 0.008198 \| 0.84542 \| \| CXCL10 \| TL-2-105 \| 0.007213 \| 0.866846 \| \| CXCL10 \| TPCA-1 \| -0.07678 \| 0.031718 \| \| CXCL10 \| TW 37 \| 0.033608 \| 0.496323 \| \| CXCL10 \| Talazoparib \| -0.01439 \| 0.785886 \| \| CXCL10 \| Tamoxifen \| -0.04305 \| 0.694849 \| \| CXCL10 \| Temozolomide \| -0.07113 \| 0.223112 \| \| CXCL10 \| Temsirolimus \| -0.00193 \| 0.979663 \| \| CXCL10 \| Thapsigargin \| 0.040196 \| 0.543251 \| \| CXCL10 \| Tipifarnib \| 0.032183 \| 0.653563 \| \| CXCL10 \| Tivozanib \| 0.038281 \| 0.523129 \| \| CXCL10 \| Trametinib \| 0.036189 \| 0.360594 \| \| CXCL10 \| Tubastatin A \| -0.01866 \| 0.63589 \| \| CXCL10 \| UNC0638 \| -0.03845 \| 0.29747 \| \| CXCL10 \| UNC1215 \| 0.031687 \| 0.563241 \| \| CXCL10 \| VNLG/124 \| -0.07158 \| 0.074838 \| \| CXCL10 \| VX-11e \| 0.023832 \| 0.660647 \| \| CXCL10 \| VX-680 \| 0.086197 \| 0.329113 \| \| CXCL10 \| VX-702 \| 0.00169 \| 0.998201 \| \| CXCL10 \| Veliparib \| 0.004635 \| 0.961575 \| \| CXCL10 \| Vinblastine \| -0.01932 \| 0.704531 \| \| CXCL10 \| Vinorelbine \| -0.02719 \| 0.656229 \| \| CXCL10 \| Vorinostat \| -0.04887 \| 0.19712 \| \| CXCL10 \| WH-4-023 \| 0.069653 \| 0.370342 \| \| CXCL10 \| WZ-1-84 \| 0.086317 \| 0.252199 \| \| CXCL10 \| WZ3105 \| -0.06732 \| 0.062939 \| \| CXCL10 \| XAV939 \| 0.005029 \| 0.914672 \| \| CXCL10 \| XL-184 \| 0.022758 \| 0.665851 \| \| CXCL10 \| XMD11-85h \| -0.02084 \| 0.921196 \| \| CXCL10 \| XMD13-2 \| -0.08207 \| 0.022773 \| \| CXCL10 \| XMD14-99 \| -0.02671 \| 0.517889 \| \| CXCL10 \| XMD15-27 \| -0.01319 \| 0.816445 \| \| CXCL10 \| XMD8-85 \| -0.00583 \| 0.956711 \| \| CXCL10 \| XMD8-92 \| -0.05037 \| 0.650557 \| \| CXCL10 \| Y-39983 \| 0.014672 \| 0.730747 \| \| CXCL10 \| YK 4-279 \| -0.01545 \| 0.817231 \| \| CXCL10 \| YM155 \| -0.04517 \| 0.384176 \| \| CXCL10 \| YM201636 \| -0.02408 \| 0.548952 \| \| CXCL10 \| Z-LLNle-CHO \| -0.10245 \| 0.138797 \| \| CXCL10 \| ZG-10 \| 0.000863 \| 0.992584 \| \| CXCL10 \| ZM-447439 \| 0.043422 \| 0.365111 \| \| CXCL10 \| ZSTK474 \| -0.08969 \| 0.013059 \| \| CXCL10 \| Zibotentan \| -0.0341 \| 0.90116 \| \| CXCL10 \| piperlongumine \| 0.00515 \| 0.928371 \| \| CXCL10 \| rTRAIL \| 0.025033 \| 0.762182 \| \| CXCL10 \| selumetinib \| 0.019405 \| 0.643048 \| \| CXCL11 \| (5Z)-7-Oxozeaenol \| -0.00757 \| 0.883935 \| \| CXCL11 \| 17-AAG \| -0.01499 \| 0.718389 \| \| CXCL11 \| 5-Fluorouracil \| -0.04857 \| 0.197743 \| \| CXCL11 \| 681640 \| -0.04761 \| 0.430386 \| \| CXCL11 \| A-443654 \| 0.08701 \| 0.5541 \| \| CXCL11 \| A-770041 \| 0.01771 \| 0.863627 \| \| CXCL11 \| AC220 \| -0.01675 \| 0.773578 \| \| CXCL11 \| AG-014699 \| -0.00956 \| 0.851812 \| \| CXCL11 \| AICAR \| -0.06702 \| 0.09408 \| \| CXCL11 \| AKT inhibitor VIII \| -0.01429 \| 0.788982 \| \| CXCL11 \| AMG-706 \| 0.035182 \| 0.582569 \| \| CXCL11 \| AP-24534 \| -0.02941 \| 0.511355 \| \| CXCL11 \| AR-42 \| -0.04146 \| 0.267909 \| \| CXCL11 \| AS601245 \| -0.02279 \| 0.713194 \| \| CXCL11 \| AS605240 \| -0.01451 \| 0.774915 \| \| CXCL11 \| AT-7519 \| -0.08575 \| 0.016553 \| \| CXCL11 \| ATRA \| 0.031173 \| 0.557148 \| \| CXCL11 \| AUY922 \| -0.02941 \| 0.610772 \| \| CXCL11 \| AZ628 \| 0.062819 \| 0.412506 \| \| CXCL11 \| AZD6482 \| -0.0302 \| 0.556837 \| \| CXCL11 \| AZD7762 \| 0.016621 \| 0.719231 \| \| CXCL11 \| AZD8055 \| -0.02436 \| 0.573075 \| \| CXCL11 \| Afatinib \| -0.08159 \| 0.02614 \| \| CXCL11 \| Axitinib \| 0.025088 \| 0.677394 \| \| CXCL11 \| BAY 61-3606 \| 0.004469 \| 0.93 \| \| CXCL11 \| BEZ235 \| -0.02242 \| 0.685577 \| \| CXCL11 \| BHG712 \| -0.0263 \| 0.500348 \| \| CXCL11 \| BI-2536 \| 0.035327 \| 0.768445 \| \| CXCL11 \| BIRB 0796 \| 0.02546 \| 0.65421 \| \| CXCL11 \| BIX02189 \| 0.021282 \| 0.594396 \| \| CXCL11 \| BMS-509744 \| 2.42E-05 \| 0.999989 \| \| CXCL11 \| BMS-536924 \| 0.008172 \| 0.933832 \| \| CXCL11 \| BMS-708163 \| -0.01041 \| 0.823172 \| \| CXCL11 \| BMS-754807 \| 0.00892 \| 0.896967 \| \| CXCL11 \| BMS345541 \| -0.03173 \| 0.408133 \| \| CXCL11 \| BX-795 \| -0.02216 \| 0.674761 \| \| CXCL11 \| BX-912 \| -0.0375 \| 0.315166 \| \| CXCL11 \| Belinostat \| -0.05864 \| 0.122039 \| \| CXCL11 \| Bexarotene \| 0.035895 \| 0.645634 \| \| CXCL11 \| Bicalutamide \| -0.01782 \| 0.727458 \| \| CXCL11 \| Bleomycin \| -0.0208 \| 0.748638 \| \| CXCL11 \| Bleomycin (50 uM) \| -0.03545 \| 0.351956 \| \| CXCL11 \| Bortezomib \| 0.041815 \| 0.635794 \| \| CXCL11 \| Bosutinib \| -0.03933 \| 0.480114 \| \| CXCL11 \| Bryostatin 1 \| -0.00212 \| 0.97678 \| \| CXCL11 \| CAL-101 \| -0.02632 \| 0.521641 \| \| CXCL11 \| CAY10603 \| -0.03118 \| 0.413573 \| \| CXCL11 \| CCT007093 \| 0.021975 \| 0.654531 \| \| CXCL11 \| CCT018159 \| -0.00543 \| 0.941162 \| \| CXCL11 \| CEP-701 \| 0.028083 \| 0.526563 \| \| CXCL11 \| CGP-082996 \| 0.042482 \| 0.685064 \| \| CXCL11 \| CGP-60474 \| 0.04139 \| 0.66319 \| \| CXCL11 \| CH5424802 \| -0.0455 \| 0.429339 \| \| CXCL11 \| CHIR-99021 \| -0.01238 \| 0.778817 \| \| CXCL11 \| CI-1040 \| -0.01981 \| 0.693215 \| \| CXCL11 \| CMK \| 0.019632 \| 0.893889 \| \| CXCL11 \| CP466722 \| -0.02721 \| 0.481794 \| \| CXCL11 \| CP724714 \| -0.03237 \| 0.575666 \| \| CXCL11 \| CUDC-101 \| -0.05653 \| 0.132918 \| \| CXCL11 \| CX-5461 \| -0.01607 \| 0.695597 \| \| CXCL11 \| Camptothecin \| 0.00248 \| 0.967461 \| \| CXCL11 \| Cetuximab \| -0.03661 \| 0.392291 \| \| CXCL11 \| Cisplatin \| 0.003544 \| 0.957956 \| \| CXCL11 \| Crizotinib \| -0.00992 \| 0.965845 \| \| CXCL11 \| Cyclopamine \| -0.0179 \| 0.900221 \| \| CXCL11 \| Cytarabine \| 0.04078 \| 0.434238 \| \| CXCL11 \| DMOG \| -0.01754 \| 0.743855 \| \| CXCL11 \| Dabrafenib \| 0.029654 \| 0.525854 \| \| CXCL11 \| Dasatinib \| -0.0471 \| 0.506897 \| \| CXCL11 \| Docetaxel \| -0.05901 \| 0.12231 \| \| CXCL11 \| Doxorubicin \| -0.03505 \| 0.602213 \| \| CXCL11 \| EHT 1864 \| 0.026987 \| 0.719509 \| \| CXCL11 \| EKB-569 \| -0.04902 \| 0.23254 \| \| CXCL11 \| EX-527 \| -0.01976 \| 0.87507 \| \| CXCL11 \| Elesclomol \| 0.008974 \| 0.86683 \| \| CXCL11 \| Embelin \| -0.0179 \| 0.805807 \| \| CXCL11 \| Epothilone B \| -0.05184 \| 0.304619 \| \| CXCL11 \| Erlotinib \| 0.031033 \| 0.697567 \| \| CXCL11 \| Etoposide \| -0.05051 \| 0.284749 \| \| CXCL11 \| FH535 \| 0.041939 \| 0.397049 \| \| CXCL11 \| FK866 \| 0.012977 \| 0.749581 \| \| CXCL11 \| FMK \| 0.003447 \| 0.970638 \| \| CXCL11 \| FR-180204 \| 0.00582 \| 0.937813 \| \| CXCL11 \| FTI-277 \| 0.02078 \| 0.65076 \| \| CXCL11 \| Foretinib \| -0.02759 \| 0.544987 \| \| CXCL11 \| GDC0449 \| -0.01397 \| 0.914269 \| \| CXCL11 \| GDC0941 \| -0.02945 \| 0.636054 \| \| CXCL11 \| GNF-2 \| -0.10522 \| 0.532983 \| \| CXCL11 \| GSK-650394 \| -0.02353 \| 0.786438 \| \| CXCL11 \| GSK1070916 \| -0.03122 \| 0.427247 \| \| CXCL11 \| GSK1904529A \| -0.00818 \| 0.885888 \| \| CXCL11 \| GSK2126458 \| -0.06981 \| 0.063436 \| \| CXCL11 \| GSK269962A \| 0.054314 \| 0.272609 \| \| CXCL11 \| GSK429286A \| 0.025844 \| 0.573039 \| \| CXCL11 \| GSK690693 \| -0.0417 \| 0.28113 \| \| CXCL11 \| GW 441756 \| 0.061252 \| 0.678263 \| \| CXCL11 \| GW-2580 \| 0.010494 \| 0.992332 \| \| CXCL11 \| GW843682X \| 0.034005 \| 0.739938 \| \| CXCL11 \| Gefitinib \| -0.06237 \| 0.132247 \| \| CXCL11 \| Gemcitabine \| -0.01498 \| 0.797647 \| \| CXCL11 \| Genentech Cpd 10 \| -0.04389 \| 0.254054 \| \| CXCL11 \| HG-5-113-01 \| -0.0224 \| 0.793669 \| \| CXCL11 \| HG-5-88-01 \| -0.05264 \| 0.719667 \| \| CXCL11 \| HG-6-64-1 \| -0.01428 \| 0.809087 \| \| CXCL11 \| I-BET-762 \| 0.000968 \| 0.980426 \| \| CXCL11 \| IOX2 \| -0.04996 \| 0.378193 \| \| CXCL11 \| IPA-3 \| -0.01441 \| 0.750137 \| \| CXCL11 \| Imatinib \| -0.11564 \| 0.250177 \| \| CXCL11 \| Ispinesib Mesylate \| -0.09289 \| 0.01044 \| \| CXCL11 \| JNJ-26854165 \| -0.01077 \| 0.869248 \| \| CXCL11 \| JNK Inhibitor VIII \| 0.012421 \| 0.800121 \| \| CXCL11 \| JNK-9L \| -0.05859 \| 0.269665 \| \| CXCL11 \| JQ1 \| -0.06078 \| 0.147723 \| \| CXCL11 \| JQ12 \| -0.00701 \| 0.919821 \| \| CXCL11 \| JW-7-24-1 \| -0.04892 \| 0.186859 \| \| CXCL11 \| JW-7-52-1 \| 0.024228 \| 0.8443 \| \| CXCL11 \| KIN001-055 \| -0.0195 \| 0.805295 \| \| CXCL11 \| KIN001-102 \| -0.05134 \| 0.162456 \| \| CXCL11 \| KIN001-135 \| 0.048781 \| 0.655478 \| \| CXCL11 \| KIN001-236 \| -0.00268 \| 0.952786 \| \| CXCL11 \| KIN001-244 \| -0.02014 \| 0.620917 \| \| CXCL11 \| KIN001-260 \| 0.002254 \| 0.958223 \| \| CXCL11 \| KIN001-266 \| -0.03447 \| 0.494952 \| \| CXCL11 \| KIN001-270 \| -0.01449 \| 0.751372 \| \| CXCL11 \| KU-55933 \| -0.00765 \| 0.931943 \| \| CXCL11 \| LAQ824 \| -0.01476 \| 0.74369 \| \| CXCL11 \| LFM-A13 \| 0.019727 \| 0.737924 \| \| CXCL11 \| LY317615 \| -0.02295 \| 0.638193 \| \| CXCL11 \| Lapatinib \| 0.036226 \| 0.594136 \| \| CXCL11 \| Lenalidomide \| 0.02481 \| 0.783395 \| \| CXCL11 \| Linifanib \| 0.010443 \| 0.875606 \| \| CXCL11 \| Lisitinib \| 0.004605 \| 0.962507 \| \| CXCL11 \| MG-132 \| 0.041607 \| 0.673163 \| \| CXCL11 \| MK-2206 \| 0.015355 \| 0.803113 \| \| CXCL11 \| MLN4924 \| 0.020137 \| 0.756977 \| \| CXCL11 \| MP470 \| 0.009464 \| 0.854717 \| \| CXCL11 \| MPS-1-IN-1 \| -0.03766 \| 0.342662 \| \| CXCL11 \| MS-275 \| 0.05365 \| 0.535733 \| \| CXCL11 \| Masitinib \| -0.08814 \| 0.017235 \| \| CXCL11 \| Methotrexate \| -0.03092 \| 0.435001 \| \| CXCL11 \| Midostaurin \| -0.01396 \| 0.78957 \| \| CXCL11 \| Mitomycin C \| -0.02554 \| 0.65872 \| \| CXCL11 \| NG-25 \| -0.04934 \| 0.186218 \| \| CXCL11 \| NPK76-II-72-1 \| -0.01959 \| 0.610594 \| \| CXCL11 \| NSC-207895 \| 0.01041 \| 0.852925 \| \| CXCL11 \| NSC-87877 \| -0.03054 \| 0.61137 \| \| CXCL11 \| NU-7441 \| -0.01683 \| 0.859611 \| \| CXCL11 \| Navitoclax \| 0.035964 \| 0.363036 \| \| CXCL11 \| Nilotinib \| -0.09262 \| 0.040775 \| \| CXCL11 \| Nutlin-3a (-) \| 0.046092 \| 0.344118 \| \| CXCL11 \| OSI-027 \| -0.05432 \| 0.143582 \| \| CXCL11 \| OSI-930 \| -0.04451 \| 0.267677 \| \| CXCL11 \| OSU-03012 \| -0.0426 \| 0.400622 \| \| CXCL11 \| Obatoclax Mesylate \| -0.04657 \| 0.331441 \| \| CXCL11 \| Olaparib \| -0.01792 \| 0.718446 \| \| CXCL11 \| PAC-1 \| -0.00169 \| 0.975576 \| \| CXCL11 \| PD-0325901 \| -0.01317 \| 0.772113 \| \| CXCL11 \| PD-0332991 \| -0.01591 \| 0.817816 \| \| CXCL11 \| PD-173074 \| 0.071573 \| 0.515975 \| \| CXCL11 \| PF-4708671 \| -0.0113 \| 0.935721 \| \| CXCL11 \| PF-562271 \| -0.0305 \| 0.616374 \| \| CXCL11 \| PFI-1 \| -0.01301 \| 0.82465 \| \| CXCL11 \| PHA-665752 \| 0.054225 \| 0.713801 \| \| CXCL11 \| PHA-793887 \| -0.05117 \| 0.159219 \| \| CXCL11 \| PI-103 \| -0.05817 \| 0.115429 \| \| CXCL11 \| PIK-93 \| -0.02606 \| 0.494296 \| \| CXCL11 \| PLX4720 \| 0.013037 \| 0.78518 \| \| CXCL11 \| Paclitaxel \| -0.03163 \| 0.807731 \| \| CXCL11 \| Parthenolide \| 0.041701 \| 0.710944 \| \| CXCL11 \| Pazopanib \| 0.033836 \| 0.565554 \| \| CXCL11 \| Phenformin \| -0.04146 \| 0.285128 \| \| CXCL11 \| Pyrimethamine \| -0.02093 \| 0.882867 \| \| CXCL11 \| QL-VIII-58 \| 0.008217 \| 0.939819 \| \| CXCL11 \| QL-X-138 \| -0.0368 \| 0.342324 \| \| CXCL11 \| QL-XI-92 \| -0.06326 \| 0.085932 \| \| CXCL11 \| QL-XII-47 \| -0.03563 \| 0.398405 \| \| CXCL11 \| QL-XII-61 \| 0.013099 \| 0.878748 \| \| CXCL11 \| QS11 \| -0.02417 \| 0.735762 \| \| CXCL11 \| RDEA119 \| -0.02819 \| 0.482821 \| \| CXCL11 \| RO-3306 \| -0.00826 \| 0.866257 \| \| CXCL11 \| Rapamycin \| 0.039592 \| 0.769755 \| \| CXCL11 \| Roscovitine \| 0.023618 \| 0.913043 \| \| CXCL11 \| Ruxolitinib \| 0.003663 \| 0.957776 \| \| CXCL11 \| S-Trityl-L-cysteine \| -0.01256 \| 0.907569 \| \| CXCL11 \| SB 216763 \| -8E-06 \| 0.999825 \| \| CXCL11 \| SB 505124 \| 0.051899 \| 0.460249 \| \| CXCL11 \| SB52334 \| 0.031678 \| 0.538857 \| \| CXCL11 \| SB590885 \| 0.020854 \| 0.705936 \| \| CXCL11 \| SGC0946 \| 0.020391 \| 0.744889 \| \| CXCL11 \| SL 0101-1 \| -0.01169 \| 0.905224 \| \| CXCL11 \| SN-38 \| -0.02354 \| 0.634825 \| \| CXCL11 \| SNX-2112 \| -0.08487 \| 0.01964 \| \| CXCL11 \| STF-62247 \| -0.02377 \| 0.595626 \| \| CXCL11 \| Salubrinal \| 0.047255 \| 0.599895 \| \| CXCL11 \| Saracatinib \| -0.05001 \| 0.567093 \| \| CXCL11 \| Shikonin \| -0.07527 \| 0.120751 \| \| CXCL11 \| Sorafenib \| 0.028093 \| 0.828216 \| \| CXCL11 \| Sunitinib \| 0.012668 \| 0.897882 \| \| CXCL11 \| T0901317 \| -0.0134 \| 0.780062 \| \| CXCL11 \| TAE684 \| -0.0167 \| 0.915541 \| \| CXCL11 \| TAK-715 \| -0.02954 \| 0.449412 \| \| CXCL11 \| TG101348 \| -0.03697 \| 0.328466 \| \| CXCL11 \| TGX221 \| 0.04398 \| 0.523138 \| \| CXCL11 \| THZ-2-102-1 \| -0.07598 \| 0.036994 \| \| CXCL11 \| THZ-2-49 \| -0.00862 \| 0.841046 \| \| CXCL11 \| TL-1-85 \| -0.04878 \| 0.194221 \| \| CXCL11 \| TL-2-105 \| -0.04353 \| 0.261613 \| \| CXCL11 \| TPCA-1 \| -0.02357 \| 0.540318 \| \| CXCL11 \| TW 37 \| 0.039247 \| 0.41398 \| \| CXCL11 \| Talazoparib \| -0.00625 \| 0.91073 \| \| CXCL11 \| Tamoxifen \| -0.04424 \| 0.691744 \| \| CXCL11 \| Temozolomide \| -0.00767 \| 0.938332 \| \| CXCL11 \| Temsirolimus \| 0.017941 \| 0.759873 \| \| CXCL11 \| Thapsigargin \| 0.005884 \| 0.950033 \| \| CXCL11 \| Tipifarnib \| 0.033728 \| 0.632854 \| \| CXCL11 \| Tivozanib \| -0.012 \| 0.874844 \| \| CXCL11 \| Trametinib \| -0.02778 \| 0.491181 \| \| CXCL11 \| Tubastatin A \| 0.007567 \| 0.853437 \| \| CXCL11 \| UNC0638 \| 0.003935 \| 0.923358 \| \| CXCL11 \| UNC1215 \| 0.006681 \| 0.917606 \| \| CXCL11 \| VNLG/124 \| -0.00487 \| 0.92497 \| \| CXCL11 \| VX-11e \| -0.0063 \| 0.923417 \| \| CXCL11 \| VX-680 \| -0.04337 \| 0.67423 \| \| CXCL11 \| VX-702 \| 0.018597 \| 0.940776 \| \| CXCL11 \| Veliparib \| 0.039088 \| 0.576719 \| \| CXCL11 \| Vinblastine \| -0.01563 \| 0.76374 \| \| CXCL11 \| Vinorelbine \| -0.04955 \| 0.360461 \| \| CXCL11 \| Vorinostat \| -0.0077 \| 0.851353 \| \| CXCL11 \| WH-4-023 \| -0.03922 \| 0.647394 \| \| CXCL11 \| WZ-1-84 \| 0.016228 \| 0.863988 \| \| CXCL11 \| WZ3105 \| -0.04602 \| 0.214107 \| \| CXCL11 \| XAV939 \| -0.02798 \| 0.502921 \| \| CXCL11 \| XL-184 \| -0.02127 \| 0.68994 \| \| CXCL11 \| XMD11-85h \| -0.04517 \| 0.802602 \| \| CXCL11 \| XMD13-2 \| -0.07016 \| 0.053441 \| \| CXCL11 \| XMD14-99 \| -0.0467 \| 0.234787 \| \| CXCL11 \| XMD15-27 \| -0.01551 \| 0.781695 \| \| CXCL11 \| XMD8-85 \| -0.01194 \| 0.912089 \| \| CXCL11 \| XMD8-92 \| -0.01856 \| 0.896849 \| \| CXCL11 \| Y-39983 \| -0.00469 \| 0.916912 \| \| CXCL11 \| YK 4-279 \| -0.00771 \| 0.916547 \| \| CXCL11 \| YM155 \| -0.03484 \| 0.526875 \| \| CXCL11 \| YM201636 \| -0.02488 \| 0.534608 \| \| CXCL11 \| Z-LLNle-CHO \| 0.009576 \| 0.919544 \| \| CXCL11 \| ZG-10 \| 0.03663 \| 0.615885 \| \| CXCL11 \| ZM-447439 \| -0.04336 \| 0.3657 \| \| CXCL11 \| ZSTK474 \| -0.04611 \| 0.220951 \| \| CXCL11 \| Zibotentan \| 0.038462 \| 0.874873 \| \| CXCL11 \| piperlongumine \| 0.016297 \| 0.750166 \| \| CXCL11 \| rTRAIL \| -0.01554 \| 0.867285 \| \| CXCL11 \| selumetinib \| -0.02948 \| 0.462974 \| \| CXCL9 \| (5Z)-7-Oxozeaenol \| 0.016901 \| 0.727978 \| \| CXCL9 \| 17-AAG \| 0.182608 \| 3.83E-07 \| \| CXCL9 \| 5-Fluorouracil \| -0.17868 \| 3.31E-07 \| \| CXCL9 \| 681640 \| 0.043444 \| 0.478258 \| \| CXCL9 \| A-443654 \| 0.038429 \| 0.847939 \| \| CXCL9 \| A-770041 \| 0.000743 \| 0.994968 \| \| CXCL9 \| AC220 \| -0.01074 \| 0.864993 \| \| CXCL9 \| AG-014699 \| 0.053846 \| 0.206906 \| \| CXCL9 \| AICAR \| -0.15664 \| 3.23E-05 \| \| CXCL9 \| AKT inhibitor VIII \| 0.054822 \| 0.224347 \| \| CXCL9 \| AMG-706 \| 0.054885 \| 0.337284 \| \| CXCL9 \| AP-24534 \| -0.06576 \| 0.108275 \| \| CXCL9 \| AR-42 \| -0.16271 \| 3.09E-06 \| \| CXCL9 \| AS601245 \| 0.063819 \| 0.210665 \| \| CXCL9 \| AS605240 \| -0.06008 \| 0.154247 \| \| CXCL9 \| AT-7519 \| -0.12608 \| 0.000334 \| \| CXCL9 \| ATRA \| -0.02832 \| 0.599826 \| \| CXCL9 \| AUY922 \| -0.00365 \| 0.960907 \| \| CXCL9 \| AZ628 \| 0.063411 \| 0.407908 \| \| CXCL9 \| AZD6482 \| 0.001936 \| 0.976234 \| \| CXCL9 \| AZD7762 \| -0.06302 \| 0.120229 \| \| CXCL9 \| AZD8055 \| -0.13548 \| 0.000352 \| \| CXCL9 \| Afatinib \| 0.058596 \| 0.119127 \| \| CXCL9 \| Axitinib \| 0.004858 \| 0.947189 \| \| CXCL9 \| BAY 61-3606 \| -0.10903 \| 0.005165 \| \| CXCL9 \| BEZ235 \| 0.013575 \| 0.818762 \| \| CXCL9 \| BHG712 \| -0.11781 \| 0.000903 \| \| CXCL9 \| BI-2536 \| 0.076373 \| 0.436623 \| \| CXCL9 \| BIRB 0796 \| 0.094272 \| 0.044596 \| \| CXCL9 \| BIX02189 \| -0.10577 \| 0.003241 \| \| CXCL9 \| BMS-509744 \| 0.111987 \| 0.185229 \| \| CXCL9 \| BMS-536924 \| 0.040469 \| 0.559471 \| \| CXCL9 \| BMS-708163 \| 0.081773 \| 0.037142 \| \| CXCL9 \| BMS-754807 \| 0.080338 \| 0.104784 \| \| CXCL9 \| BMS345541 \| -0.15067 \| 1.6E-05 \| \| CXCL9 \| BX-795 \| 0.005329 \| 0.93192 \| \| CXCL9 \| BX-912 \| -0.07427 \| 0.038654 \| \| CXCL9 \| Belinostat \| -0.14294 \| 7.02E-05 \| \| CXCL9 \| Bexarotene \| 0.031013 \| 0.702971 \| \| CXCL9 \| Bicalutamide \| 0.057719 \| 0.188972 \| \| CXCL9 \| Bleomycin \| -0.03842 \| 0.506091 \| \| CXCL9 \| Bleomycin (50 uM) \| 0.088701 \| 0.013932 \| \| CXCL9 \| Bortezomib \| 0.053125 \| 0.526921 \| \| CXCL9 \| Bosutinib \| -0.04091 \| 0.458417 \| \| CXCL9 \| Bryostatin 1 \| 0.079185 \| 0.096025 \| \| CXCL9 \| CAL-101 \| -0.1359 \| 0.00016 \| \| CXCL9 \| CAY10603 \| -0.1562 \| 7.49E-06 \| \| CXCL9 \| CCT007093 \| 0.047759 \| 0.280565 \| \| CXCL9 \| CCT018159 \| -0.00646 \| 0.926805 \| \| CXCL9 \| CEP-701 \| -0.08315 \| 0.036726 \| \| CXCL9 \| CGP-082996 \| 0.007474 \| 0.956607 \| \| CXCL9 \| CGP-60474 \| -0.03911 \| 0.683555 \| \| CXCL9 \| CH5424802 \| -0.0243 \| 0.720072 \| \| CXCL9 \| CHIR-99021 \| 0.070438 \| 0.06359 \| \| CXCL9 \| CI-1040 \| 0.056074 \| 0.201156 \| \| CXCL9 \| CMK \| -0.13509 \| 0.14729 \| \| CXCL9 \| CP466722 \| -0.10168 \| 0.004246 \| \| CXCL9 \| CP724714 \| 0.047992 \| 0.366775 \| \| CXCL9 \| CUDC-101 \| -0.13951 \| 9.4E-05 \| \| CXCL9 \| CX-5461 \| -0.11921 \| 0.000869 \| \| CXCL9 \| Camptothecin \| -0.06713 \| 0.117076 \| \| CXCL9 \| Cetuximab \| 0.06391 \| 0.116778 \| \| CXCL9 \| Cisplatin \| 0.045965 \| 0.358868 \| \| CXCL9 \| Crizotinib \| 0.039478 \| 0.806035 \| \| CXCL9 \| Cyclopamine \| -0.01813 \| 0.899019 \| \| CXCL9 \| Cytarabine \| -0.02323 \| 0.687155 \| \| CXCL9 \| DMOG \| -0.06907 \| 0.111258 \| \| CXCL9 \| Dabrafenib \| 0.008869 \| 0.863253 \| \| CXCL9 \| Dasatinib \| 0.073435 \| 0.278822 \| \| CXCL9 \| Docetaxel \| 0.125226 \| 0.000687 \| \| CXCL9 \| Doxorubicin \| -0.04642 \| 0.462135 \| \| CXCL9 \| EHT 1864 \| 0.010466 \| 0.909991 \| \| CXCL9 \| EKB-569 \| -0.0942 \| 0.014325 \| \| CXCL9 \| EX-527 \| 0.03594 \| 0.744622 \| \| CXCL9 \| Elesclomol \| 0.084287 \| 0.046649 \| \| CXCL9 \| Embelin \| -0.0025 \| 0.978248 \| \| CXCL9 \| Epothilone B \| 0.046354 \| 0.367167 \| \| CXCL9 \| Erlotinib \| 0.061484 \| 0.408337 \| \| CXCL9 \| Etoposide \| -0.07183 \| 0.105624 \| \| CXCL9 \| FH535 \| 0.044097 \| 0.36969 \| \| CXCL9 \| FK866 \| -0.13107 \| 0.00022 \| \| CXCL9 \| FMK \| 0.009922 \| 0.911288 \| \| CXCL9 \| FR-180204 \| -0.0279 \| 0.631232 \| \| CXCL9 \| FTI-277 \| 0.051983 \| 0.218144 \| \| CXCL9 \| Foretinib \| -0.02933 \| 0.516548 \| \| CXCL9 \| GDC0449 \| 0.004982 \| 0.974615 \| \| CXCL9 \| GDC0941 \| 0.008752 \| 0.904025 \| \| CXCL9 \| GNF-2 \| 0.025955 \| 0.928795 \| \| CXCL9 \| GSK-650394 \| 0.041508 \| 0.572102 \| \| CXCL9 \| GSK1070916 \| -0.05158 \| 0.174944 \| \| CXCL9 \| GSK1904529A \| 0.09956 \| 0.019686 \| \| CXCL9 \| GSK2126458 \| -0.11816 \| 0.001128 \| \| CXCL9 \| GSK269962A \| 0.055466 \| 0.259555 \| \| CXCL9 \| GSK429286A \| 0.000222 \| 0.996878 \| \| CXCL9 \| GSK690693 \| -0.09518 \| 0.008932 \| \| CXCL9 \| GW 441756 \| 0.033441 \| 0.873489 \| \| CXCL9 \| GW-2580 \| 0.014965 \| 0.984347 \| \| CXCL9 \| GW843682X \| 0.040249 \| 0.684096 \| \| CXCL9 \| Gefitinib \| 0.009593 \| 0.839455 \| \| CXCL9 \| Gemcitabine \| -0.06262 \| 0.185549 \| \| CXCL9 \| Genentech Cpd 10 \| -0.06661 \| 0.074014 \| \| CXCL9 \| HG-5-113-01 \| -0.07476 \| 0.272732 \| \| CXCL9 \| HG-5-88-01 \| 0.030307 \| 0.869698 \| \| CXCL9 \| HG-6-64-1 \| -0.01781 \| 0.755489 \| \| CXCL9 \| I-BET-762 \| -0.17438 \| 3.4E-07 \| \| CXCL9 \| IOX2 \| 0.022685 \| 0.731572 \| \| CXCL9 \| IPA-3 \| -0.0938 \| 0.014713 \| \| CXCL9 \| Imatinib \| -0.00399 \| 0.986368 \| \| CXCL9 \| Ispinesib Mesylate \| -0.10984 \| 0.002235 \| \| CXCL9 \| JNJ-26854165 \| 0.028226 \| 0.618529 \| \| CXCL9 \| JNK Inhibitor VIII \| 0.085484 \| 0.038241 \| \| CXCL9 \| JNK-9L \| -0.04642 \| 0.408909 \| \| CXCL9 \| JQ1 \| -0.07034 \| 0.08935 \| \| CXCL9 \| JQ12 \| -0.04536 \| 0.406911 \| \| CXCL9 \| JW-7-24-1 \| -0.15275 \| 1.15E-05 \| \| CXCL9 \| JW-7-52-1 \| 0.043518 \| 0.690159 \| \| CXCL9 \| KIN001-055 \| -0.00152 \| 0.986617 \| \| CXCL9 \| KIN001-102 \| -0.14476 \| 3.13E-05 \| \| CXCL9 \| KIN001-135 \| -0.00639 \| 0.967876 \| \| CXCL9 \| KIN001-236 \| -0.07489 \| 0.043352 \| \| CXCL9 \| KIN001-244 \| -0.11399 \| 0.001512 \| \| CXCL9 \| KIN001-260 \| -0.11597 \| 0.001135 \| \| CXCL9 \| KIN001-266 \| -0.06811 \| 0.133918 \| \| CXCL9 \| KIN001-270 \| -0.07476 \| 0.053706 \| \| CXCL9 \| KU-55933 \| -0.0423 \| 0.49311 \| \| CXCL9 \| LAQ824 \| -0.0964 \| 0.012174 \| \| CXCL9 \| LFM-A13 \| 0.061663 \| 0.203469 \| \| CXCL9 \| LY317615 \| -0.05589 \| 0.197515 \| \| CXCL9 \| Lapatinib \| 0.118934 \| 0.053695 \| \| CXCL9 \| Lenalidomide \| 0.008991 \| 0.937552 \| \| CXCL9 \| Linifanib \| -0.00062 \| 0.993832 \| \| CXCL9 \| Lisitinib \| 0.039173 \| 0.556659 \| \| CXCL9 \| MG-132 \| 0.039962 \| 0.687355 \| \| CXCL9 \| MK-2206 \| -0.0426 \| 0.418178 \| \| CXCL9 \| MLN4924 \| 0.071026 \| 0.185777 \| \| CXCL9 \| MP470 \| -0.06702 \| 0.114764 \| \| CXCL9 \| MPS-1-IN-1 \| -0.07964 \| 0.033434 \| \| CXCL9 \| MS-275 \| 0.052084 \| 0.549614 \| \| CXCL9 \| Masitinib \| -0.09577 \| 0.009306 \| \| CXCL9 \| Methotrexate \| -0.18315 \| 3.07E-07 \| \| CXCL9 \| Midostaurin \| 0.087983 \| 0.035797 \| \| CXCL9 \| Mitomycin C \| -0.05004 \| 0.326121 \| \| CXCL9 \| NG-25 \| -0.13983 \| 6.57E-05 \| \| CXCL9 \| NPK76-II-72-1 \| -0.15094 \| 1.18E-05 \| \| CXCL9 \| NSC-207895 \| -0.06141 \| 0.157367 \| \| CXCL9 \| NSC-87877 \| 0.065744 \| 0.214979 \| \| CXCL9 \| NU-7441 \| -0.01285 \| 0.899986 \| \| CXCL9 \| Navitoclax \| -0.12627 \| 0.000562 \| \| CXCL9 \| Nilotinib \| -0.03024 \| 0.590903 \| \| CXCL9 \| Nutlin-3a (-) \| -0.07201 \| 0.109578 \| \| CXCL9 \| OSI-027 \| -0.17331 \| 6.91E-07 \| \| CXCL9 \| OSI-930 \| -0.08366 \| 0.027773 \| \| CXCL9 \| OSU-03012 \| -0.03179 \| 0.551174 \| \| CXCL9 \| Obatoclax Mesylate \| -0.04224 \| 0.385573 \| \| CXCL9 \| Olaparib \| 0.061218 \| 0.155737 \| \| CXCL9 \| PAC-1 \| -0.12783 \| 0.00104 \| \| CXCL9 \| PD-0325901 \| 0.106566 \| 0.005855 \| \| CXCL9 \| PD-0332991 \| 0.003795 \| 0.96082 \| \| CXCL9 \| PD-173074 \| 0.010616 \| 0.962767 \| \| CXCL9 \| PF-4708671 \| 0.034117 \| 0.755141 \| \| CXCL9 \| PF-562271 \| 0.042963 \| 0.447006 \| \| CXCL9 \| PFI-1 \| -0.06886 \| 0.128045 \| \| CXCL9 \| PHA-665752 \| 0.069534 \| 0.62043 \| \| CXCL9 \| PHA-793887 \| -0.16306 \| 2.13E-06 \| \| CXCL9 \| PI-103 \| -0.18443 \| 1.16E-07 \| \| CXCL9 \| PIK-93 \| -0.14584 \| 2.57E-05 \| \| CXCL9 \| PLX4720 \| 0.013158 \| 0.783245 \| \| CXCL9 \| Paclitaxel \| 0.025062 \| 0.858085 \| \| CXCL9 \| Parthenolide \| -0.10641 \| 0.254146 \| \| CXCL9 \| Pazopanib \| 0.050433 \| 0.353161 \| \| CXCL9 \| Phenformin \| -0.08261 \| 0.025307 \| \| CXCL9 \| Pyrimethamine \| 0.050125 \| 0.675209 \| \| CXCL9 \| QL-VIII-58 \| 0.013376 \| 0.897575 \| \| CXCL9 \| QL-X-138 \| -0.16175 \| 4.44E-06 \| \| CXCL9 \| QL-XI-92 \| -0.19094 \| 3.56E-08 \| \| CXCL9 \| QL-XII-47 \| -0.10697 \| 0.004854 \| \| CXCL9 \| QL-XII-61 \| -0.11354 \| 0.058219 \| \| CXCL9 \| QS11 \| -0.01384 \| 0.863741 \| \| CXCL9 \| RDEA119 \| 0.105933 \| 0.003045 \| \| CXCL9 \| RO-3306 \| 0.088701 \| 0.030308 \| \| CXCL9 \| Rapamycin \| -0.01886 \| 0.907564 \| \| CXCL9 \| Roscovitine \| 0.0067 \| 0.980084 \| \| CXCL9 \| Ruxolitinib \| -0.02744 \| 0.631692 \| \| CXCL9 \| S-Trityl-L-cysteine \| -0.02653 \| 0.786966 \| \| CXCL9 \| SB 216763 \| 0.093309 \| 0.04667 \| \| CXCL9 \| SB 505124 \| 0.052963 \| 0.450214 \| \| CXCL9 \| SB52334 \| -0.02602 \| 0.626648 \| \| CXCL9 \| SB590885 \| 0.011496 \| 0.841962 \| \| CXCL9 \| SGC0946 \| 0.039497 \| 0.47071 \| \| CXCL9 \| SL 0101-1 \| 0.013513 \| 0.886934 \| \| CXCL9 \| SN-38 \| -0.024 \| 0.627566 \| \| CXCL9 \| SNX-2112 \| -0.16683 \| 1.87E-06 \| \| CXCL9 \| STF-62247 \| -0.07837 \| 0.046631 \| \| CXCL9 \| Salubrinal \| -0.04106 \| 0.655989 \| \| CXCL9 \| Saracatinib \| 0.047223 \| 0.593954 \| \| CXCL9 \| Shikonin \| -0.03058 \| 0.595739 \| \| CXCL9 \| Sorafenib \| 0.044685 \| 0.708523 \| \| CXCL9 \| Sunitinib \| 0.004789 \| 0.965617 \| \| CXCL9 \| T0901317 \| -0.08029 \| 0.042326 \| \| CXCL9 \| TAE684 \| 0.099967 \| 0.2827 \| \| CXCL9 \| TAK-715 \| -0.1508 \| 1.78E-05 \| \| CXCL9 \| TG101348 \| -0.13262 \| 0.000154 \| \| CXCL9 \| TGX221 \| 0.063053 \| 0.343317 \| \| CXCL9 \| THZ-2-102-1 \| -0.15362 \| 1.25E-05 \| \| CXCL9 \| THZ-2-49 \| -0.08194 \| 0.025853 \| \| CXCL9 \| TL-1-85 \| -0.12552 \| 0.000383 \| \| CXCL9 \| TL-2-105 \| -0.07188 \| 0.053843 \| \| CXCL9 \| TPCA-1 \| -0.17568 \| 3.19E-07 \| \| CXCL9 \| TW 37 \| -0.01808 \| 0.739453 \| \| CXCL9 \| Talazoparib \| 0.013487 \| 0.799729 \| \| CXCL9 \| Tamoxifen \| -0.02273 \| 0.869066 \| \| CXCL9 \| Temozolomide \| -0.00082 \| 0.994337 \| \| CXCL9 \| Temsirolimus \| -0.02398 \| 0.669808 \| \| CXCL9 \| Thapsigargin \| 0.019881 \| 0.802335 \| \| CXCL9 \| Tipifarnib \| -0.00021 \| 0.997892 \| \| CXCL9 \| Tivozanib \| -0.03328 \| 0.592629 \| \| CXCL9 \| Trametinib \| 0.114314 \| 0.001623 \| \| CXCL9 \| Tubastatin A \| -0.12045 \| 0.000628 \| \| CXCL9 \| UNC0638 \| -0.11325 \| 0.001104 \| \| CXCL9 \| UNC1215 \| 0.055858 \| 0.256425 \| \| CXCL9 \| VNLG/124 \| -0.06545 \| 0.107307 \| \| CXCL9 \| VX-11e \| -0.00246 \| 0.969532 \| \| CXCL9 \| VX-680 \| 0.034079 \| 0.751461 \| \| CXCL9 \| VX-702 \| 0.009744 \| 0.977352 \| \| CXCL9 \| Veliparib \| 0.063398 \| 0.322108 \| \| CXCL9 \| Vinblastine \| -0.01905 \| 0.708485 \| \| CXCL9 \| Vinorelbine \| -0.01167 \| 0.868162 \| \| CXCL9 \| Vorinostat \| -0.13269 \| 0.000247 \| \| CXCL9 \| WH-4-023 \| 0.031846 \| 0.718893 \| \| CXCL9 \| WZ-1-84 \| 0.073495 \| 0.343484 \| \| CXCL9 \| WZ3105 \| -0.11487 \| 0.001099 \| \| CXCL9 \| XAV939 \| 0.063897 \| 0.101887 \| \| CXCL9 \| XL-184 \| -0.01047 \| 0.857027 \| \| CXCL9 \| XMD11-85h \| -0.07924 \| 0.586622 \| \| CXCL9 \| XMD13-2 \| -0.17347 \| 5.56E-07 \| \| CXCL9 \| XMD14-99 \| -0.088 \| 0.018044 \| \| CXCL9 \| XMD15-27 \| -0.0469 \| 0.32302 \| \| CXCL9 \| XMD8-85 \| -0.05766 \| 0.497446 \| \| CXCL9 \| XMD8-92 \| -0.08141 \| 0.396096 \| \| CXCL9 \| Y-39983 \| -0.0905 \| 0.014013 \| \| CXCL9 \| YK 4-279 \| 0.046185 \| 0.39382 \| \| CXCL9 \| YM155 \| -0.06594 \| 0.169247 \| \| CXCL9 \| YM201636 \| -0.11033 \| 0.002143 \| \| CXCL9 \| Z-LLNle-CHO \| -0.07483 \| 0.308505 \| \| CXCL9 \| ZG-10 \| 0.000681 \| 0.994357 \| \| CXCL9 \| ZM-447439 \| -0.01626 \| 0.766651 \| \| CXCL9 \| ZSTK474 \| -0.14387 \| 4.49E-05 \| \| CXCL9 \| Zibotentan \| -0.00049 \| 0.999485 \| \| CXCL9 \| piperlongumine \| 0.028656 \| 0.547243 \| \| CXCL9 \| rTRAIL \| 0.022677 \| 0.788437 \| \| CXCL9 \| selumetinib \| 0.099912 \| 0.005665 \| \| GZMA \| (5Z)-7-Oxozeaenol \| -0.04985 \| 0.234845 \| \| GZMA \| 17-AAG \| 0.13338 \| 0.000259 \| \| GZMA \| 5-Fluorouracil \| -0.23927 \| 3.87E-12 \| \| GZMA \| 681640 \| -0.00267 \| 0.973141 \| \| GZMA \| A-443654 \| -0.03228 \| 0.880166 \| \| GZMA \| A-770041 \| 0.032176 \| 0.731767 \| \| GZMA \| AC220 \| -0.04069 \| 0.408323 \| \| GZMA \| AG-014699 \| 0.027234 \| 0.555974 \| \| GZMA \| AICAR \| -0.13003 \| 0.000638 \| \| GZMA \| AKT inhibitor VIII \| 0.074237 \| 0.08736 \| \| GZMA \| AMG-706 \| 0.078775 \| 0.130767 \| \| GZMA \| AP-24534 \| -0.06498 \| 0.112782 \| \| GZMA \| AR-42 \| -0.20703 \| 1.9E-09 \| \| GZMA \| AS601245 \| 0.030539 \| 0.603437 \| \| GZMA \| AS605240 \| -0.18492 \| 6.79E-07 \| \| GZMA \| AT-7519 \| -0.21912 \| 1.48E-10 \| \| GZMA \| ATRA \| -0.06311 \| 0.179093 \| \| GZMA \| AUY922 \| -0.06254 \| 0.202138 \| \| GZMA \| AZ628 \| -0.02375 \| 0.789704 \| \| GZMA \| AZD6482 \| 0.01763 \| 0.758819 \| \| GZMA \| AZD7762 \| -0.1979 \| 9.66E-08 \| \| GZMA \| AZD8055 \| -0.19959 \| 7.27E-08 \| \| GZMA \| Afatinib \| 0.085377 \| 0.01959 \| \| GZMA \| Axitinib \| -0.06225 \| 0.210166 \| \| GZMA \| BAY 61-3606 \| -0.12342 \| 0.001373 \| \| GZMA \| BEZ235 \| -0.06429 \| 0.165638 \| \| GZMA \| BHG712 \| -0.15735 \| 6.75E-06 \| \| GZMA \| BI-2536 \| -0.10029 \| 0.276999 \| \| GZMA \| BIRB 0796 \| 0.084995 \| 0.073309 \| \| GZMA \| BIX02189 \| -0.18517 \| 1.06E-07 \| \| GZMA \| BMS-509744 \| 0.083869 \| 0.340678 \| \| GZMA \| BMS-536924 \| 0.046441 \| 0.481398 \| \| GZMA \| BMS-708163 \| 0.082137 \| 0.036264 \| \| GZMA \| BMS-754807 \| 0.066001 \| 0.199482 \| \| GZMA \| BMS345541 \| -0.23525 \| 5.79E-12 \| \| GZMA \| BX-795 \| -0.12752 \| 0.001601 \| \| GZMA \| BX-912 \| -0.22974 \| 1.26E-11 \| \| GZMA \| Belinostat \| -0.19259 \| 5.24E-08 \| \| GZMA \| Bexarotene \| 0.031642 \| 0.696398 \| \| GZMA \| Bicalutamide \| 0.045698 \| 0.312271 \| \| GZMA \| Bleomycin \| -0.06493 \| 0.206776 \| \| GZMA \| Bleomycin (50 uM) \| 0.033977 \| 0.374126 \| \| GZMA \| Bortezomib \| -0.02573 \| 0.789522 \| \| GZMA \| Bosutinib \| -0.11319 \| 0.00968 \| \| GZMA \| Bryostatin 1 \| 0.053661 \| 0.291996 \| \| GZMA \| CAL-101 \| -0.16163 \| 5.57E-06 \| \| GZMA \| CAY10603 \| -0.20882 \| 1.25E-09 \| \| GZMA \| CCT007093 \| 0.072638 \| 0.083677 \| \| GZMA \| CCT018159 \| -0.02385 \| 0.679569 \| \| GZMA \| CEP-701 \| -0.21153 \| 9.8E-09 \| \| GZMA \| CGP-082996 \| -0.00682 \| 0.960575 \| \| GZMA \| CGP-60474 \| -0.09894 \| 0.197692 \| \| GZMA \| CH5424802 \| -0.31734 \| 2.81E-20 \| \| GZMA \| CHIR-99021 \| 0.024899 \| 0.552066 \| \| GZMA \| CI-1040 \| -0.01859 \| 0.712858 \| \| GZMA \| CMK \| -0.07985 \| 0.45825 \| \| GZMA \| CP466722 \| -0.28738 \| 1.09E-17 \| \| GZMA \| CP724714 \| 0.045296 \| 0.400592 \| \| GZMA \| CUDC-101 \| -0.19261 \| 4.02E-08 \| \| GZMA \| CX-5461 \| -0.18132 \| 2.16E-07 \| \| GZMA \| Camptothecin \| -0.1965 \| 2.73E-07 \| \| GZMA \| Cetuximab \| 0.083858 \| 0.034606 \| \| GZMA \| Cisplatin \| -0.05319 \| 0.278766 \| \| GZMA \| Crizotinib \| -0.23728 \| 0.00031 \| \| GZMA \| Cyclopamine \| -0.12123 \| 0.164325 \| \| GZMA \| Cytarabine \| -0.13535 \| 0.001416 \| \| GZMA \| DMOG \| -0.1374 \| 0.000494 \| \| GZMA \| Dabrafenib \| -0.05979 \| 0.161225 \| \| GZMA \| Dasatinib \| 0.078324 \| 0.244203 \| \| GZMA \| Docetaxel \| 0.128884 \| 0.00047 \| \| GZMA \| Doxorubicin \| -0.07974 \| 0.143069 \| \| GZMA \| EHT 1864 \| -0.01424 \| 0.871015 \| \| GZMA \| EKB-569 \| -0.22982 \| 1.12E-10 \| \| GZMA \| EX-527 \| 0.015481 \| 0.906866 \| \| GZMA \| Elesclomol \| 0.006557 \| 0.903975 \| \| GZMA \| Embelin \| -0.03603 \| 0.562599 \| \| GZMA \| Epothilone B \| 0.008221 \| 0.89769 \| \| GZMA \| Erlotinib \| 0.082483 \| 0.257414 \| \| GZMA \| Etoposide \| -0.14187 \| 0.000497 \| \| GZMA \| FH535 \| 0.070471 \| 0.118965 \| \| GZMA \| FK866 \| -0.20032 \| 8.02E-09 \| \| GZMA \| FMK \| -0.16322 \| 0.000422 \| \| GZMA \| FR-180204 \| -0.01487 \| 0.819333 \| \| GZMA \| FTI-277 \| 0.055105 \| 0.189109 \| \| GZMA \| Foretinib \| -0.12956 \| 0.000513 \| \| GZMA \| GDC0449 \| -0.10693 \| 0.126889 \| \| GZMA \| GDC0941 \| 0.021265 \| 0.748087 \| \| GZMA \| GNF-2 \| 0.027201 \| 0.923389 \| \| GZMA \| GSK-650394 \| -0.00726 \| 0.943834 \| \| GZMA \| GSK1070916 \| -0.24595 \| 9.72E-13 \| \| GZMA \| GSK1904529A \| 0.114136 \| 0.006534 \| \| GZMA \| GSK2126458 \| -0.13016 \| 0.000308 \| \| GZMA \| GSK269962A \| -0.08097 \| 0.069853 \| \| GZMA \| GSK429286A \| -0.16107 \| 1.3E-05 \| \| GZMA \| GSK690693 \| -0.12184 \| 0.000668 \| \| GZMA \| GW 441756 \| 0.057273 \| 0.712272 \| \| GZMA \| GW-2580 \| 0.038746 \| 0.917913 \| \| GZMA \| GW843682X \| -0.12164 \| 0.112285 \| \| GZMA \| Gefitinib \| 0.072179 \| 0.078387 \| \| GZMA \| Gemcitabine \| -0.12526 \| 0.003288 \| \| GZMA \| Genentech Cpd 10 \| -0.22454 \| 7.14E-11 \| \| GZMA \| HG-5-113-01 \| -0.11516 \| 0.068086 \| \| GZMA \| HG-5-88-01 \| -0.11786 \| 0.208892 \| \| GZMA \| HG-6-64-1 \| -0.01922 \| 0.732736 \| \| GZMA \| I-BET-762 \| -0.22377 \| 3.64E-11 \| \| GZMA \| IOX2 \| 0.083883 \| 0.104841 \| \| GZMA \| IPA-3 \| -0.129 \| 0.0006 \| \| GZMA \| Imatinib \| 0.001264 \| 0.996395 \| \| GZMA \| Ispinesib Mesylate \| -0.19118 \| 3.77E-08 \| \| GZMA \| JNJ-26854165 \| -0.03737 \| 0.482545 \| \| GZMA \| JNK Inhibitor VIII \| 0.091256 \| 0.02604 \| \| GZMA \| JNK-9L \| -0.05695 \| 0.285104 \| \| GZMA \| JQ1 \| -0.0865 \| 0.031957 \| \| GZMA \| JQ12 \| -0.09668 \| 0.043947 \| \| GZMA \| JW-7-24-1 \| -0.21012 \| 8.44E-10 \| \| GZMA \| JW-7-52-1 \| -0.04731 \| 0.659014 \| \| GZMA \| KIN001-055 \| -0.00106 \| 0.989732 \| \| GZMA \| KIN001-102 \| -0.22516 \| 3.9E-11 \| \| GZMA \| KIN001-135 \| 0.112491 \| 0.202517 \| \| GZMA \| KIN001-236 \| -0.13425 \| 0.000172 \| \| GZMA \| KIN001-244 \| -0.19432 \| 2.69E-08 \| \| GZMA \| KIN001-260 \| -0.17372 \| 6.39E-07 \| \| GZMA \| KIN001-266 \| -0.04639 \| 0.333551 \| \| GZMA \| KIN001-270 \| -0.1189 \| 0.001445 \| \| GZMA \| KU-55933 \| -0.09675 \| 0.052205 \| \| GZMA \| LAQ824 \| -0.17244 \| 2.91E-06 \| \| GZMA \| LFM-A13 \| 0.051477 \| 0.304388 \| \| GZMA \| LY317615 \| -0.14307 \| 0.000226 \| \| GZMA \| Lapatinib \| 0.112914 \| 0.068741 \| \| GZMA \| Lenalidomide \| -0.07842 \| 0.192425 \| \| GZMA \| Linifanib \| -0.03338 \| 0.545548 \| \| GZMA \| Lisitinib \| 0.087066 \| 0.095032 \| \| GZMA \| MG-132 \| -0.02542 \| 0.818667 \| \| GZMA \| MK-2206 \| -0.13186 \| 0.003217 \| \| GZMA \| MLN4924 \| -0.03848 \| 0.516644 \| \| GZMA \| MP470 \| -0.03764 \| 0.402661 \| \| GZMA \| MPS-1-IN-1 \| -0.2443 \| 3.03E-12 \| \| GZMA \| MS-275 \| -0.27047 \| 0.000111 \| \| GZMA \| Masitinib \| -0.15819 \| 9.26E-06 \| \| GZMA \| Methotrexate \| -0.34584 \| 5.87E-24 \| \| GZMA \| Midostaurin \| 0.013908 \| 0.790405 \| \| GZMA \| Mitomycin C \| -0.13996 \| 0.001144 \| \| GZMA \| NG-25 \| -0.12846 \| 0.000261 \| \| GZMA \| NPK76-II-72-1 \| -0.2464 \| 2.27E-13 \| \| GZMA \| NSC-207895 \| -0.01775 \| 0.736789 \| \| GZMA \| NSC-87877 \| 0.079044 \| 0.12634 \| \| GZMA \| NU-7441 \| -0.07037 \| 0.261184 \| \| GZMA \| Navitoclax \| -0.22051 \| 5.45E-10 \| \| GZMA \| Nilotinib \| -0.09714 \| 0.03047 \| \| GZMA \| Nutlin-3a (-) \| -0.11802 \| 0.004822 \| \| GZMA \| OSI-027 \| -0.19174 \| 3.35E-08 \| \| GZMA \| OSI-930 \| -0.07333 \| 0.056408 \| \| GZMA \| OSU-03012 \| -0.11146 \| 0.009893 \| \| GZMA \| Obatoclax Mesylate \| -0.11008 \| 0.008529 \| \| GZMA \| Olaparib \| -0.02511 \| 0.597601 \| \| GZMA \| PAC-1 \| -0.18278 \| 1.32E-06 \| \| GZMA \| PD-0325901 \| 0.038023 \| 0.368803 \| \| GZMA \| PD-0332991 \| -0.17599 \| 3.67E-05 \| \| GZMA \| PD-173074 \| -0.05775 \| 0.651057 \| \| GZMA \| PF-4708671 \| -0.00436 \| 0.981021 \| \| GZMA \| PF-562271 \| 0.022803 \| 0.721187 \| \| GZMA \| PFI-1 \| -0.08463 \| 0.054064 \| \| GZMA \| PHA-665752 \| 0.038898 \| 0.805791 \| \| GZMA \| PHA-793887 \| -0.23956 \| 1.27E-12 \| \| GZMA \| PI-103 \| -0.15423 \| 1.12E-05 \| \| GZMA \| PIK-93 \| -0.21868 \| 1.22E-10 \| \| GZMA \| PLX4720 \| -0.01494 \| 0.751203 \| \| GZMA \| Paclitaxel \| -0.08045 \| 0.422627 \| \| GZMA \| Parthenolide \| -0.13368 \| 0.144684 \| \| GZMA \| Pazopanib \| 0.042805 \| 0.443654 \| \| GZMA \| Phenformin \| -0.17912 \| 4.53E-07 \| \| GZMA \| Pyrimethamine \| -0.14713 \| 0.130803 \| \| GZMA \| QL-VIII-58 \| -0.0288 \| 0.749152 \| \| GZMA \| QL-X-138 \| -0.18659 \| 9.32E-08 \| \| GZMA \| QL-XI-92 \| -0.19125 \| 3.38E-08 \| \| GZMA \| QL-XII-47 \| -0.16994 \| 3.63E-06 \| \| GZMA \| QL-XII-61 \| -0.09818 \| 0.108304 \| \| GZMA \| QS11 \| -0.03326 \| 0.61556 \| \| GZMA \| RDEA119 \| 0.037061 \| 0.34535 \| \| GZMA \| RO-3306 \| 0.052922 \| 0.213078 \| \| GZMA \| Rapamycin \| -0.0857 \| 0.41796 \| \| GZMA \| Roscovitine \| -0.09308 \| 0.52587 \| \| GZMA \| Ruxolitinib \| -0.07246 \| 0.116141 \| \| GZMA \| S-Trityl-L-cysteine \| -0.15781 \| 0.025597 \| \| GZMA \| SB 216763 \| 0.075645 \| 0.113674 \| \| GZMA \| SB 505124 \| 0.039312 \| 0.605842 \| \| GZMA \| SB52334 \| 0.061352 \| 0.170318 \| \| GZMA \| SB590885 \| 0.004315 \| 0.940995 \| \| GZMA \| SGC0946 \| 0.096872 \| 0.03155 \| \| GZMA \| SL 0101-1 \| 0.031022 \| 0.705501 \| \| GZMA \| SN-38 \| -0.15583 \| 4.04E-05 \| \| GZMA \| SNX-2112 \| -0.2254 \| 5.57E-11 \| \| GZMA \| STF-62247 \| -0.12394 \| 0.001172 \| \| GZMA \| Salubrinal \| -0.05458 \| 0.535028 \| \| GZMA \| Saracatinib \| 0.028134 \| 0.766038 \| \| GZMA \| Shikonin \| -0.0744 \| 0.126371 \| \| GZMA \| Sorafenib \| -0.01281 \| 0.92803 \| \| GZMA \| Sunitinib \| -0.14936 \| 0.020823 \| \| GZMA \| T0901317 \| -0.06893 \| 0.086016 \| \| GZMA \| TAE684 \| -0.19405 \| 0.007013 \| \| GZMA \| TAK-715 \| -0.21494 \| 5.16E-10 \| \| GZMA \| TG101348 \| -0.24169 \| 1.06E-12 \| \| GZMA \| TGX221 \| 0.089089 \| 0.165311 \| \| GZMA \| THZ-2-102-1 \| -0.20687 \| 2.33E-09 \| \| GZMA \| THZ-2-49 \| -0.13435 \| 0.000179 \| \| GZMA \| TL-1-85 \| -0.13808 \| 8.61E-05 \| \| GZMA \| TL-2-105 \| -0.12106 \| 0.000794 \| \| GZMA \| TPCA-1 \| -0.27153 \| 6.05E-16 \| \| GZMA \| TW 37 \| -0.03574 \| 0.465313 \| \| GZMA \| Talazoparib \| -0.07787 \| 0.067148 \| \| GZMA \| Tamoxifen \| -0.00647 \| 0.968705 \| \| GZMA \| Temozolomide \| -0.11103 \| 0.021521 \| \| GZMA \| Temsirolimus \| -0.13427 \| 0.001094 \| \| GZMA \| Thapsigargin \| 0.029083 \| 0.688079 \| \| GZMA \| Tipifarnib \| -0.01642 \| 0.844043 \| \| GZMA \| Tivozanib \| -0.01065 \| 0.890605 \| \| GZMA \| Trametinib \| 0.092583 \| 0.011914 \| \| GZMA \| Tubastatin A \| -0.17717 \| 2.99E-07 \| \| GZMA \| UNC0638 \| -0.16103 \| 2.22E-06 \| \| GZMA \| UNC1215 \| 0.079719 \| 0.084486 \| \| GZMA \| VNLG/124 \| -0.05278 \| 0.204945 \| \| GZMA \| VX-11e \| -0.07706 \| 0.079159 \| \| GZMA \| VX-680 \| -0.15836 \| 0.038824 \| \| GZMA \| VX-702 \| -0.04798 \| 0.801399 \| \| GZMA \| Veliparib \| 0.018843 \| 0.815997 \| \| GZMA \| Vinblastine \| -0.16574 \| 2.14E-05 \| \| GZMA \| Vinorelbine \| -0.02706 \| 0.658426 \| \| GZMA \| Vorinostat \| -0.26195 \| 8.77E-14 \| \| GZMA \| WH-4-023 \| 0.049383 \| 0.549643 \| \| GZMA \| WZ-1-84 \| 0.112746 \| 0.115562 \| \| GZMA \| WZ3105 \| -0.21207 \| 5.5E-10 \| \| GZMA \| XAV939 \| 0.10442 \| 0.005775 \| \| GZMA \| XL-184 \| -0.05097 \| 0.272807 \| \| GZMA \| XMD11-85h \| -0.17406 \| 0.050654 \| \| GZMA \| XMD13-2 \| -0.23435 \| 6.18E-12 \| \| GZMA \| XMD14-99 \| -0.31459 \| 1.54E-20 \| \| GZMA \| XMD15-27 \| -0.22038 \| 3.18E-09 \| \| GZMA \| XMD8-85 \| -0.09558 \| 0.210415 \| \| GZMA \| XMD8-92 \| -0.13996 \| 0.080263 \| \| GZMA \| Y-39983 \| -0.16002 \| 6.58E-06 \| \| GZMA \| YK 4-279 \| -0.12809 \| 0.004335 \| \| GZMA \| YM155 \| -0.06232 \| 0.199891 \| \| GZMA \| YM201636 \| -0.08635 \| 0.017944 \| \| GZMA \| Z-LLNle-CHO \| -0.10192 \| 0.141121 \| \| GZMA \| ZG-10 \| -0.11503 \| 0.054041 \| \| GZMA \| ZM-447439 \| -0.20078 \| 4.36E-07 \| \| GZMA \| ZSTK474 \| -0.1623 \| 3.66E-06 \| \| GZMA \| Zibotentan \| 0.032237 \| 0.912262 \| \| GZMA \| piperlongumine \| -0.06054 \| 0.155395 \| \| GZMA \| rTRAIL \| 0.045468 \| 0.531691 \| \| GZMA \| selumetinib \| 0.051406 \| 0.179194 \| \| HLA-DRA \| (5Z)-7-Oxozeaenol \| -0.06004 \| 0.142079 \| \| HLA-DRA \| 17-AAG \| 0.173329 \| 1.53E-06 \| \| HLA-DRA \| 5-Fluorouracil \| -0.24695 \| 6.96E-13 \| \| HLA-DRA \| 681640 \| 0.047906 \| 0.426451 \| \| HLA-DRA \| A-443654 \| -0.07027 \| 0.662956 \| \| HLA-DRA \| A-770041 \| -0.11989 \| 0.091596 \| \| HLA-DRA \| AC220 \| -0.07979 \| 0.06461 \| \| HLA-DRA \| AG-014699 \| 0.104961 \| 0.009319 \| \| HLA-DRA \| AICAR \| -0.23448 \| 1.55E-10 \| \| HLA-DRA \| AKT inhibitor VIII \| 0.12759 \| 0.001888 \| \| HLA-DRA \| AMG-706 \| 0.042132 \| 0.492351 \| \| HLA-DRA \| AP-24534 \| -0.15244 \| 5.27E-05 \| \| HLA-DRA \| AR-42 \| -0.19829 \| 9.48E-09 \| \| HLA-DRA \| AS601245 \| -0.0081 \| 0.911221 \| \| HLA-DRA \| AS605240 \| -0.16104 \| 2.08E-05 \| \| HLA-DRA \| AT-7519 \| -0.24856 \| 2.5E-13 \| \| HLA-DRA \| ATRA \| -0.18561 \| 2.44E-06 \| \| HLA-DRA \| AUY922 \| -0.08518 \| 0.065208 \| \| HLA-DRA \| AZ628 \| -0.00627 \| 0.950384 \| \| HLA-DRA \| AZD6482 \| -0.08208 \| 0.049319 \| \| HLA-DRA \| AZD7762 \| -0.19426 \| 1.72E-07 \| \| HLA-DRA \| AZD8055 \| -0.24288 \| 3.12E-11 \| \| HLA-DRA \| Afatinib \| 0.077926 \| 0.034281 \| \| HLA-DRA \| Axitinib \| 0.026076 \| 0.66355 \| \| HLA-DRA \| BAY 61-3606 \| -0.19564 \| 1.66E-07 \| \| HLA-DRA \| BEZ235 \| -0.02714 \| 0.615289 \| \| HLA-DRA \| BHG712 \| -0.2444 \| 7.98E-13 \| \| HLA-DRA \| BI-2536 \| -0.02852 \| 0.820138 \| \| HLA-DRA \| BIRB 0796 \| 0.065094 \| 0.186119 \| \| HLA-DRA \| BIX02189 \| -0.2351 \| 7.52E-12 \| \| HLA-DRA \| BMS-509744 \| 0.041877 \| 0.670805 \| \| HLA-DRA \| BMS-536924 \| -0.02655 \| 0.736187 \| \| HLA-DRA \| BMS-708163 \| 0.092212 \| 0.018237 \| \| HLA-DRA \| BMS-754807 \| 0.102323 \| 0.029716 \| \| HLA-DRA \| BMS345541 \| -0.13643 \| 0.000101 \| \| HLA-DRA \| BX-795 \| -0.05209 \| 0.254784 \| \| HLA-DRA \| BX-912 \| -0.19635 \| 9.58E-09 \| \| HLA-DRA \| Belinostat \| -0.18823 \| 1.07E-07 \| \| HLA-DRA \| Bexarotene \| 0.008761 \| 0.939275 \| \| HLA-DRA \| Bicalutamide \| 0.071447 \| 0.094358 \| \| HLA-DRA \| Bleomycin \| -0.03233 \| 0.590816 \| \| HLA-DRA \| Bleomycin (50 uM) \| 0.101956 \| 0.004436 \| \| HLA-DRA \| Bortezomib \| -0.11542 \| 0.099151 \| \| HLA-DRA \| Bosutinib \| -0.04596 \| 0.392138 \| \| HLA-DRA \| Bryostatin 1 \| 0.08094 \| 0.087908 \| \| HLA-DRA \| CAL-101 \| -0.26957 \| 2.97E-15 \| \| HLA-DRA \| CAY10603 \| -0.21531 \| 3.5E-10 \| \| HLA-DRA \| CCT007093 \| 0.069868 \| 0.097749 \| \| HLA-DRA \| CCT018159 \| 0.034168 \| 0.530547 \| \| HLA-DRA \| CEP-701 \| -0.1718 \| 4.58E-06 \| \| HLA-DRA \| CGP-082996 \| -0.11537 \| 0.14724 \| \| HLA-DRA \| CGP-60474 \| -0.19134 \| 0.004275 \| \| HLA-DRA \| CH5424802 \| -0.09355 \| 0.049665 \| \| HLA-DRA \| CHIR-99021 \| 0.012564 \| 0.775419 \| \| HLA-DRA \| CI-1040 \| -0.0321 \| 0.498086 \| \| HLA-DRA \| CMK \| -0.15781 \| 0.081635 \| \| HLA-DRA \| CP466722 \| -0.25079 \| 1.4E-13 \| \| HLA-DRA \| CP724714 \| 0.031187 \| 0.593161 \| \| HLA-DRA \| CUDC-101 \| -0.21053 \| 1.62E-09 \| \| HLA-DRA \| CX-5461 \| -0.15452 \| 1.18E-05 \| \| HLA-DRA \| Camptothecin \| -0.09807 \| 0.016061 \| \| HLA-DRA \| Cetuximab \| 0.131547 \| 0.000567 \| \| HLA-DRA \| Cisplatin \| 0.033196 \| 0.525055 \| \| HLA-DRA \| Crizotinib \| -0.06969 \| 0.599095 \| \| HLA-DRA \| Cyclopamine \| -0.13712 \| 0.103472 \| \| HLA-DRA \| Cytarabine \| 0.000148 \| 0.998761 \| \| HLA-DRA \| DMOG \| -0.19581 \| 2.35E-07 \| \| HLA-DRA \| Dabrafenib \| -0.07385 \| 0.075496 \| \| HLA-DRA \| Dasatinib \| -0.00969 \| 0.903691 \| \| HLA-DRA \| Docetaxel \| 0.152107 \| 3.3E-05 \| \| HLA-DRA \| Doxorubicin \| -0.04883 \| 0.431909 \| \| HLA-DRA \| EHT 1864 \| 0.10552 \| 0.043506 \| \| HLA-DRA \| EKB-569 \| -0.09736 \| 0.011084 \| \| HLA-DRA \| EX-527 \| 0.03562 \| 0.746083 \| \| HLA-DRA \| Elesclomol \| 0.103563 \| 0.012311 \| \| HLA-DRA \| Embelin \| -0.02463 \| 0.72085 \| \| HLA-DRA \| Epothilone B \| 0.050021 \| 0.324383 \| \| HLA-DRA \| Erlotinib \| 0.10601 \| 0.134648 \| \| HLA-DRA \| Etoposide \| -0.05841 \| 0.204416 \| \| HLA-DRA \| FH535 \| 0.049253 \| 0.306707 \| \| HLA-DRA \| FK866 \| -0.16789 \| 1.65E-06 \| \| HLA-DRA \| FMK \| -0.03506 \| 0.625179 \| \| HLA-DRA \| FR-180204 \| -0.04524 \| 0.386017 \| \| HLA-DRA \| FTI-277 \| 0.07035 \| 0.086395 \| \| HLA-DRA \| Foretinib \| -0.12652 \| 0.000715 \| \| HLA-DRA \| GDC0449 \| -0.10189 \| 0.149033 \| \| HLA-DRA \| GDC0941 \| -0.09033 \| 0.053278 \| \| HLA-DRA \| GNF-2 \| 0.061884 \| 0.782691 \| \| HLA-DRA \| GSK-650394 \| 0.045245 \| 0.52495 \| \| HLA-DRA \| GSK1070916 \| -0.12779 \| 0.000361 \| \| HLA-DRA \| GSK1904529A \| 0.126686 \| 0.002299 \| \| HLA-DRA \| GSK2126458 \| -0.21944 \| 3.05E-10 \| \| HLA-DRA \| GSK269962A \| -0.00114 \| 0.98705 \| \| HLA-DRA \| GSK429286A \| -0.06488 \| 0.111506 \| \| HLA-DRA \| GSK690693 \| -0.16125 \| 4.71E-06 \| \| HLA-DRA \| GW 441756 \| -0.02078 \| 0.936903 \| \| HLA-DRA \| GW-2580 \| -0.03398 \| 0.937849 \| \| HLA-DRA \| GW843682X \| -0.08037 \| 0.342701 \| \| HLA-DRA \| Gefitinib \| 0.046284 \| 0.275213 \| \| HLA-DRA \| Gemcitabine \| -0.08321 \| 0.066098 \| \| HLA-DRA \| Genentech Cpd 10 \| -0.12754 \| 0.000344 \| \| HLA-DRA \| HG-5-113-01 \| 0.0005 \| 0.996586 \| \| HLA-DRA \| HG-5-88-01 \| -0.00768 \| 0.975195 \| \| HLA-DRA \| HG-6-64-1 \| -0.09396 \| 0.027157 \| \| HLA-DRA \| I-BET-762 \| -0.32549 \| 5.4E-23 \| \| HLA-DRA \| IOX2 \| 0.057892 \| 0.295639 \| \| HLA-DRA \| IPA-3 \| -0.2123 \| 5.52E-09 \| \| HLA-DRA \| Imatinib \| 0.004445 \| 0.985495 \| \| HLA-DRA \| Ispinesib Mesylate \| -0.2057 \| 2.81E-09 \| \| HLA-DRA \| JNJ-26854165 \| 0.014912 \| 0.811633 \| \| HLA-DRA \| JNK Inhibitor VIII \| 0.125787 \| 0.001598 \| \| HLA-DRA \| JNK-9L \| -0.09489 \| 0.045839 \| \| HLA-DRA \| JQ1 \| -0.10004 \| 0.011949 \| \| HLA-DRA \| JQ12 \| -0.06636 \| 0.193831 \| \| HLA-DRA \| JW-7-24-1 \| -0.28103 \| 6.12E-17 \| \| HLA-DRA \| JW-7-52-1 \| -0.13654 \| 0.082363 \| \| HLA-DRA \| KIN001-055 \| -0.02327 \| 0.75897 \| \| HLA-DRA \| KIN001-102 \| -0.21219 \| 5.28E-10 \| \| HLA-DRA \| KIN001-135 \| 0.098451 \| 0.284687 \| \| HLA-DRA \| KIN001-236 \| -0.20233 \| 6.56E-09 \| \| HLA-DRA \| KIN001-244 \| -0.23577 \| 8.78E-12 \| \| HLA-DRA \| KIN001-260 \| -0.28019 \| 1.28E-16 \| \| HLA-DRA \| KIN001-266 \| -0.09547 \| 0.028416 \| \| HLA-DRA \| KIN001-270 \| -0.15514 \| 2.33E-05 \| \| HLA-DRA \| KU-55933 \| -0.1314 \| 0.004958 \| \| HLA-DRA \| LAQ824 \| -0.1263 \| 0.0008 \| \| HLA-DRA \| LFM-A13 \| 0.090574 \| 0.050036 \| \| HLA-DRA \| LY317615 \| -0.10012 \| 0.012991 \| \| HLA-DRA \| Lapatinib \| 0.144721 \| 0.016439 \| \| HLA-DRA \| Lenalidomide \| -0.04824 \| 0.499444 \| \| HLA-DRA \| Linifanib \| -0.09183 \| 0.036335 \| \| HLA-DRA \| Lisitinib \| 0.068792 \| 0.222399 \| \| HLA-DRA \| MG-132 \| -0.0681 \| 0.436081 \| \| HLA-DRA \| MK-2206 \| -0.05319 \| 0.294795 \| \| HLA-DRA \| MLN4924 \| 0.077331 \| 0.14394 \| \| HLA-DRA \| MP470 \| -0.02172 \| 0.646756 \| \| HLA-DRA \| MPS-1-IN-1 \| -0.16518 \| 3.77E-06 \| \| HLA-DRA \| MS-275 \| -0.12241 \| 0.091307 \| \| HLA-DRA \| Masitinib \| -0.1823 \| 2.5E-07 \| \| HLA-DRA \| Methotrexate \| -0.3165 \| 5.4E-20 \| \| HLA-DRA \| Midostaurin \| 0.075952 \| 0.07506 \| \| HLA-DRA \| Mitomycin C \| -0.04644 \| 0.367608 \| \| HLA-DRA \| NG-25 \| -0.26569 \| 3.87E-15 \| \| HLA-DRA \| NPK76-II-72-1 \| -0.1938 \| 1.24E-08 \| \| HLA-DRA \| NSC-207895 \| -0.02544 \| 0.611614 \| \| HLA-DRA \| NSC-87877 \| 0.096629 \| 0.056906 \| \| HLA-DRA \| NU-7441 \| -0.06559 \| 0.308125 \| \| HLA-DRA \| Navitoclax \| -0.15847 \| 1.18E-05 \| \| HLA-DRA \| Nilotinib \| -0.03901 \| 0.467806 \| \| HLA-DRA \| Nutlin-3a (-) \| -0.1658 \| 4.05E-05 \| \| HLA-DRA \| OSI-027 \| -0.24847 \| 3.9E-13 \| \| HLA-DRA \| OSI-930 \| -0.18955 \| 1.22E-07 \| \| HLA-DRA \| OSU-03012 \| -0.05855 \| 0.220513 \| \| HLA-DRA \| Obatoclax Mesylate \| -0.15131 \| 0.000181 \| \| HLA-DRA \| Olaparib \| 0.048683 \| 0.269322 \| \| HLA-DRA \| PAC-1 \| -0.16397 \| 1.7E-05 \| \| HLA-DRA \| PD-0325901 \| 0.088757 \| 0.023881 \| \| HLA-DRA \| PD-0332991 \| -0.00638 \| 0.934594 \| \| HLA-DRA \| PD-173074 \| 0.015359 \| 0.937386 \| \| HLA-DRA \| PF-4708671 \| 0.061472 \| 0.488111 \| \| HLA-DRA \| PF-562271 \| 0.05475 \| 0.312035 \| \| HLA-DRA \| PFI-1 \| -0.09265 \| 0.03223 \| \| HLA-DRA \| PHA-665752 \| 0.01824 \| 0.916613 \| \| HLA-DRA \| PHA-793887 \| -0.3003 \| 1.56E-19 \| \| HLA-DRA \| PI-103 \| -0.27758 \| 2.84E-16 \| \| HLA-DRA \| PIK-93 \| -0.30855 \| 1.46E-20 \| \| HLA-DRA \| PLX4720 \| -0.09501 \| 0.01455 \| \| HLA-DRA \| Paclitaxel \| -0.10405 \| 0.258004 \| \| HLA-DRA \| Parthenolide \| -0.13145 \| 0.151711 \| \| HLA-DRA \| Pazopanib \| 0.02842 \| 0.636984 \| \| HLA-DRA \| Phenformin \| -0.24849 \| 9.36E-13 \| \| HLA-DRA \| Pyrimethamine \| -0.03186 \| 0.803464 \| \| HLA-DRA \| QL-VIII-58 \| -0.0158 \| 0.875474 \| \| HLA-DRA \| QL-X-138 \| -0.23772 \| 5.41E-12 \| \| HLA-DRA \| QL-XI-92 \| -0.28237 \| 6.05E-17 \| \| HLA-DRA \| QL-XII-47 \| -0.15369 \| 3.18E-05 \| \| HLA-DRA \| QL-XII-61 \| -0.13951 \| 0.015893 \| \| HLA-DRA \| QS11 \| 0.004338 \| 0.960656 \| \| HLA-DRA \| RDEA119 \| 0.054884 \| 0.146713 \| \| HLA-DRA \| RO-3306 \| 0.095233 \| 0.019331 \| \| HLA-DRA \| Rapamycin \| -0.13202 \| 0.161136 \| \| HLA-DRA \| Roscovitine \| -0.0272 \| 0.898392 \| \| HLA-DRA \| Ruxolitinib \| -0.12337 \| 0.002959 \| \| HLA-DRA \| S-Trityl-L-cysteine \| -0.17964 \| 0.010035 \| \| HLA-DRA \| SB 216763 \| 0.083845 \| 0.076544 \| \| HLA-DRA \| SB 505124 \| 0.119099 \| 0.032167 \| \| HLA-DRA \| SB52334 \| 0.017216 \| 0.765441 \| \| HLA-DRA \| SB590885 \| -0.023 \| 0.67459 \| \| HLA-DRA \| SGC0946 \| 0.075922 \| 0.106283 \| \| HLA-DRA \| SL 0101-1 \| 0.000377 \| 0.997609 \| \| HLA-DRA \| SN-38 \| -0.06583 \| 0.120246 \| \| HLA-DRA \| SNX-2112 \| -0.27192 \| 1.29E-15 \| \| HLA-DRA \| STF-62247 \| -0.17128 \| 4.11E-06 \| \| HLA-DRA \| Salubrinal \| -0.18785 \| 0.009428 \| \| HLA-DRA \| Saracatinib \| 0.059173 \| 0.489983 \| \| HLA-DRA \| Shikonin \| -0.09819 \| 0.03384 \| \| HLA-DRA \| Sorafenib \| -0.05351 \| 0.63589 \| \| HLA-DRA \| Sunitinib \| -0.1675 \| 0.008302 \| \| HLA-DRA \| T0901317 \| -0.20994 \| 7.05E-09 \| \| HLA-DRA \| TAE684 \| 0.050299 \| 0.668863 \| \| HLA-DRA \| TAK-715 \| -0.17361 \| 6.59E-07 \| \| HLA-DRA \| TG101348 \| -0.25567 \| 4.09E-14 \| \| HLA-DRA \| TGX221 \| -0.0075 \| 0.925199 \| \| HLA-DRA \| THZ-2-102-1 \| -0.23375 \| 1.12E-11 \| \| HLA-DRA \| THZ-2-49 \| -0.24813 \| 7.92E-13 \| \| HLA-DRA \| TL-1-85 \| -0.25694 \| 3.69E-14 \| \| HLA-DRA \| TL-2-105 \| -0.11332 \| 0.001767 \| \| HLA-DRA \| TPCA-1 \| -0.32981 \| 1.66E-23 \| \| HLA-DRA \| TW 37 \| 0.014412 \| 0.796549 \| \| HLA-DRA \| Talazoparib \| 0.023036 \| 0.648289 \| \| HLA-DRA \| Tamoxifen \| -0.04662 \| 0.672111 \| \| HLA-DRA \| Temozolomide \| -0.1376 \| 0.002053 \| \| HLA-DRA \| Temsirolimus \| -0.121 \| 0.003726 \| \| HLA-DRA \| Thapsigargin \| 0.029272 \| 0.68628 \| \| HLA-DRA \| Tipifarnib \| -0.03739 \| 0.587436 \| \| HLA-DRA \| Tivozanib \| -0.03781 \| 0.528959 \| \| HLA-DRA \| Trametinib \| 0.094454 \| 0.010205 \| \| HLA-DRA \| Tubastatin A \| -0.24429 \| 6.59E-13 \| \| HLA-DRA \| UNC0638 \| -0.20778 \| 6E-10 \| \| HLA-DRA \| UNC1215 \| 0.056541 \| 0.249615 \| \| HLA-DRA \| VNLG/124 \| -0.15787 \| 2.03E-05 \| \| HLA-DRA \| VX-11e \| -0.05618 \| 0.224965 \| \| HLA-DRA \| VX-680 \| -0.09387 \| 0.276437 \| \| HLA-DRA \| VX-702 \| -0.06779 \| 0.652037 \| \| HLA-DRA \| Veliparib \| 0.034671 \| 0.627273 \| \| HLA-DRA \| Vinblastine \| -0.13606 \| 0.000614 \| \| HLA-DRA \| Vinorelbine \| -0.04173 \| 0.457593 \| \| HLA-DRA \| Vorinostat \| -0.16412 \| 4.9E-06 \| \| HLA-DRA \| WH-4-023 \| 0.004095 \| 0.96854 \| \| HLA-DRA \| WZ-1-84 \| 0.026313 \| 0.770826 \| \| HLA-DRA \| WZ3105 \| -0.17039 \| 8.11E-07 \| \| HLA-DRA \| XAV939 \| 0.111039 \| 0.003229 \| \| HLA-DRA \| XL-184 \| -0.11244 \| 0.005798 \| \| HLA-DRA \| XMD11-85h \| -0.00368 \| 0.988079 \| \| HLA-DRA \| XMD13-2 \| -0.25102 \| 1.43E-13 \| \| HLA-DRA \| XMD14-99 \| -0.22099 \| 3E-10 \| \| HLA-DRA \| XMD15-27 \| -0.17782 \| 4.43E-06 \| \| HLA-DRA \| XMD8-85 \| -0.16674 \| 0.013944 \| \| HLA-DRA \| XMD8-92 \| -0.09721 \| 0.276474 \| \| HLA-DRA \| Y-39983 \| -0.18817 \| 8.74E-08 \| \| HLA-DRA \| YK 4-279 \| 0.012759 \| 0.852233 \| \| HLA-DRA \| YM155 \| -0.1076 \| 0.014215 \| \| HLA-DRA \| YM201636 \| -0.18514 \| 1.14E-07 \| \| HLA-DRA \| Z-LLNle-CHO \| -0.24356 \| 4.26E-05 \| \| HLA-DRA \| ZG-10 \| -0.06166 \| 0.355469 \| \| HLA-DRA \| ZM-447439 \| -0.04143 \| 0.390454 \| \| HLA-DRA \| ZSTK474 \| -0.27136 \| 1.4E-15 \| \| HLA-DRA \| Zibotentan \| -0.01384 \| 0.974903 \| \| HLA-DRA \| piperlongumine \| 0.000771 \| 0.990704 \| \| HLA-DRA \| rTRAIL \| 0.055304 \| 0.425118 \| \| HLA-DRA \| selumetinib \| 0.03914 \| 0.318699 \| \| IDO1 \| (5Z)-7-Oxozeaenol \| 0.035086 \| 0.429008 \| \| IDO1 \| 17-AAG \| -0.10859 \| 0.003233 \| \| IDO1 \| 5-Fluorouracil \| 0.054462 \| 0.145338 \| \| IDO1 \| 681640 \| -0.06433 \| 0.264198 \| \| IDO1 \| A-443654 \| -0.06121 \| 0.711868 \| \| IDO1 \| A-770041 \| -0.08705 \| 0.254928 \| \| IDO1 \| AC220 \| 0.038868 \| 0.433865 \| \| IDO1 \| AG-014699 \| -0.06155 \| 0.143358 \| \| IDO1 \| AICAR \| -0.0138 \| 0.761193 \| \| IDO1 \| AKT inhibitor VIII \| -0.06979 \| 0.110462 \| \| IDO1 \| AMG-706 \| -0.02314 \| 0.743805 \| \| IDO1 \| AP-24534 \| 0.022518 \| 0.625551 \| \| IDO1 \| AR-42 \| 0.109355 \| 0.002086 \| \| IDO1 \| AS601245 \| -0.1382 \| 0.001549 \| \| IDO1 \| AS605240 \| 0.041253 \| 0.356415 \| \| IDO1 \| AT-7519 \| 0.031822 \| 0.402073 \| \| IDO1 \| ATRA \| 0.026307 \| 0.630668 \| \| IDO1 \| AUY922 \| -0.07751 \| 0.100017 \| \| IDO1 \| AZ628 \| -0.02506 \| 0.777211 \| \| IDO1 \| AZD6482 \| -0.08447 \| 0.041894 \| \| IDO1 \| AZD7762 \| 0.009947 \| 0.8357 \| \| IDO1 \| AZD8055 \| 0.036659 \| 0.37927 \| \| IDO1 \| Afatinib \| -0.07941 \| 0.030794 \| \| IDO1 \| Axitinib \| 0.04581 \| 0.385688 \| \| IDO1 \| BAY 61-3606 \| -0.00336 \| 0.948077 \| \| IDO1 \| BEZ235 \| -0.08281 \| 0.063879 \| \| IDO1 \| BHG712 \| 0.08565 \| 0.017927 \| \| IDO1 \| BI-2536 \| -0.02877 \| 0.818269 \| \| IDO1 \| BIRB 0796 \| -0.01707 \| 0.77148 \| \| IDO1 \| BIX02189 \| 0.050432 \| 0.181666 \| \| IDO1 \| BMS-509744 \| -0.05327 \| 0.577213 \| \| IDO1 \| BMS-536924 \| 0.010435 \| 0.915224 \| \| IDO1 \| BMS-708163 \| -0.06647 \| 0.095358 \| \| IDO1 \| BMS-754807 \| -0.05447 \| 0.306728 \| \| IDO1 \| BMS345541 \| 0.044745 \| 0.231687 \| \| IDO1 \| BX-795 \| -0.01508 \| 0.787219 \| \| IDO1 \| BX-912 \| 0.053917 \| 0.139915 \| \| IDO1 \| Belinostat \| 0.061271 \| 0.104949 \| \| IDO1 \| Bexarotene \| -0.05075 \| 0.456359 \| \| IDO1 \| Bicalutamide \| -0.08009 \| 0.057764 \| \| IDO1 \| Bleomycin \| -0.06407 \| 0.214222 \| \| IDO1 \| Bleomycin (50 uM) \| -0.08191 \| 0.023797 \| \| IDO1 \| Bortezomib \| -0.0297 \| 0.753607 \| \| IDO1 \| Bosutinib \| -0.02661 \| 0.659559 \| \| IDO1 \| Bryostatin 1 \| -0.05327 \| 0.296402 \| \| IDO1 \| CAL-101 \| 0.011737 \| 0.787878 \| \| IDO1 \| CAY10603 \| 0.064806 \| 0.07506 \| \| IDO1 \| CCT007093 \| -0.01622 \| 0.750513 \| \| IDO1 \| CCT018159 \| -0.05059 \| 0.317675 \| \| IDO1 \| CEP-701 \| 0.014761 \| 0.75179 \| \| IDO1 \| CGP-082996 \| -0.02472 \| 0.838044 \| \| IDO1 \| CGP-60474 \| -0.09376 \| 0.229281 \| \| IDO1 \| CH5424802 \| 0.012994 \| 0.865711 \| \| IDO1 \| CHIR-99021 \| -0.05722 \| 0.138454 \| \| IDO1 \| CI-1040 \| 0.013352 \| 0.796918 \| \| IDO1 \| CMK \| -0.00888 \| 0.953896 \| \| IDO1 \| CP466722 \| 0.042381 \| 0.257945 \| \| IDO1 \| CP724714 \| 0.022064 \| 0.729567 \| \| IDO1 \| CUDC-101 \| 0.06772 \| 0.068561 \| \| IDO1 \| CX-5461 \| 0.034655 \| 0.373848 \| \| IDO1 \| Camptothecin \| -0.0221 \| 0.662716 \| \| IDO1 \| Cetuximab \| 0.002073 \| 0.964601 \| \| IDO1 \| Cisplatin \| -0.03513 \| 0.499468 \| \| IDO1 \| Crizotinib \| 0.008869 \| 0.970406 \| \| IDO1 \| Cyclopamine \| 0.006538 \| 0.965275 \| \| IDO1 \| Cytarabine \| 0.025881 \| 0.648182 \| \| IDO1 \| DMOG \| -0.04591 \| 0.320106 \| \| IDO1 \| Dabrafenib \| 0.026407 \| 0.577481 \| \| IDO1 \| Dasatinib \| -0.12577 \| 0.046815 \| \| IDO1 \| Docetaxel \| -0.11225 \| 0.002441 \| \| IDO1 \| Doxorubicin \| -0.07271 \| 0.191537 \| \| IDO1 \| EHT 1864 \| 0.049056 \| 0.438283 \| \| IDO1 \| EKB-569 \| -0.01009 \| 0.833307 \| \| IDO1 \| EX-527 \| -0.02845 \| 0.812419 \| \| IDO1 \| Elesclomol \| -0.0345 \| 0.464972 \| \| IDO1 \| Embelin \| -0.09491 \| 0.050348 \| \| IDO1 \| Epothilone B \| -0.11998 \| 0.007014 \| \| IDO1 \| Erlotinib \| -0.12998 \| 0.058132 \| \| IDO1 \| Etoposide \| -0.03254 \| 0.528633 \| \| IDO1 \| FH535 \| 0.006471 \| 0.917072 \| \| IDO1 \| FK866 \| 0.090903 \| 0.012002 \| \| IDO1 \| FMK \| 0.006287 \| 0.945227 \| \| IDO1 \| FR-180204 \| -0.00238 \| 0.976583 \| \| IDO1 \| FTI-277 \| -0.04585 \| 0.283597 \| \| IDO1 \| Foretinib \| -0.00896 \| 0.858186 \| \| IDO1 \| GDC0449 \| -0.03664 \| 0.718442 \| \| IDO1 \| GDC0941 \| 0.005154 \| 0.945247 \| \| IDO1 \| GNF-2 \| -0.01883 \| 0.947893 \| \| IDO1 \| GSK-650394 \| -0.03316 \| 0.672634 \| \| IDO1 \| GSK1070916 \| 0.060379 \| 0.108086 \| \| IDO1 \| GSK1904529A \| -0.02799 \| 0.574445 \| \| IDO1 \| GSK2126458 \| -0.01551 \| 0.713284 \| \| IDO1 \| GSK269962A \| 0.008257 \| 0.902336 \| \| IDO1 \| GSK429286A \| 0.071492 \| 0.076078 \| \| IDO1 \| GSK690693 \| 0.059467 \| 0.113788 \| \| IDO1 \| GW 441756 \| 0.036026 \| 0.860969 \| \| IDO1 \| GW-2580 \| -0.01245 \| 0.989269 \| \| IDO1 \| GW843682X \| -0.02933 \| 0.779881 \| \| IDO1 \| Gefitinib \| -0.06248 \| 0.1315 \| \| IDO1 \| Gemcitabine \| -0.07601 \| 0.097498 \| \| IDO1 \| Genentech Cpd 10 \| 0.028721 \| 0.470669 \| \| IDO1 \| HG-5-113-01 \| 0.014549 \| 0.872187 \| \| IDO1 \| HG-5-88-01 \| -0.05046 \| 0.738547 \| \| IDO1 \| HG-6-64-1 \| -0.08549 \| 0.048266 \| \| IDO1 \| I-BET-762 \| 0.116263 \| 0.000822 \| \| IDO1 \| IOX2 \| -0.01672 \| 0.813905 \| \| IDO1 \| IPA-3 \| 0.012862 \| 0.778405 \| \| IDO1 \| Imatinib \| 0.027058 \| 0.881196 \| \| IDO1 \| Ispinesib Mesylate \| -0.008 \| 0.847832 \| \| IDO1 \| JNJ-26854165 \| -0.06476 \| 0.176368 \| \| IDO1 \| JNK Inhibitor VIII \| -0.06956 \| 0.097588 \| \| IDO1 \| JNK-9L \| -0.04858 \| 0.381932 \| \| IDO1 \| JQ1 \| 0.022731 \| 0.631293 \| \| IDO1 \| JQ12 \| 0.007647 \| 0.911539 \| \| IDO1 \| JW-7-24-1 \| 0.053292 \| 0.148421 \| \| IDO1 \| JW-7-52-1 \| -0.09697 \| 0.265963 \| \| IDO1 \| KIN001-055 \| -0.05437 \| 0.349587 \| \| IDO1 \| KIN001-102 \| 0.061157 \| 0.092159 \| \| IDO1 \| KIN001-135 \| -0.03256 \| 0.78542 \| \| IDO1 \| KIN001-236 \| 0.066072 \| 0.07749 \| \| IDO1 \| KIN001-244 \| 0.048229 \| 0.205749 \| \| IDO1 \| KIN001-260 \| 0.064223 \| 0.080897 \| \| IDO1 \| KIN001-266 \| 0.012993 \| 0.819265 \| \| IDO1 \| KIN001-270 \| 0.026878 \| 0.531807 \| \| IDO1 \| KU-55933 \| -0.02599 \| 0.713334 \| \| IDO1 \| LAQ824 \| 0.034558 \| 0.410746 \| \| IDO1 \| LFM-A13 \| -0.08426 \| 0.071375 \| \| IDO1 \| LY317615 \| -0.03701 \| 0.418239 \| \| IDO1 \| Lapatinib \| -0.11221 \| 0.070627 \| \| IDO1 \| Lenalidomide \| 0.003852 \| 0.973343 \| \| IDO1 \| Linifanib \| 0.013521 \| 0.8343 \| \| IDO1 \| Lisitinib \| 0.007711 \| 0.933143 \| \| IDO1 \| MG-132 \| -0.01098 \| 0.930074 \| \| IDO1 \| MK-2206 \| 0.078881 \| 0.100515 \| \| IDO1 \| MLN4924 \| -0.02521 \| 0.690701 \| \| IDO1 \| MP470 \| 0.092385 \| 0.026815 \| \| IDO1 \| MPS-1-IN-1 \| 0.052623 \| 0.17278 \| \| IDO1 \| MS-275 \| 0.09124 \| 0.23612 \| \| IDO1 \| Masitinib \| 0.043295 \| 0.269258 \| \| IDO1 \| Methotrexate \| 0.081642 \| 0.02832 \| \| IDO1 \| Midostaurin \| -0.09865 \| 0.017176 \| \| IDO1 \| Mitomycin C \| -0.07736 \| 0.097134 \| \| IDO1 \| NG-25 \| 0.079306 \| 0.028312 \| \| IDO1 \| NPK76-II-72-1 \| 0.078476 \| 0.027112 \| \| IDO1 \| NSC-207895 \| 0.078829 \| 0.059845 \| \| IDO1 \| NSC-87877 \| -0.05052 \| 0.358194 \| \| IDO1 \| NU-7441 \| -0.02346 \| 0.786254 \| \| IDO1 \| Navitoclax \| 0.086816 \| 0.019911 \| \| IDO1 \| Nilotinib \| 0.033927 \| 0.537637 \| \| IDO1 \| Nutlin-3a (-) \| 0.040809 \| 0.412062 \| \| IDO1 \| OSI-027 \| 0.028117 \| 0.469391 \| \| IDO1 \| OSI-930 \| 0.048535 \| 0.222653 \| \| IDO1 \| OSU-03012 \| -0.06292 \| 0.183146 \| \| IDO1 \| Obatoclax Mesylate \| -0.03809 \| 0.439523 \| \| IDO1 \| Olaparib \| -0.10113 \| 0.013586 \| \| IDO1 \| PAC-1 \| 0.033622 \| 0.458351 \| \| IDO1 \| PD-0325901 \| 0.010381 \| 0.821265 \| \| IDO1 \| PD-0332991 \| -0.02781 \| 0.655641 \| \| IDO1 \| PD-173074 \| 0.029543 \| 0.859194 \| \| IDO1 \| PF-4708671 \| 0.033034 \| 0.762689 \| \| IDO1 \| PF-562271 \| -0.0916 \| 0.063971 \| \| IDO1 \| PFI-1 \| 0.031492 \| 0.544608 \| \| IDO1 \| PHA-665752 \| -0.03243 \| 0.842804 \| \| IDO1 \| PHA-793887 \| 0.06571 \| 0.066547 \| \| IDO1 \| PI-103 \| 0.015587 \| 0.69943 \| \| IDO1 \| PIK-93 \| 0.043552 \| 0.237478 \| \| IDO1 \| PLX4720 \| -0.01271 \| 0.790902 \| \| IDO1 \| Paclitaxel \| -0.0897 \| 0.353057 \| \| IDO1 \| Parthenolide \| 0.027782 \| 0.821336 \| \| IDO1 \| Pazopanib \| -0.02723 \| 0.654018 \| \| IDO1 \| Phenformin \| 0.085428 \| 0.020479 \| \| IDO1 \| Pyrimethamine \| 0.063912 \| 0.580611 \| \| IDO1 \| QL-VIII-58 \| 0.002974 \| 0.979412 \| \| IDO1 \| QL-X-138 \| 0.052179 \| 0.167225 \| \| IDO1 \| QL-XI-92 \| 0.060449 \| 0.102158 \| \| IDO1 \| QL-XII-47 \| 0.03536 \| 0.401961 \| \| IDO1 \| QL-XII-61 \| 0.056538 \| 0.407785 \| \| IDO1 \| QS11 \| -0.00439 \| 0.960656 \| \| IDO1 \| RDEA119 \| 0.008038 \| 0.849607 \| \| IDO1 \| RO-3306 \| -0.06871 \| 0.099742 \| \| IDO1 \| Rapamycin \| -0.05208 \| 0.675397 \| \| IDO1 \| Roscovitine \| -0.00899 \| 0.970297 \| \| IDO1 \| Ruxolitinib \| 0.022985 \| 0.696887 \| \| IDO1 \| S-Trityl-L-cysteine \| -0.03153 \| 0.741044 \| \| IDO1 \| SB 216763 \| -0.01117 \| 0.847421 \| \| IDO1 \| SB 505124 \| 0.039468 \| 0.603417 \| \| IDO1 \| SB52334 \| 0.041392 \| 0.396455 \| \| IDO1 \| SB590885 \| 0.028533 \| 0.596191 \| \| IDO1 \| SGC0946 \| -0.03036 \| 0.601782 \| \| IDO1 \| SL 0101-1 \| -0.09583 \| 0.189682 \| \| IDO1 \| SN-38 \| -0.05866 \| 0.173435 \| \| IDO1 \| SNX-2112 \| 0.062694 \| 0.091205 \| \| IDO1 \| STF-62247 \| 0.047878 \| 0.244908 \| \| IDO1 \| Salubrinal \| 0.010863 \| 0.921024 \| \| IDO1 \| Saracatinib \| -0.10481 \| 0.182113 \| \| IDO1 \| Shikonin \| -0.04721 \| 0.374278 \| \| IDO1 \| Sorafenib \| 0.023042 \| 0.862842 \| \| IDO1 \| Sunitinib \| -0.00659 \| 0.951132 \| \| IDO1 \| T0901317 \| 0.038796 \| 0.363665 \| \| IDO1 \| TAE684 \| -0.04256 \| 0.731768 \| \| IDO1 \| TAK-715 \| -0.00513 \| 0.904081 \| \| IDO1 \| TG101348 \| 0.061683 \| 0.091399 \| \| IDO1 \| TGX221 \| -0.2199 \| 0.000146 \| \| IDO1 \| THZ-2-102-1 \| 0.087783 \| 0.01522 \| \| IDO1 \| THZ-2-49 \| 0.027582 \| 0.485738 \| \| IDO1 \| TL-1-85 \| 0.085771 \| 0.017731 \| \| IDO1 \| TL-2-105 \| 0.049249 \| 0.199049 \| \| IDO1 \| TPCA-1 \| 0.05095 \| 0.164528 \| \| IDO1 \| TW 37 \| 0.042259 \| 0.373751 \| \| IDO1 \| Talazoparib \| -0.04453 \| 0.332476 \| \| IDO1 \| Tamoxifen \| -0.04609 \| 0.676185 \| \| IDO1 \| Temozolomide \| -0.03846 \| 0.6001 \| \| IDO1 \| Temsirolimus \| -0.01742 \| 0.766556 \| \| IDO1 \| Thapsigargin \| -0.05079 \| 0.407172 \| \| IDO1 \| Tipifarnib \| -0.03457 \| 0.622771 \| \| IDO1 \| Tivozanib \| 0.012435 \| 0.869036 \| \| IDO1 \| Trametinib \| -0.01724 \| 0.680525 \| \| IDO1 \| Tubastatin A \| 0.100388 \| 0.004707 \| \| IDO1 \| UNC0638 \| 0.092049 \| 0.008777 \| \| IDO1 \| UNC1215 \| -0.00808 \| 0.899874 \| \| IDO1 \| VNLG/124 \| 0.014028 \| 0.772934 \| \| IDO1 \| VX-11e \| 0.057489 \| 0.212308 \| \| IDO1 \| VX-680 \| -0.00734 \| 0.957692 \| \| IDO1 \| VX-702 \| -0.0105 \| 0.974173 \| \| IDO1 \| Veliparib \| -0.03365 \| 0.638235 \| \| IDO1 \| Vinblastine \| -0.00097 \| 0.986789 \| \| IDO1 \| Vinorelbine \| -0.05114 \| 0.341311 \| \| IDO1 \| Vorinostat \| 0.107083 \| 0.003352 \| \| IDO1 \| WH-4-023 \| -0.12916 \| 0.061793 \| \| IDO1 \| WZ-1-84 \| -0.08529 \| 0.258797 \| \| IDO1 \| WZ3105 \| 0.071492 \| 0.047587 \| \| IDO1 \| XAV939 \| -0.04722 \| 0.237214 \| \| IDO1 \| XL-184 \| -0.03433 \| 0.487043 \| \| IDO1 \| XMD11-85h \| 0.048355 \| 0.787089 \| \| IDO1 \| XMD13-2 \| 0.035527 \| 0.35099 \| \| IDO1 \| XMD14-99 \| 0.052622 \| 0.176203 \| \| IDO1 \| XMD15-27 \| 0.032748 \| 0.51657 \| \| IDO1 \| XMD8-85 \| 0.011242 \| 0.916344 \| \| IDO1 \| XMD8-92 \| 0.043309 \| 0.701831 \| \| IDO1 \| Y-39983 \| 0.064213 \| 0.089183 \| \| IDO1 \| YK 4-279 \| -0.02818 \| 0.63824 \| \| IDO1 \| YM155 \| -0.00972 \| 0.889108 \| \| IDO1 \| YM201636 \| 0.057609 \| 0.124515 \| \| IDO1 \| Z-LLNle-CHO \| -0.02421 \| 0.781244 \| \| IDO1 \| ZG-10 \| 0.084643 \| 0.180548 \| \| IDO1 \| ZM-447439 \| -0.01833 \| 0.736812 \| \| IDO1 \| ZSTK474 \| -0.00027 \| 0.995513 \| \| IDO1 \| Zibotentan \| -0.07723 \| 0.529613 \| \| IDO1 \| piperlongumine \| 0.004652 \| 0.936081 \| \| IDO1 \| rTRAIL \| -0.07373 \| 0.245463 \| \| IDO1 \| selumetinib \| -0.00883 \| 0.841788 \| \| IFNG \| (5Z)-7-Oxozeaenol \| -0.00587 \| 0.911831 \| \| IFNG \| 17-AAG \| 0.082501 \| 0.027313 \| \| IFNG \| 5-Fluorouracil \| -0.18911 \| 6.06E-08 \| \| IFNG \| 681640 \| 0.084335 \| 0.132752 \| \| IFNG \| A-443654 \| 0.081859 \| 0.590824 \| \| IFNG \| A-770041 \| 0.0236 \| 0.808971 \| \| IFNG \| AC220 \| -0.03074 \| 0.555166 \| \| IFNG \| AG-014699 \| 0.091728 \| 0.024553 \| \| IFNG \| AICAR \| -0.07824 \| 0.04763 \| \| IFNG \| AKT inhibitor VIII \| 0.040846 \| 0.385467 \| \| IFNG \| AMG-706 \| 0.05119 \| 0.38006 \| \| IFNG \| AP-24534 \| -0.03946 \| 0.361841 \| \| IFNG \| AR-42 \| -0.13399 \| 0.000141 \| \| IFNG \| AS601245 \| 0.022739 \| 0.71386 \| \| IFNG \| AS605240 \| -0.1323 \| 0.000591 \| \| IFNG \| AT-7519 \| -0.10301 \| 0.003683 \| \| IFNG \| ATRA \| -0.00748 \| 0.90515 \| \| IFNG \| AUY922 \| -0.03412 \| 0.543179 \| \| IFNG \| AZ628 \| 0.052443 \| 0.513478 \| \| IFNG \| AZD6482 \| -0.06962 \| 0.1041 \| \| IFNG \| AZD7762 \| -0.04776 \| 0.252297 \| \| IFNG \| AZD8055 \| -0.17029 \| 5.32E-06 \| \| IFNG \| Afatinib \| 0.044096 \| 0.250941 \| \| IFNG \| Axitinib \| 0.017718 \| 0.783871 \| \| IFNG \| BAY 61-3606 \| -0.10409 \| 0.007852 \| \| IFNG \| BEZ235 \| -0.0303 \| 0.56764 \| \| IFNG \| BHG712 \| -0.10716 \| 0.002673 \| \| IFNG \| BI-2536 \| 0.039574 \| 0.736698 \| \| IFNG \| BIRB 0796 \| 0.047684 \| 0.356331 \| \| IFNG \| BIX02189 \| -0.06504 \| 0.079542 \| \| IFNG \| BMS-509744 \| 0.096937 \| 0.260668 \| \| IFNG \| BMS-536924 \| 0.045482 \| 0.492752 \| \| IFNG \| BMS-708163 \| 0.020002 \| 0.654007 \| \| IFNG \| BMS-754807 \| 0.085931 \| 0.078693 \| \| IFNG \| BMS345541 \| -0.12286 \| 0.000496 \| \| IFNG \| BX-795 \| -0.02075 \| 0.696447 \| \| IFNG \| BX-912 \| -0.0541 \| 0.138559 \| \| IFNG \| Belinostat \| -0.11913 \| 0.001038 \| \| IFNG \| Bexarotene \| 0.025598 \| 0.763331 \| \| IFNG \| Bicalutamide \| 0.061885 \| 0.155218 \| \| IFNG \| Bleomycin \| -0.00152 \| 0.985428 \| \| IFNG \| Bleomycin (50 uM) \| 0.080238 \| 0.026963 \| \| IFNG \| Bortezomib \| 0.059577 \| 0.46484 \| \| IFNG \| Bosutinib \| 0.036312 \| 0.52129 \| \| IFNG \| Bryostatin 1 \| 0.059636 \| 0.23309 \| \| IFNG \| CAL-101 \| -0.22266 \| 1.46E-10 \| \| IFNG \| CAY10603 \| -0.1365 \| 0.000101 \| \| IFNG \| CCT007093 \| 0.058929 \| 0.170984 \| \| IFNG \| CCT018159 \| -0.0144 \| 0.820275 \| \| IFNG \| CEP-701 \| -0.0687 \| 0.090058 \| \| IFNG \| CGP-082996 \| 0.040961 \| 0.698462 \| \| IFNG \| CGP-60474 \| 0.011311 \| 0.922178 \| \| IFNG \| CH5424802 \| 0.003926 \| 0.961906 \| \| IFNG \| CHIR-99021 \| 0.097739 \| 0.008197 \| \| IFNG \| CI-1040 \| 0.001738 \| 0.974973 \| \| IFNG \| CMK \| -0.00669 \| 0.965827 \| \| IFNG \| CP466722 \| -0.14096 \| 5.57E-05 \| \| IFNG \| CP724714 \| 0.00974 \| 0.892428 \| \| IFNG \| CUDC-101 \| -0.12655 \| 0.000423 \| \| IFNG \| CX-5461 \| -0.1045 \| 0.003779 \| \| IFNG \| Camptothecin \| -0.02737 \| 0.576635 \| \| IFNG \| Cetuximab \| 0.059933 \| 0.143402 \| \| IFNG \| Cisplatin \| 0.056302 \| 0.245823 \| \| IFNG \| Crizotinib \| 0.047981 \| 0.753996 \| \| IFNG \| Cyclopamine \| 0.049734 \| 0.662815 \| \| IFNG \| Cytarabine \| 0.016543 \| 0.783 \| \| IFNG \| DMOG \| -0.09531 \| 0.021043 \| \| IFNG \| Dabrafenib \| 0.016474 \| 0.739248 \| \| IFNG \| Dasatinib \| 0.083531 \| 0.21011 \| \| IFNG \| Docetaxel \| 0.131189 \| 0.000369 \| \| IFNG \| Doxorubicin \| -0.0102 \| 0.90339 \| \| IFNG \| EHT 1864 \| 0.052375 \| 0.400035 \| \| IFNG \| EKB-569 \| -0.07113 \| 0.071746 \| \| IFNG \| EX-527 \| 0.028742 \| 0.809949 \| \| IFNG \| Elesclomol \| 0.016609 \| 0.74707 \| \| IFNG \| Embelin \| -0.01282 \| 0.870137 \| \| IFNG \| Epothilone B \| 0.056389 \| 0.258959 \| \| IFNG \| Erlotinib \| 0.086002 \| 0.236597 \| \| IFNG \| Etoposide \| -0.02002 \| 0.720109 \| \| IFNG \| FH535 \| 0.042539 \| 0.389261 \| \| IFNG \| FK866 \| -0.1034 \| 0.003984 \| \| IFNG \| FMK \| -0.11694 \| 0.023344 \| \| IFNG \| FR-180204 \| -0.00686 \| 0.924256 \| \| IFNG \| FTI-277 \| 0.033888 \| 0.439162 \| \| IFNG \| Foretinib \| -0.00181 \| 0.97254 \| \| IFNG \| GDC0449 \| -0.0453 \| 0.637036 \| \| IFNG \| GDC0941 \| -0.02564 \| 0.689091 \| \| IFNG \| GNF-2 \| 0.031908 \| 0.909559 \| \| IFNG \| GSK-650394 \| 0.023259 \| 0.788716 \| \| IFNG \| GSK1070916 \| -0.03014 \| 0.444657 \| \| IFNG \| GSK1904529A \| 0.08246 \| 0.057888 \| \| IFNG \| GSK2126458 \| -0.10882 \| 0.002852 \| \| IFNG \| GSK269962A \| 0.024746 \| 0.677214 \| \| IFNG \| GSK429286A \| -0.02418 \| 0.59982 \| \| IFNG \| GSK690693 \| -0.06059 \| 0.106614 \| \| IFNG \| GW 441756 \| 0.028331 \| 0.898037 \| \| IFNG \| GW-2580 \| -0.00376 \| 0.99503 \| \| IFNG \| GW843682X \| 0.029001 \| 0.782281 \| \| IFNG \| Gefitinib \| 0.054209 \| 0.195264 \| \| IFNG \| Gemcitabine \| 0.008014 \| 0.898044 \| \| IFNG \| Genentech Cpd 10 \| -0.06679 \| 0.073185 \| \| IFNG \| HG-5-113-01 \| -0.07224 \| 0.293069 \| \| IFNG \| HG-5-88-01 \| 0.034172 \| 0.84288 \| \| IFNG \| HG-6-64-1 \| 0.021937 \| 0.691463 \| \| IFNG \| I-BET-762 \| -0.17986 \| 1.4E-07 \| \| IFNG \| IOX2 \| 0.070339 \| 0.186369 \| \| IFNG \| IPA-3 \| -0.19367 \| 1.2E-07 \| \| IFNG \| Imatinib \| 0.032812 \| 0.853412 \| \| IFNG \| Ispinesib Mesylate \| -0.06347 \| 0.08804 \| \| IFNG \| JNJ-26854165 \| 0.023702 \| 0.683497 \| \| IFNG \| JNK Inhibitor VIII \| 0.074734 \| 0.073313 \| \| IFNG \| JNK-9L \| -0.0329 \| 0.5917 \| \| IFNG \| JQ1 \| -0.02869 \| 0.531915 \| \| IFNG \| JQ12 \| -0.02959 \| 0.617498 \| \| IFNG \| JW-7-24-1 \| -0.11821 \| 0.000798 \| \| IFNG \| JW-7-52-1 \| 0.085875 \| 0.344276 \| \| IFNG \| KIN001-055 \| 0.025847 \| 0.723771 \| \| IFNG \| KIN001-102 \| -0.16366 \| 2.22E-06 \| \| IFNG \| KIN001-135 \| 0.072111 \| 0.466954 \| \| IFNG \| KIN001-236 \| -0.11428 \| 0.00155 \| \| IFNG \| KIN001-244 \| -0.09701 \| 0.007483 \| \| IFNG \| KIN001-260 \| -0.14596 \| 3.41E-05 \| \| IFNG \| KIN001-266 \| -0.02913 \| 0.572522 \| \| IFNG \| KIN001-270 \| -0.08556 \| 0.025573 \| \| IFNG \| KU-55933 \| -0.04961 \| 0.399548 \| \| IFNG \| LAQ824 \| -0.09356 \| 0.015167 \| \| IFNG \| LFM-A13 \| 0.064594 \| 0.180273 \| \| IFNG \| LY317615 \| -0.04927 \| 0.263402 \| \| IFNG \| Lapatinib \| 0.075125 \| 0.240847 \| \| IFNG \| Lenalidomide \| -0.04923 \| 0.488133 \| \| IFNG \| Linifanib \| 0.011398 \| 0.862765 \| \| IFNG \| Lisitinib \| 0.066807 \| 0.240644 \| \| IFNG \| MG-132 \| 0.056455 \| 0.538875 \| \| IFNG \| MK-2206 \| -0.11814 \| 0.00911 \| \| IFNG \| MLN4924 \| 0.021389 \| 0.740124 \| \| IFNG \| MP470 \| -0.02492 \| 0.595577 \| \| IFNG \| MPS-1-IN-1 \| -0.03647 \| 0.359483 \| \| IFNG \| MS-275 \| 0.013079 \| 0.90279 \| \| IFNG \| Masitinib \| -0.06119 \| 0.107936 \| \| IFNG \| Methotrexate \| -0.09627 \| 0.009134 \| \| IFNG \| Midostaurin \| 0.070005 \| 0.104508 \| \| IFNG \| Mitomycin C \| -0.02975 \| 0.596872 \| \| IFNG \| NG-25 \| -0.07861 \| 0.029809 \| \| IFNG \| NPK76-II-72-1 \| -0.14066 \| 4.68E-05 \| \| IFNG \| NSC-207895 \| -0.08343 \| 0.044834 \| \| IFNG \| NSC-87877 \| 0.066916 \| 0.20575 \| \| IFNG \| NU-7441 \| -0.1263 \| 0.019768 \| \| IFNG \| Navitoclax \| -0.10824 \| 0.003319 \| \| IFNG \| Nilotinib \| -0.00918 \| 0.888465 \| \| IFNG \| Nutlin-3a (-) \| -0.0967 \| 0.024605 \| \| IFNG \| OSI-027 \| -0.12841 \| 0.000289 \| \| IFNG \| OSI-930 \| -0.06549 \| 0.091092 \| \| IFNG \| OSU-03012 \| -0.03997 \| 0.43536 \| \| IFNG \| Obatoclax Mesylate \| -0.02908 \| 0.571534 \| \| IFNG \| Olaparib \| 0.091689 \| 0.026604 \| \| IFNG \| PAC-1 \| -0.12202 \| 0.001829 \| \| IFNG \| PD-0325901 \| 0.043124 \| 0.302889 \| \| IFNG \| PD-0332991 \| 0.019449 \| 0.770664 \| \| IFNG \| PD-173074 \| 0.007002 \| 0.981366 \| \| IFNG \| PF-4708671 \| 0.045414 \| 0.634246 \| \| IFNG \| PF-562271 \| 0.091949 \| 0.062918 \| \| IFNG \| PFI-1 \| -0.03257 \| 0.528874 \| \| IFNG \| PHA-665752 \| 0.063437 \| 0.659461 \| \| IFNG \| PHA-793887 \| -0.14308 \| 3.54E-05 \| \| IFNG \| PI-103 \| -0.13781 \| 9.44E-05 \| \| IFNG \| PIK-93 \| -0.12899 \| 0.000214 \| \| IFNG \| PLX4720 \| 0.030562 \| 0.488718 \| \| IFNG \| Paclitaxel \| 0.048882 \| 0.676172 \| \| IFNG \| Parthenolide \| 0.011443 \| 0.932578 \| \| IFNG \| Pazopanib \| 0.068123 \| 0.182797 \| \| IFNG \| Phenformin \| -0.11478 \| 0.001562 \| \| IFNG \| Pyrimethamine \| 0.038167 \| 0.764866 \| \| IFNG \| QL-VIII-58 \| -0.01101 \| 0.918317 \| \| IFNG \| QL-X-138 \| -0.11595 \| 0.001245 \| \| IFNG \| QL-XI-92 \| -0.16632 \| 1.88E-06 \| \| IFNG \| QL-XII-47 \| -0.11205 \| 0.003062 \| \| IFNG \| QL-XII-61 \| -0.10299 \| 0.090057 \| \| IFNG \| QS11 \| 0.035746 \| 0.58384 \| \| IFNG \| RDEA119 \| 0.047879 \| 0.211196 \| \| IFNG \| RO-3306 \| 0.078429 \| 0.058074 \| \| IFNG \| Rapamycin \| 0.036842 \| 0.791857 \| \| IFNG \| Roscovitine \| -0.00594 \| 0.981758 \| \| IFNG \| Ruxolitinib \| -0.12572 \| 0.00239 \| \| IFNG \| S-Trityl-L-cysteine \| 0.007364 \| 0.948827 \| \| IFNG \| SB 216763 \| 0.09835 \| 0.034911 \| \| IFNG \| SB 505124 \| 0.037623 \| 0.625325 \| \| IFNG \| SB52334 \| 0.021247 \| 0.701226 \| \| IFNG \| SB590885 \| 0.029677 \| 0.579018 \| \| IFNG \| SGC0946 \| 0.026091 \| 0.664962 \| \| IFNG \| SL 0101-1 \| 0.006113 \| 0.952767 \| \| IFNG \| SN-38 \| -0.00431 \| 0.94046 \| \| IFNG \| SNX-2112 \| -0.14413 \| 4.41E-05 \| \| IFNG \| STF-62247 \| -0.09694 \| 0.012564 \| \| IFNG \| Salubrinal \| 0.043089 \| 0.638103 \| \| IFNG \| Saracatinib \| 0.056275 \| 0.513029 \| \| IFNG \| Shikonin \| 0.021491 \| 0.726069 \| \| IFNG \| Sorafenib \| 0.055403 \| 0.622376 \| \| IFNG \| Sunitinib \| 0.036489 \| 0.664082 \| \| IFNG \| T0901317 \| -0.0447 \| 0.288374 \| \| IFNG \| TAE684 \| 0.112909 \| 0.205855 \| \| IFNG \| TAK-715 \| -0.12366 \| 0.00049 \| \| IFNG \| TG101348 \| -0.14592 \| 2.87E-05 \| \| IFNG \| TGX221 \| 0.065396 \| 0.323811 \| \| IFNG \| THZ-2-102-1 \| -0.12441 \| 0.000459 \| \| IFNG \| THZ-2-49 \| -0.07516 \| 0.042012 \| \| IFNG \| TL-1-85 \| -0.07588 \| 0.037291 \| \| IFNG \| TL-2-105 \| -0.05212 \| 0.172522 \| \| IFNG \| TPCA-1 \| -0.21685 \| 1.8E-10 \| \| IFNG \| TW 37 \| 0.018346 \| 0.73549 \| \| IFNG \| Talazoparib \| 0.046049 \| 0.314622 \| \| IFNG \| Tamoxifen \| -0.04905 \| 0.651968 \| \| IFNG \| Temozolomide \| -0.13569 \| 0.002462 \| \| IFNG \| Temsirolimus \| -0.07354 \| 0.106551 \| \| IFNG \| Thapsigargin \| 0.053311 \| 0.376305 \| \| IFNG \| Tipifarnib \| -0.02807 \| 0.702658 \| \| IFNG \| Tivozanib \| 0.000285 \| 0.997365 \| \| IFNG \| Trametinib \| 0.090686 \| 0.013897 \| \| IFNG \| Tubastatin A \| -0.10149 \| 0.004253 \| \| IFNG \| UNC0638 \| -0.07131 \| 0.045099 \| \| IFNG \| UNC1215 \| 0.021041 \| 0.716731 \| \| IFNG \| VNLG/124 \| -0.01645 \| 0.732753 \| \| IFNG \| VX-11e \| -0.04353 \| 0.370231 \| \| IFNG \| VX-680 \| 0.035893 \| 0.734946 \| \| IFNG \| VX-702 \| -0.03034 \| 0.90325 \| \| IFNG \| Veliparib \| 0.025913 \| 0.728209 \| \| IFNG \| Vinblastine \| -0.00737 \| 0.894394 \| \| IFNG \| Vinorelbine \| 0.012441 \| 0.858242 \| \| IFNG \| Vorinostat \| -0.14595 \| 5.25E-05 \| \| IFNG \| WH-4-023 \| 0.066538 \| 0.395807 \| \| IFNG \| WZ-1-84 \| 0.094666 \| 0.200874 \| \| IFNG \| WZ3105 \| -0.07296 \| 0.042965 \| \| IFNG \| XAV939 \| 0.070112 \| 0.071027 \| \| IFNG \| XL-184 \| 0.019304 \| 0.719441 \| \| IFNG \| XMD11-85h \| -0.0647 \| 0.685295 \| \| IFNG \| XMD13-2 \| -0.14234 \| 4.84E-05 \| \| IFNG \| XMD14-99 \| -0.09319 \| 0.011951 \| \| IFNG \| XMD15-27 \| -0.05079 \| 0.277746 \| \| IFNG \| XMD8-85 \| -0.04699 \| 0.593164 \| \| IFNG \| XMD8-92 \| -0.07191 \| 0.472288 \| \| IFNG \| Y-39983 \| -0.06286 \| 0.096793 \| \| IFNG \| YK 4-279 \| -0.00306 \| 0.969674 \| \| IFNG \| YM155 \| -0.05657 \| 0.253564 \| \| IFNG \| YM201636 \| -0.07322 \| 0.047124 \| \| IFNG \| Z-LLNle-CHO \| -0.03142 \| 0.710855 \| \| IFNG \| ZG-10 \| -0.01111 \| 0.89921 \| \| IFNG \| ZM-447439 \| -0.02304 \| 0.66294 \| \| IFNG \| ZSTK474 \| -0.15488 \| 1.04E-05 \| \| IFNG \| Zibotentan \| 0.026867 \| 0.93003 \| \| IFNG \| piperlongumine \| 0.003509 \| 0.953399 \| \| IFNG \| rTRAIL \| 0.060037 \| 0.37576 \| \| IFNG \| selumetinib \| 0.066577 \| 0.07476 \| \| PRF1 \| (5Z)-7-Oxozeaenol \| -0.02173 \| 0.644882 \| \| PRF1 \| 17-AAG \| 0.094465 \| 0.010931 \| \| PRF1 \| 5-Fluorouracil \| -0.26061 \| 2.92E-14 \| \| PRF1 \| 681640 \| -0.0069 \| 0.925951 \| \| PRF1 \| A-443654 \| 0.040814 \| 0.833982 \| \| PRF1 \| A-770041 \| 0.034248 \| 0.713164 \| \| PRF1 \| AC220 \| -0.02824 \| 0.591956 \| \| PRF1 \| AG-014699 \| 0.070105 \| 0.092287 \| \| PRF1 \| AICAR \| -0.13761 \| 0.000291 \| \| PRF1 \| AKT inhibitor VIII \| 0.091359 \| 0.031109 \| \| PRF1 \| AMG-706 \| 0.076233 \| 0.146832 \| \| PRF1 \| AP-24534 \| -0.07338 \| 0.069571 \| \| PRF1 \| AR-42 \| -0.14896 \| 2.12E-05 \| \| PRF1 \| AS601245 \| 0.046466 \| 0.390793 \| \| PRF1 \| AS605240 \| -0.07963 \| 0.050291 \| \| PRF1 \| AT-7519 \| -0.1988 \| 7.29E-09 \| \| PRF1 \| ATRA \| -0.09096 \| 0.038786 \| \| PRF1 \| AUY922 \| -0.00423 \| 0.955445 \| \| PRF1 \| AZ628 \| 0.077166 \| 0.296329 \| \| PRF1 \| AZD6482 \| 0.020126 \| 0.718602 \| \| PRF1 \| AZD7762 \| -0.192 \| 2.45E-07 \| \| PRF1 \| AZD8055 \| -0.16484 \| 1.1E-05 \| \| PRF1 \| Afatinib \| 0.07945 \| 0.030726 \| \| PRF1 \| Axitinib \| -0.08325 \| 0.075494 \| \| PRF1 \| BAY 61-3606 \| -0.0668 \| 0.100843 \| \| PRF1 \| BEZ235 \| -0.01637 \| 0.776792 \| \| PRF1 \| BHG712 \| -0.10802 \| 0.002459 \| \| PRF1 \| BI-2536 \| -0.02299 \| 0.861609 \| \| PRF1 \| BIRB 0796 \| 0.085143 \| 0.072807 \| \| PRF1 \| BIX02189 \| -0.10652 \| 0.003021 \| \| PRF1 \| BMS-509744 \| 0.083945 \| 0.340181 \| \| PRF1 \| BMS-536924 \| -0.03153 \| 0.676455 \| \| PRF1 \| BMS-708163 \| 0.08186 \| 0.036931 \| \| PRF1 \| BMS-754807 \| 0.098784 \| 0.037291 \| \| PRF1 \| BMS345541 \| -0.16524 \| 2.01E-06 \| \| PRF1 \| BX-795 \| -0.10342 \| 0.012445 \| \| PRF1 \| BX-912 \| -0.16388 \| 2.07E-06 \| \| PRF1 \| Belinostat \| -0.13887 \| 0.000115 \| \| PRF1 \| Bexarotene \| 0.027067 \| 0.749442 \| \| PRF1 \| Bicalutamide \| 0.092941 \| 0.025247 \| \| PRF1 \| Bleomycin \| -0.03288 \| 0.582582 \| \| PRF1 \| Bleomycin (50 uM) \| 0.036063 \| 0.343154 \| \| PRF1 \| Bortezomib \| -0.02651 \| 0.781413 \| \| PRF1 \| Bosutinib \| -0.07388 \| 0.120951 \| \| PRF1 \| Bryostatin 1 \| 0.094518 \| 0.040956 \| \| PRF1 \| CAL-101 \| -0.14477 \| 5.41E-05 \| \| PRF1 \| CAY10603 \| -0.15238 \| 1.27E-05 \| \| PRF1 \| CCT007093 \| 0.053344 \| 0.221509 \| \| PRF1 \| CCT018159 \| -0.04733 \| 0.356305 \| \| PRF1 \| CEP-701 \| -0.19128 \| 2.68E-07 \| \| PRF1 \| CGP-082996 \| -0.00663 \| 0.961555 \| \| PRF1 \| CGP-60474 \| -0.09168 \| 0.241326 \| \| PRF1 \| CH5424802 \| -0.54175 \| 9.55E-68 \| \| PRF1 \| CHIR-99021 \| 0.088301 \| 0.017826 \| \| PRF1 \| CI-1040 \| 0.013764 \| 0.790078 \| \| PRF1 \| CMK \| -0.00216 \| 0.988871 \| \| PRF1 \| CP466722 \| -0.20822 \| 1.21E-09 \| \| PRF1 \| CP724714 \| 0.030586 \| 0.601976 \| \| PRF1 \| CUDC-101 \| -0.13067 \| 0.000267 \| \| PRF1 \| CX-5461 \| -0.12299 \| 0.00058 \| \| PRF1 \| Camptothecin \| -0.11765 \| 0.003155 \| \| PRF1 \| Cetuximab \| 0.073264 \| 0.068272 \| \| PRF1 \| Cisplatin \| -0.00483 \| 0.942046 \| \| PRF1 \| Crizotinib \| -0.37506 \| 1.32E-11 \| \| PRF1 \| Cyclopamine \| -0.0852 \| 0.381695 \| \| PRF1 \| Cytarabine \| -0.01349 \| 0.825678 \| \| PRF1 \| DMOG \| -0.10948 \| 0.006965 \| \| PRF1 \| Dabrafenib \| 0.020274 \| 0.676877 \| \| PRF1 \| Dasatinib \| 0.084437 \| 0.20435 \| \| PRF1 \| Docetaxel \| 0.118677 \| 0.001327 \| \| PRF1 \| Doxorubicin \| -0.01769 \| 0.818615 \| \| PRF1 \| EHT 1864 \| 0.025715 \| 0.736391 \| \| PRF1 \| EKB-569 \| -0.13877 \| 0.000193 \| \| PRF1 \| EX-527 \| 0.013938 \| 0.916605 \| \| PRF1 \| Elesclomol \| 0.027375 \| 0.573068 \| \| PRF1 \| Embelin \| 0.015101 \| 0.842531 \| \| PRF1 \| Epothilone B \| 0.054836 \| 0.273442 \| \| PRF1 \| Erlotinib \| 0.106198 \| 0.133766 \| \| PRF1 \| Etoposide \| -0.08764 \| 0.042233 \| \| PRF1 \| FH535 \| 0.082874 \| 0.059275 \| \| PRF1 \| FK866 \| -0.15918 \| 5.88E-06 \| \| PRF1 \| FMK \| -0.0778 \| 0.182535 \| \| PRF1 \| FR-180204 \| 0.006686 \| 0.926491 \| \| PRF1 \| FTI-277 \| 0.050091 \| 0.236716 \| \| PRF1 \| Foretinib \| -0.06696 \| 0.097303 \| \| PRF1 \| GDC0449 \| -0.04057 \| 0.679903 \| \| PRF1 \| GDC0941 \| 0.014873 \| 0.831802 \| \| PRF1 \| GNF-2 \| -0.00996 \| 0.980215 \| \| PRF1 \| GSK-650394 \| 0.029701 \| 0.712034 \| \| PRF1 \| GSK1070916 \| -0.12178 \| 0.0007 \| \| PRF1 \| GSK1904529A \| 0.121508 \| 0.003631 \| \| PRF1 \| GSK2126458 \| -0.10794 \| 0.003105 \| \| PRF1 \| GSK269962A \| -0.02736 \| 0.637754 \| \| PRF1 \| GSK429286A \| -0.14645 \| 8.19E-05 \| \| PRF1 \| GSK690693 \| -0.03867 \| 0.320633 \| \| PRF1 \| GW 441756 \| 0.001194 \| 0.995843 \| \| PRF1 \| GW-2580 \| 0.034041 \| 0.937849 \| \| PRF1 \| GW843682X \| -0.05467 \| 0.554509 \| \| PRF1 \| Gefitinib \| 0.078462 \| 0.053859 \| \| PRF1 \| Gemcitabine \| -0.0718 \| 0.12123 \| \| PRF1 \| Genentech Cpd 10 \| -0.16353 \| 3.12E-06 \| \| PRF1 \| HG-5-113-01 \| -0.21054 \| 0.000357 \| \| PRF1 \| HG-5-88-01 \| -0.24012 \| 6.85E-05 \| \| PRF1 \| HG-6-64-1 \| 0.011596 \| 0.846643 \| \| PRF1 \| I-BET-762 \| -0.14771 \| 1.77E-05 \| \| PRF1 \| IOX2 \| 0.084455 \| 0.101997 \| \| PRF1 \| IPA-3 \| -0.12661 \| 0.000771 \| \| PRF1 \| Imatinib \| -0.01134 \| 0.958699 \| \| PRF1 \| Ispinesib Mesylate \| -0.15845 \| 6.44E-06 \| \| PRF1 \| JNJ-26854165 \| -0.0208 \| 0.72463 \| \| PRF1 \| JNK Inhibitor VIII \| 0.10628 \| 0.008609 \| \| PRF1 \| JNK-9L \| -0.0528 \| 0.329894 \| \| PRF1 \| JQ1 \| -0.02305 \| 0.626004 \| \| PRF1 \| JQ12 \| -0.03067 \| 0.602271 \| \| PRF1 \| JW-7-24-1 \| -0.17295 \| 5.8E-07 \| \| PRF1 \| JW-7-52-1 \| -0.00844 \| 0.951016 \| \| PRF1 \| KIN001-055 \| 0.04394 \| 0.480733 \| \| PRF1 \| KIN001-102 \| -0.14345 \| 3.72E-05 \| \| PRF1 \| KIN001-135 \| 0.142794 \| 0.09408 \| \| PRF1 \| KIN001-236 \| -0.07191 \| 0.053178 \| \| PRF1 \| KIN001-244 \| -0.09899 \| 0.006278 \| \| PRF1 \| KIN001-260 \| -0.19097 \| 3.79E-08 \| \| PRF1 \| KIN001-266 \| -0.02509 \| 0.63345 \| \| PRF1 \| KIN001-270 \| -0.08457 \| 0.027497 \| \| PRF1 \| KU-55933 \| -0.12504 \| 0.008201 \| \| PRF1 \| LAQ824 \| -0.12455 \| 0.000961 \| \| PRF1 \| LFM-A13 \| 0.09824 \| 0.031402 \| \| PRF1 \| LY317615 \| -0.06386 \| 0.134533 \| \| PRF1 \| Lapatinib \| 0.104391 \| 0.094658 \| \| PRF1 \| Lenalidomide \| 0.01762 \| 0.856801 \| \| PRF1 \| Linifanib \| -0.04289 \| 0.412914 \| \| PRF1 \| Lisitinib \| 0.084844 \| 0.106773 \| \| PRF1 \| MG-132 \| -0.00897 \| 0.942702 \| \| PRF1 \| MK-2206 \| -0.04132 \| 0.434377 \| \| PRF1 \| MLN4924 \| 0.031507 \| 0.607627 \| \| PRF1 \| MP470 \| -0.02464 \| 0.599797 \| \| PRF1 \| MPS-1-IN-1 \| -0.36056 \| 3.79E-27 \| \| PRF1 \| MS-275 \| -0.16929 \| 0.014577 \| \| PRF1 \| Masitinib \| -0.17721 \| 5.6E-07 \| \| PRF1 \| Methotrexate \| -0.33815 \| 7.11E-23 \| \| PRF1 \| Midostaurin \| 0.046735 \| 0.304546 \| \| PRF1 \| Mitomycin C \| -0.0897 \| 0.048125 \| \| PRF1 \| NG-25 \| -0.1286 \| 0.000257 \| \| PRF1 \| NPK76-II-72-1 \| -0.13051 \| 0.000168 \| \| PRF1 \| NSC-207895 \| -0.01222 \| 0.824736 \| \| PRF1 \| NSC-87877 \| 0.069866 \| 0.183067 \| \| PRF1 \| NU-7441 \| -0.06043 \| 0.358575 \| \| PRF1 \| Navitoclax \| -0.17634 \| 9.33E-07 \| \| PRF1 \| Nilotinib \| -0.12101 \| 0.005128 \| \| PRF1 \| Nutlin-3a (-) \| -0.10691 \| 0.011692 \| \| PRF1 \| OSI-027 \| -0.17642 \| 4.25E-07 \| \| PRF1 \| OSI-930 \| -0.10035 \| 0.007401 \| \| PRF1 \| OSU-03012 \| -0.05507 \| 0.254108 \| \| PRF1 \| Obatoclax Mesylate \| -0.06632 \| 0.143475 \| \| PRF1 \| Olaparib \| 0.020868 \| 0.669199 \| \| PRF1 \| PAC-1 \| -0.08016 \| 0.049055 \| \| PRF1 \| PD-0325901 \| 0.072896 \| 0.067747 \| \| PRF1 \| PD-0332991 \| -0.03353 \| 0.575962 \| \| PRF1 \| PD-173074 \| -0.03325 \| 0.833705 \| \| PRF1 \| PF-4708671 \| 0.027057 \| 0.817983 \| \| PRF1 \| PF-562271 \| 0.054446 \| 0.315193 \| \| PRF1 \| PFI-1 \| -0.04603 \| 0.342052 \| \| PRF1 \| PHA-665752 \| 0.085323 \| 0.531321 \| \| PRF1 \| PHA-793887 \| -0.21831 \| 1.22E-10 \| \| PRF1 \| PI-103 \| -0.12882 \| 0.000275 \| \| PRF1 \| PIK-93 \| -0.13582 \| 9.38E-05 \| \| PRF1 \| PLX4720 \| 0.015527 \| 0.740533 \| \| PRF1 \| Paclitaxel \| -0.02953 \| 0.825025 \| \| PRF1 \| Parthenolide \| -0.04849 \| 0.65331 \| \| PRF1 \| Pazopanib \| 0.061109 \| 0.241126 \| \| PRF1 \| Phenformin \| -0.12539 \| 0.000518 \| \| PRF1 \| Pyrimethamine \| -0.11757 \| 0.247909 \| \| PRF1 \| QL-VIII-58 \| -0.01704 \| 0.864162 \| \| PRF1 \| QL-X-138 \| -0.16887 \| 1.55E-06 \| \| PRF1 \| QL-XI-92 \| -0.22122 \| 1.17E-10 \| \| PRF1 \| QL-XII-47 \| -0.13508 \| 0.000293 \| \| PRF1 \| QL-XII-61 \| -0.13715 \| 0.018036 \| \| PRF1 \| QS11 \| 0.026913 \| 0.699048 \| \| PRF1 \| RDEA119 \| 0.066697 \| 0.072599 \| \| PRF1 \| RO-3306 \| 0.072601 \| 0.081231 \| \| PRF1 \| Rapamycin \| -0.00703 \| 0.970447 \| \| PRF1 \| Roscovitine \| -0.05311 \| 0.757591 \| \| PRF1 \| Ruxolitinib \| -0.23575 \| 8.47E-11 \| \| PRF1 \| S-Trityl-L-cysteine \| -0.09162 \| 0.232689 \| \| PRF1 \| SB 216763 \| 0.09279 \| 0.048011 \| \| PRF1 \| SB 505124 \| 0.02598 \| 0.762922 \| \| PRF1 \| SB52334 \| 0.071227 \| 0.101851 \| \| PRF1 \| SB590885 \| 0.022325 \| 0.684751 \| \| PRF1 \| SGC0946 \| 0.083155 \| 0.071662 \| \| PRF1 \| SL 0101-1 \| 0.074938 \| 0.305452 \| \| PRF1 \| SN-38 \| -0.10271 \| 0.00955 \| \| PRF1 \| SNX-2112 \| -0.19296 \| 2.68E-08 \| \| PRF1 \| STF-62247 \| -0.17192 \| 3.74E-06 \| \| PRF1 \| Salubrinal \| -0.00824 \| 0.9414 \| \| PRF1 \| Saracatinib \| 0.032605 \| 0.725582 \| \| PRF1 \| Shikonin \| -0.06103 \| 0.225909 \| \| PRF1 \| Sorafenib \| 0.027886 \| 0.829764 \| \| PRF1 \| Sunitinib \| -0.10587 \| 0.12407 \| \| PRF1 \| T0901317 \| -0.08153 \| 0.039012 \| \| PRF1 \| TAE684 \| -0.32294 \| 2.73E-08 \| \| PRF1 \| TAK-715 \| -0.1599 \| 5.08E-06 \| \| PRF1 \| TG101348 \| -0.19655 \| 1.04E-08 \| \| PRF1 \| TGX221 \| 0.095212 \| 0.135163 \| \| PRF1 \| THZ-2-102-1 \| -0.17297 \| 7.59E-07 \| \| PRF1 \| THZ-2-49 \| -0.14618 \| 4.22E-05 \| \| PRF1 \| TL-1-85 \| -0.13862 \| 8.05E-05 \| \| PRF1 \| TL-2-105 \| -0.07828 \| 0.034747 \| \| PRF1 \| TPCA-1 \| -0.23653 \| 2.72E-12 \| \| PRF1 \| TW 37 \| -0.00084 \| 0.990425 \| \| PRF1 \| Talazoparib \| -0.06345 \| 0.147311 \| \| PRF1 \| Tamoxifen \| -0.03726 \| 0.757048 \| \| PRF1 \| Temozolomide \| -0.24279 \| 1.6E-10 \| \| PRF1 \| Temsirolimus \| -0.09772 \| 0.023457 \| \| PRF1 \| Thapsigargin \| 0.074932 \| 0.168975 \| \| PRF1 \| Tipifarnib \| 0.002215 \| 0.981043 \| \| PRF1 \| Tivozanib \| -0.02122 \| 0.754197 \| \| PRF1 \| Trametinib \| 0.110348 \| 0.002409 \| \| PRF1 \| Tubastatin A \| -0.13988 \| 6.4E-05 \| \| PRF1 \| UNC0638 \| -0.11356 \| 0.001069 \| \| PRF1 \| UNC1215 \| 0.086316 \| 0.05906 \| \| PRF1 \| VNLG/124 \| -0.08719 \| 0.026812 \| \| PRF1 \| VX-11e \| -0.01943 \| 0.726853 \| \| PRF1 \| VX-680 \| -0.09113 \| 0.295131 \| \| PRF1 \| VX-702 \| -0.01114 \| 0.972826 \| \| PRF1 \| Veliparib \| 0.044165 \| 0.519897 \| \| PRF1 \| Vinblastine \| -0.13924 \| 0.00044 \| \| PRF1 \| Vinorelbine \| -0.00226 \| 0.976968 \| \| PRF1 \| Vorinostat \| -0.22626 \| 1.64E-10 \| \| PRF1 \| WH-4-023 \| 0.060125 \| 0.450554 \| \| PRF1 \| WZ-1-84 \| 0.092206 \| 0.21621 \| \| PRF1 \| WZ3105 \| -0.16057 \| 3.56E-06 \| \| PRF1 \| XAV939 \| 0.095871 \| 0.011729 \| \| PRF1 \| XL-184 \| -0.02078 \| 0.697132 \| \| PRF1 \| XMD11-85h \| -0.1489 \| 0.119514 \| \| PRF1 \| XMD13-2 \| -0.23287 \| 8.5E-12 \| \| PRF1 \| XMD14-99 \| -0.43359 \| 3.33E-41 \| \| PRF1 \| XMD15-27 \| -0.33918 \| 2.46E-22 \| \| PRF1 \| XMD8-85 \| -0.01404 \| 0.894883 \| \| PRF1 \| XMD8-92 \| -0.15367 \| 0.047452 \| \| PRF1 \| Y-39983 \| -0.1217 \| 0.000749 \| \| PRF1 \| YK 4-279 \| -0.09457 \| 0.045077 \| \| PRF1 \| YM155 \| -0.0191 \| 0.757791 \| \| PRF1 \| YM201636 \| -0.04814 \| 0.205972 \| \| PRF1 \| Z-LLNle-CHO \| -0.14277 \| 0.027897 \| \| PRF1 \| ZG-10 \| -0.08784 \| 0.161766 \| \| PRF1 \| ZM-447439 \| -0.10323 \| 0.014123 \| \| PRF1 \| ZSTK474 \| -0.12637 \| 0.000372 \| \| PRF1 \| Zibotentan \| 0.033755 \| 0.902045 \| \| PRF1 \| piperlongumine \| -0.00339 \| 0.955343 \| \| PRF1 \| rTRAIL \| 0.053291 \| 0.447891 \| \| PRF1 \| selumetinib \| 0.073544 \| 0.047202 \| \| STAT1 \| (5Z)-7-Oxozeaenol \| -0.04118 \| 0.341467 \| \| STAT1 \| 17-AAG \| -0.14253 \| 8.97E-05 \| \| STAT1 \| 5-Fluorouracil \| 0.058135 \| 0.118629 \| \| STAT1 \| 681640 \| -0.00226 \| 0.977927 \| \| STAT1 \| A-443654 \| 0.022525 \| 0.924585 \| \| STAT1 \| A-770041 \| -0.07749 \| 0.323423 \| \| STAT1 \| AC220 \| 0.082411 \| 0.054984 \| \| STAT1 \| AG-014699 \| -0.00533 \| 0.919487 \| \| STAT1 \| AICAR \| 0.039908 \| 0.340061 \| \| STAT1 \| AKT inhibitor VIII \| -0.02307 \| 0.649713 \| \| STAT1 \| AMG-706 \| 0.000976 \| 0.991571 \| \| STAT1 \| AP-24534 \| 0.026891 \| 0.55073 \| \| STAT1 \| AR-42 \| 0.126051 \| 0.000354 \| \| STAT1 \| AS601245 \| -0.05993 \| 0.24567 \| \| STAT1 \| AS605240 \| -0.00239 \| 0.965453 \| \| STAT1 \| AT-7519 \| 0.084417 \| 0.018428 \| \| STAT1 \| ATRA \| 0.039483 \| 0.438947 \| \| STAT1 \| AUY922 \| -0.03245 \| 0.567585 \| \| STAT1 \| AZ628 \| 0.017442 \| 0.851333 \| \| STAT1 \| AZD6482 \| -0.11822 \| 0.002711 \| \| STAT1 \| AZD7762 \| -0.00746 \| 0.878327 \| \| STAT1 \| AZD8055 \| 0.054003 \| 0.181411 \| \| STAT1 \| Afatinib \| -0.10073 \| 0.005349 \| \| STAT1 \| Axitinib \| 0.104918 \| 0.019831 \| \| STAT1 \| BAY 61-3606 \| 0.049634 \| 0.235195 \| \| STAT1 \| BEZ235 \| -0.08103 \| 0.070489 \| \| STAT1 \| BHG712 \| 0.079647 \| 0.028348 \| \| STAT1 \| BI-2536 \| 0.027328 \| 0.829842 \| \| STAT1 \| BIRB 0796 \| 0.007846 \| 0.900754 \| \| STAT1 \| BIX02189 \| 0.095069 \| 0.008572 \| \| STAT1 \| BMS-509744 \| 0.061142 \| 0.511974 \| \| STAT1 \| BMS-536924 \| 0.062869 \| 0.284029 \| \| STAT1 \| BMS-708163 \| -0.04545 \| 0.26958 \| \| STAT1 \| BMS-754807 \| 0.140692 \| 0.001568 \| \| STAT1 \| BMS345541 \| 0.115321 \| 0.001113 \| \| STAT1 \| BX-795 \| 0.053593 \| 0.239423 \| \| STAT1 \| BX-912 \| 0.144366 \| 3.27E-05 \| \| STAT1 \| Belinostat \| 0.10794 \| 0.003122 \| \| STAT1 \| Bexarotene \| -0.03657 \| 0.638151 \| \| STAT1 \| Bicalutamide \| -0.06237 \| 0.151635 \| \| STAT1 \| Bleomycin \| -0.07255 \| 0.14951 \| \| STAT1 \| Bleomycin (50 uM) \| -0.10496 \| 0.003367 \| \| STAT1 \| Bortezomib \| -0.17306 \| 0.007381 \| \| STAT1 \| Bosutinib \| -0.02358 \| 0.704026 \| \| STAT1 \| Bryostatin 1 \| -0.03823 \| 0.479035 \| \| STAT1 \| CAL-101 \| -0.04145 \| 0.293657 \| \| STAT1 \| CAY10603 \| 0.123955 \| 0.000441 \| \| STAT1 \| CCT007093 \| -0.05352 \| 0.219758 \| \| STAT1 \| CCT018159 \| -0.06108 \| 0.210659 \| \| STAT1 \| CEP-701 \| 0.022727 \| 0.614896 \| \| STAT1 \| CGP-082996 \| -0.00832 \| 0.9511 \| \| STAT1 \| CGP-60474 \| -0.15475 \| 0.026245 \| \| STAT1 \| CH5424802 \| -0.06047 \| 0.256396 \| \| STAT1 \| CHIR-99021 \| -0.12502 \| 0.000555 \| \| STAT1 \| CI-1040 \| -0.03864 \| 0.40214 \| \| STAT1 \| CMK \| -0.07836 \| 0.467244 \| \| STAT1 \| CP466722 \| 0.080285 \| 0.025734 \| \| STAT1 \| CP724714 \| 0.010633 \| 0.880409 \| \| STAT1 \| CUDC-101 \| 0.100933 \| 0.005486 \| \| STAT1 \| CX-5461 \| 0.143725 \| 4.99E-05 \| \| STAT1 \| Camptothecin \| 0.038664 \| 0.405516 \| \| STAT1 \| Cetuximab \| -0.1083 \| 0.005251 \| \| STAT1 \| Cisplatin \| -0.00285 \| 0.966455 \| \| STAT1 \| Crizotinib \| -0.05709 \| 0.697389 \| \| STAT1 \| Cyclopamine \| -0.03067 \| 0.809812 \| \| STAT1 \| Cytarabine \| 0.10265 \| 0.020158 \| \| STAT1 \| DMOG \| -0.00425 \| 0.942843 \| \| STAT1 \| Dabrafenib \| -0.0226 \| 0.639248 \| \| STAT1 \| Dasatinib \| -0.12087 \| 0.057352 \| \| STAT1 \| Docetaxel \| -0.05605 \| 0.143564 \| \| STAT1 \| Doxorubicin \| 0.007773 \| 0.926193 \| \| STAT1 \| EHT 1864 \| 0.045685 \| 0.478756 \| \| STAT1 \| EKB-569 \| 0.040754 \| 0.331624 \| \| STAT1 \| EX-527 \| 0.013711 \| 0.917825 \| \| STAT1 \| Elesclomol \| 0.010417 \| 0.844272 \| \| STAT1 \| Embelin \| -0.00434 \| 0.960601 \| \| STAT1 \| Epothilone B \| 0.003 \| 0.962722 \| \| STAT1 \| Erlotinib \| 0.014806 \| 0.862594 \| \| STAT1 \| Etoposide \| 0.037063 \| 0.461136 \| \| STAT1 \| FH535 \| -0.00985 \| 0.87096 \| \| STAT1 \| FK866 \| 0.112641 \| 0.001623 \| \| STAT1 \| FMK \| 0.024664 \| 0.74992 \| \| STAT1 \| FR-180204 \| 0.030191 \| 0.597338 \| \| STAT1 \| FTI-277 \| -0.08958 \| 0.025829 \| \| STAT1 \| Foretinib \| 0.099758 \| 0.009345 \| \| STAT1 \| GDC0449 \| -0.0111 \| 0.936295 \| \| STAT1 \| GDC0941 \| 0.016605 \| 0.808566 \| \| STAT1 \| GNF-2 \| 0.013911 \| 0.968513 \| \| STAT1 \| GSK-650394 \| 0.082276 \| 0.170309 \| \| STAT1 \| GSK1070916 \| 0.185379 \| 1.21E-07 \| \| STAT1 \| GSK1904529A \| -0.00823 \| 0.885115 \| \| STAT1 \| GSK2126458 \| 0.067752 \| 0.072428 \| \| STAT1 \| GSK269962A \| 0.01504 \| 0.815852 \| \| STAT1 \| GSK429286A \| 0.040642 \| 0.347434 \| \| STAT1 \| GSK690693 \| 0.149908 \| 2.25E-05 \| \| STAT1 \| GW 441756 \| -0.02833 \| 0.898037 \| \| STAT1 \| GW-2580 \| 0.019007 \| 0.969305 \| \| STAT1 \| GW843682X \| 0.046296 \| 0.63128 \| \| STAT1 \| Gefitinib \| -0.10026 \| 0.011836 \| \| STAT1 \| Gemcitabine \| -0.03378 \| 0.521823 \| \| STAT1 \| Genentech Cpd 10 \| 0.1078 \| 0.002766 \| \| STAT1 \| HG-5-113-01 \| 0.09863 \| 0.128052 \| \| STAT1 \| HG-5-88-01 \| 0.055849 \| 0.693571 \| \| STAT1 \| HG-6-64-1 \| -0.00562 \| 0.929499 \| \| STAT1 \| I-BET-762 \| 0.137137 \| 7.11E-05 \| \| STAT1 \| IOX2 \| -0.01588 \| 0.823804 \| \| STAT1 \| IPA-3 \| 0.011534 \| 0.802011 \| \| STAT1 \| Imatinib \| 0.041758 \| 0.810094 \| \| STAT1 \| Ispinesib Mesylate \| 0.133017 \| 0.000178 \| \| STAT1 \| JNJ-26854165 \| -0.04478 \| 0.383606 \| \| STAT1 \| JNK Inhibitor VIII \| -0.0801 \| 0.053464 \| \| STAT1 \| JNK-9L \| -0.007 \| 0.929716 \| \| STAT1 \| JQ1 \| 0.016554 \| 0.73695 \| \| STAT1 \| JQ12 \| 0.047452 \| 0.383259 \| \| STAT1 \| JW-7-24-1 \| 0.080691 \| 0.024739 \| \| STAT1 \| JW-7-52-1 \| -0.05428 \| 0.602038 \| \| STAT1 \| KIN001-055 \| -0.0009 \| 0.991167 \| \| STAT1 \| KIN001-102 \| 0.09743 \| 0.005891 \| \| STAT1 \| KIN001-135 \| 0.082987 \| 0.386061 \| \| STAT1 \| KIN001-236 \| 0.093655 \| 0.010305 \| \| STAT1 \| KIN001-244 \| 0.080738 \| 0.027887 \| \| STAT1 \| KIN001-260 \| 0.041617 \| 0.273344 \| \| STAT1 \| KIN001-266 \| 0.052867 \| 0.261946 \| \| STAT1 \| KIN001-270 \| 0.050641 \| 0.207461 \| \| STAT1 \| KU-55933 \| -0.03352 \| 0.612017 \| \| STAT1 \| LAQ824 \| 0.067728 \| 0.08768 \| \| STAT1 \| LFM-A13 \| -0.05204 \| 0.297961 \| \| STAT1 \| LY317615 \| -0.01654 \| 0.743512 \| \| STAT1 \| Lapatinib \| -0.0029 \| 0.969299 \| \| STAT1 \| Lenalidomide \| 0.032595 \| 0.691994 \| \| STAT1 \| Linifanib \| 0.060225 \| 0.212303 \| \| STAT1 \| Lisitinib \| 0.135296 \| 0.003322 \| \| STAT1 \| MG-132 \| -0.08337 \| 0.316813 \| \| STAT1 \| MK-2206 \| 0.072302 \| 0.136968 \| \| STAT1 \| MLN4924 \| -0.03574 \| 0.552816 \| \| STAT1 \| MP470 \| 0.133394 \| 0.001363 \| \| STAT1 \| MPS-1-IN-1 \| 0.097811 \| 0.007981 \| \| STAT1 \| MS-275 \| 0.024901 \| 0.803083 \| \| STAT1 \| Masitinib \| 0.068489 \| 0.069494 \| \| STAT1 \| Methotrexate \| 0.073885 \| 0.048533 \| \| STAT1 \| Midostaurin \| -0.02885 \| 0.552634 \| \| STAT1 \| Mitomycin C \| 0.032661 \| 0.553967 \| \| STAT1 \| NG-25 \| 0.107424 \| 0.002491 \| \| STAT1 \| NPK76-II-72-1 \| 0.168198 \| 9.24E-07 \| \| STAT1 \| NSC-207895 \| 0.063293 \| 0.143424 \| \| STAT1 \| NSC-87877 \| 0.027476 \| 0.653946 \| \| STAT1 \| NU-7441 \| -0.03975 \| 0.590853 \| \| STAT1 \| Navitoclax \| 0.157215 \| 1.39E-05 \| \| STAT1 \| Nilotinib \| 0.039147 \| 0.465647 \| \| STAT1 \| Nutlin-3a (-) \| -0.01082 \| 0.856697 \| \| STAT1 \| OSI-027 \| 0.077551 \| 0.032632 \| \| STAT1 \| OSI-930 \| 0.051476 \| 0.193287 \| \| STAT1 \| OSU-03012 \| -0.00122 \| 0.986831 \| \| STAT1 \| Obatoclax Mesylate \| 0.035777 \| 0.471029 \| \| STAT1 \| Olaparib \| 0.021516 \| 0.658153 \| \| STAT1 \| PAC-1 \| 0.048152 \| 0.264505 \| \| STAT1 \| PD-0325901 \| -0.06748 \| 0.09307 \| \| STAT1 \| PD-0332991 \| 0.083561 \| 0.084672 \| \| STAT1 \| PD-173074 \| -0.01493 \| 0.939238 \| \| STAT1 \| PF-4708671 \| -0.00548 \| 0.974915 \| \| STAT1 \| PF-562271 \| 0.003576 \| 0.961537 \| \| STAT1 \| PFI-1 \| -0.02966 \| 0.571445 \| \| STAT1 \| PHA-665752 \| -0.00039 \| 0.99865 \| \| STAT1 \| PHA-793887 \| 0.100556 \| 0.004191 \| \| STAT1 \| PI-103 \| 0.095441 \| 0.007896 \| \| STAT1 \| PIK-93 \| 0.09181 \| 0.009489 \| \| STAT1 \| PLX4720 \| 0.010107 \| 0.834485 \| \| STAT1 \| Paclitaxel \| -0.07257 \| 0.487509 \| \| STAT1 \| Parthenolide \| -0.00664 \| 0.962247 \| \| STAT1 \| Pazopanib \| -0.03552 \| 0.542333 \| \| STAT1 \| Phenformin \| 0.115272 \| 0.001491 \| \| STAT1 \| Pyrimethamine \| 0.042855 \| 0.727302 \| \| STAT1 \| QL-VIII-58 \| -0.0053 \| 0.960754 \| \| STAT1 \| QL-X-138 \| 0.101385 \| 0.005084 \| \| STAT1 \| QL-XI-92 \| 0.059763 \| 0.106413 \| \| STAT1 \| QL-XII-47 \| 0.02231 \| 0.61558 \| \| STAT1 \| QL-XII-61 \| 0.070685 \| 0.279418 \| \| STAT1 \| QS11 \| 0.09547 \| 0.074215 \| \| STAT1 \| RDEA119 \| -0.07372 \| 0.04529 \| \| STAT1 \| RO-3306 \| -0.08016 \| 0.05235 \| \| STAT1 \| Rapamycin \| -0.06232 \| 0.597172 \| \| STAT1 \| Roscovitine \| -0.06852 \| 0.665713 \| \| STAT1 \| Ruxolitinib \| -0.11673 \| 0.005336 \| \| STAT1 \| S-Trityl-L-cysteine \| 0.002126 \| 0.985505 \| \| STAT1 \| SB 216763 \| -0.04275 \| 0.400373 \| \| STAT1 \| SB 505124 \| 0.052628 \| 0.453373 \| \| STAT1 \| SB52334 \| 0.169632 \| 8.72E-06 \| \| STAT1 \| SB590885 \| 0.008823 \| 0.878964 \| \| STAT1 \| SGC0946 \| -0.01025 \| 0.881686 \| \| STAT1 \| SL 0101-1 \| 0.00058 \| 0.996811 \| \| STAT1 \| SN-38 \| 0.015191 \| 0.775386 \| \| STAT1 \| SNX-2112 \| 0.065017 \| 0.079055 \| \| STAT1 \| STF-62247 \| -0.03722 \| 0.379512 \| \| STAT1 \| Salubrinal \| 0.038495 \| 0.679547 \| \| STAT1 \| Saracatinib \| -0.04859 \| 0.579921 \| \| STAT1 \| Shikonin \| 0.026094 \| 0.659113 \| \| STAT1 \| Sorafenib \| 0.058447 \| 0.596485 \| \| STAT1 \| Sunitinib \| 0.029539 \| 0.73202 \| \| STAT1 \| T0901317 \| 0.079688 \| 0.044062 \| \| STAT1 \| TAE684 \| -0.07182 \| 0.500308 \| \| STAT1 \| TAK-715 \| 0.073961 \| 0.042769 \| \| STAT1 \| TG101348 \| 0.042679 \| 0.254786 \| \| STAT1 \| TGX221 \| -0.11401 \| 0.067974 \| \| STAT1 \| THZ-2-102-1 \| 0.097732 \| 0.006561 \| \| STAT1 \| THZ-2-49 \| 0.019463 \| 0.631387 \| \| STAT1 \| TL-1-85 \| 0.101716 \| 0.004496 \| \| STAT1 \| TL-2-105 \| 0.129389 \| 0.000319 \| \| STAT1 \| TPCA-1 \| 0.061892 \| 0.087161 \| \| STAT1 \| TW 37 \| 0.026263 \| 0.6096 \| \| STAT1 \| Talazoparib \| 0.032058 \| 0.50492 \| \| STAT1 \| Tamoxifen \| -0.04607 \| 0.676456 \| \| STAT1 \| Temozolomide \| -0.12262 \| 0.00869 \| \| STAT1 \| Temsirolimus \| -0.0572 \| 0.23334 \| \| STAT1 \| Thapsigargin \| 0.07926 \| 0.137376 \| \| STAT1 \| Tipifarnib \| 0.042365 \| 0.524333 \| \| STAT1 \| Tivozanib \| 0.046459 \| 0.415691 \| \| STAT1 \| Trametinib \| -0.06004 \| 0.114354 \| \| STAT1 \| Tubastatin A \| 0.153491 \| 1.05E-05 \| \| STAT1 \| UNC0638 \| 0.115053 \| 0.000909 \| \| STAT1 \| UNC1215 \| -0.0458 \| 0.368334 \| \| STAT1 \| VNLG/124 \| -0.01026 \| 0.837254 \| \| STAT1 \| VX-11e \| 0.002366 \| 0.970963 \| \| STAT1 \| VX-680 \| 0.057254 \| 0.550206 \| \| STAT1 \| VX-702 \| 0.025266 \| 0.919784 \| \| STAT1 \| Veliparib \| -0.01685 \| 0.837636 \| \| STAT1 \| Vinblastine \| 0.032974 \| 0.487091 \| \| STAT1 \| Vinorelbine \| 0.075475 \| 0.127832 \| \| STAT1 \| Vorinostat \| 0.146639 \| 4.82E-05 \| \| STAT1 \| WH-4-023 \| -0.10134 \| 0.162026 \| \| STAT1 \| WZ-1-84 \| 0.007291 \| 0.941133 \| \| STAT1 \| WZ3105 \| 0.164397 \| 2.02E-06 \| \| STAT1 \| XAV939 \| -0.07056 \| 0.069114 \| \| STAT1 \| XL-184 \| 0.083496 \| 0.050734 \| \| STAT1 \| XMD11-85h \| -0.00787 \| 0.974633 \| \| STAT1 \| XMD13-2 \| 0.101851 \| 0.004254 \| \| STAT1 \| XMD14-99 \| 0.00132 \| 0.977133 \| \| STAT1 \| XMD15-27 \| -0.03848 \| 0.433199 \| \| STAT1 \| XMD8-85 \| -0.02401 \| 0.810728 \| \| STAT1 \| XMD8-92 \| -0.01583 \| 0.916377 \| \| STAT1 \| Y-39983 \| 0.07709 \| 0.038635 \| \| STAT1 \| YK 4-279 \| -0.01316 \| 0.846981 \| \| STAT1 \| YM155 \| 0.03421 \| 0.535924 \| \| STAT1 \| YM201636 \| 0.140109 \| 7.8E-05 \| \| STAT1 \| Z-LLNle-CHO \| -0.24325 \| 4.34E-05 \| \| STAT1 \| ZG-10 \| 0.039049 \| 0.589945 \| \| STAT1 \| ZM-447439 \| 0.153345 \| 0.000144 \| \| STAT1 \| ZSTK474 \| 0.043742 \| 0.246939 \| \| STAT1 \| Zibotentan \| -0.01634 \| 0.964217 \| \| STAT1 \| piperlongumine \| -0.07082 \| 0.089616 \| \| STAT1 \| rTRAIL \| 0.041903 \| 0.570633 \| \| STAT1 \| selumetinib \| -0.07615 \| 0.039273 \| |

|  |
| --- |
